# Synthesis of 5,6-Dihydropyrazolo[5,1-*a*]isoquinolines through Tandem Reaction of *C*,*N*-Cyclic Azomethine Imines with α,β-Unsaturated Ketones

**DOI:** 10.3390/molecules28093710

**Published:** 2023-04-25

**Authors:** Young Jae Yun, Sung-Gon Kim

**Affiliations:** Department of Chemistry, Kyonggi University, 154-42, Gwanggyosan-ro, Yeongtong-gu, Suwon 16227, Republic of Korea

**Keywords:** fused pyrazoles, nitrogen heterocycles, *C*,*N*-cyclic azomethine imines, cycloaddition, tandem reaction

## Abstract

An innovative and efficient approach has been developed for the synthesis of 5,6-dihydropyrazolo[5,1-*a*]isoquinolines. This one-pot tandem reaction involves the reaction of *C*,*N*-cyclic azomethine imines with α,β-unsaturated ketones, using K_2_CO_3_ as the base and DDQ as the oxidant. The process results in functionalized 5,6-dihydropyrazolo[5,1-*a*]isoquinolines with good yields. This convenient one-step method encompasses a tandem [3 + 2]-cycloaddition, detosylation, and oxidative aromatization.

## 1. Introduction

Nitrogen-containing heterocycles are highly valued structures and play a crucial role as components in a variety of biologically significant natural products and pharmaceuticals. These molecular skeletons are found throughout nature and play a crucial role in metabolism due to their widespread occurrence as the structural nucleus in many important natural products, including hormones, antibiotics, alkaloids, and others [1,2]. In particular, pyrazoles, five-membered heterocycles with two adjacent nitrogen atoms, are recognized for their extensive range of biological properties in both natural products and their synthetic derivatives. Many FDA-approved and commercially available drugs, such as celecoxib, rimonabant, and sildenafil, have been derived from pyrazole derivatives, highlighting the wide utilization of these groups in the development of novel bioactive molecules [3,4]. Additionally, tetrahydroquinolines are a class of nitrogen-containing heterocyclic compounds that feature a benzene ring fused with a tetrahydropyridine or piperidine ring. These compounds, found naturally as well as in synthetic derivatives, exhibit a broad range of pharmacological properties, such as antibacterial, antifungal, antiviral, antimalarial, and anti-inflammatory effects. They have been largely employed for their potent antitumor properties [5,6]. Given the value of the pyrazole and tetrahydroquinoline scaffolds, the 5,6-dihydropyrazolo[5,1-*a*]isoquinoline, which is a pyrazole-fused tetrahydroquinoline, holds great potential as a target for the creation of unique biologically active compounds [7,8,9,10].

**Scheme 1:**
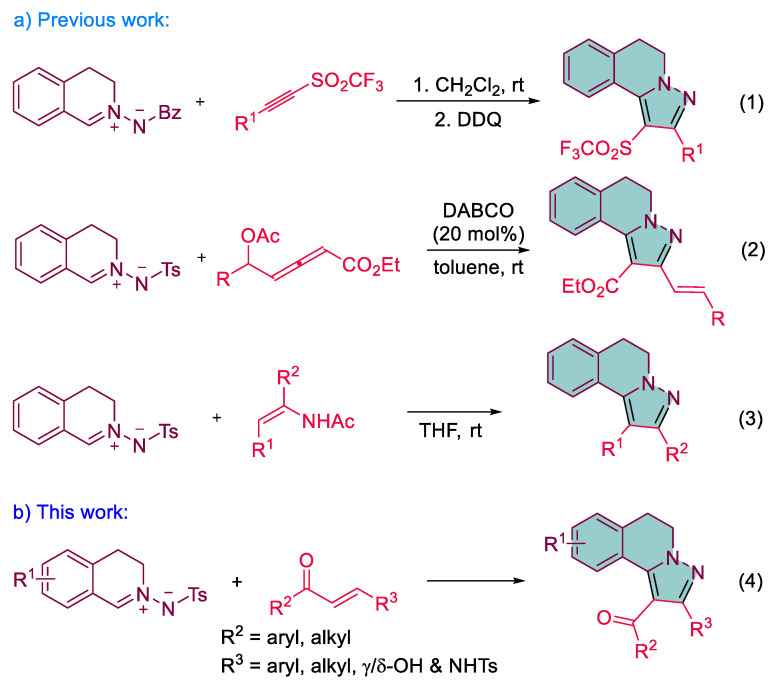
Synthesis of 5,6-dihydropyrazolo[5,1-*a*]isoquinolines from *C*,*N*-cyclic azomethine imines.

Therefore, much attention and effort have been dedicated to the improvement of efficient synthesis methods for 5,6-dihydropyrazolo[5,1-*a*]isoquinolines. Despite this, readily accessible synthetic methods for 5,6-dihydropyrazolo[5,1-*a*]isoquinolines remain scarce at present [11,12,13,14,15,16]. Several approaches for the synthesis of 5,6-dihydropyrazolo[5,1-*a*]isoquinolines from *C*,*N*-cyclic azomethine imines have been reported in the literature. The [3 + 2]-cycloaddition between *C*,*N*-cyclic azomethine imines and unsaturated compounds is a highly effective synthetic strategy for the creation of five-membered dinitrogen-fused heterocyclic compounds [17,18,19,20,21]. In 2012, Shibata et al. developed the synthesis of pyrazolo[5,1-*a*]isoquinoline triflones through a tandem 1,3-dipolar cycloaddition/oxidative aromatization between *C*,*N*-cyclic azomethine imines and triflyl alkynes (Scheme 1(1)) [11]. In 2016, Shi et al. reported the DABCO-catalyzed [3 + 2] annulation of *C*,*N*-cyclic azomethine imines and δ-acetoxylallenoates, which yielded 5,6-dihydropyrazolo[5,1-*a*]isoquinoline ethylesters (Scheme 1(2)) [12]. In 2022, Wang et al. disclosed the catalyst-free [3 + 2] annulation of *C*,*N*-cyclic azomethine imines and enamides, resulting in the synthesis of functionalized 5,6-dihydropyrazolo[5,1-*a*]isoquinolines through a tandem aza-Mannich/cyclization/aromatization (Scheme 1(3)) [16]. As part of our ongoing interest in cycloaddition reactions [22,23,24,25], we present the development of a tandem reaction of *C*,*N*-cyclic azomethine imines with α,β-unsaturated ketones, yielding highly functionalized 5,6-dihydropyrazolo[5,1-*a*]isoquinolines (Scheme 1(4)).

## 2. Results and Discussion

**Scheme 2:**
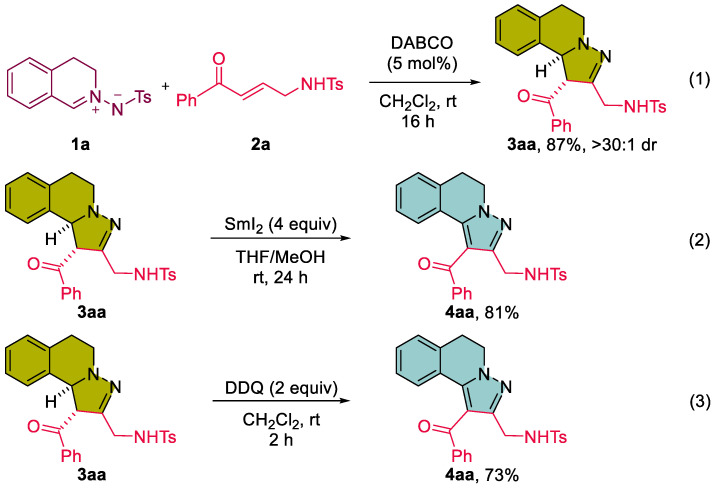
Synthesis of 5,6-dihydropyrazolo[5,1-*a*]isoquinoline **4aa** via the reaction of *C*,*N*-cyclic azomethine imine **1a** with α,β-unsaturated ketone **2a**.

Recently, we published a study on the DABCO-catalyzed cycloaddition of *N*-T-protected *C*,*N*-cyclic azomethine imines with γ-NHTs-α,β-unsaturated ketones followed by a cleavage of the tosyl group, resulting in the efficient synthesis of tetrahydropyrazolo[5,1-*a*]isoquinolines (19 examples) with high yields (up to 87% yield) and excellent diastereoselectivity (up to >30:1 dr) [24]. The reaction of *N*-T-protected *C*,*N*-cyclic azomethine imine **1a** with γ-NHTs-α,β-unsaturated ketone **2a** in the presence of the DABCO catalyst provided by tetrahydropyrazolo[5,1-*a*]isoquinoline **3aa** in an 87% yield with >30:1 dr (Scheme 2(1)). As an application of compound **3aa**, we attempted the oxidative aromatization of **3aa** using oxidants such as SmI_2_ and DDQ, which produced the corresponding dihydropyrazolo[5,1-*a*]isoquinoline **4aa** in yields of 81% and 73%, respectively (Scheme 2(2),(3)).

This led us to question whether it would be possible to synthesize **4aa** from the reaction of **1a** with **2a** in a one-pot procedure. To investigate this, we initiated our study by conducting a reaction of **1a** with **2a** in the presence of DABCO as the base catalyst and chloranil as the oxidant in CH_2_Cl_2_ (Table 1). Upon stirring for 12 h at room temperature, we were delighted to discover that the desired product **4aa** was obtained in a yield of 62% (Table 1, entry 1). Inspired by these positive results, we continued our exploration by conducting the reaction using different oxidants and bases in an effort to optimize the reaction conditions. When using other oxidants, such as DDQ and IBX, the chemical reactivity and yield were not improved (Table 1, entries 2–3). Subsequently, various organic bases (Et_3_N, *i*-Pr_2_NEt, pyridine, DBU, DBN) and inorganic bases (Na_2_CO_3_, K_2_CO_3_, Cs_2_CO_3_) were screened to optimize the reaction conditions (Table 1, entries 4–11). A slight increase in the reaction yield (71%) was achieved using *i*-Pr_2_NEt as the base. The inorganic bases were well tolerated in this reaction and K_2_CO_3_ resulted in a higher yield of the product **4aa** (75%), though a longer reaction time was required. To further optimize the reaction conditions, various organic solvents, including ClCH_2_CH_2_Cl, CHCl_3_, CH_3_CN, THF, 1,4-dioxane, toluene, and o-xylene, were screened using K_2_CO_3_ as the base and chloranil as the oxidant. Among the solvents tested, THF was found to be the optimal reaction solvent, with a slight increase in reaction temperature to 50 °C after 12 h providing the best results. The optimal reaction conditions were achieved using DDQ as the oxidant and K_2_CO_3_ as the base catalyst in THF, yielding product **4aa** with a good yield of 81% (Table 1, entry 19).



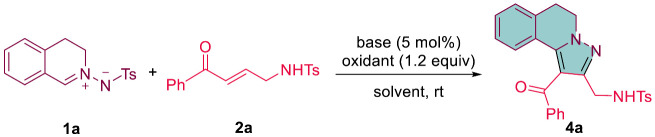



**Table 1:** Optimization of the Reaction Conditions ^a^.

Entry	Base	Oxidant	Solvent	Time (h)	Yield (%) ^b^
1	DABCO	Chloranil	CH_2_Cl_2_	12	62
2	DABCO	DDQ	CH_2_Cl_2_	24	61
3	DABCO	IBX	CH_2_Cl_2_	20	38
4	Et_3_N	Chloranil	CH_2_Cl_2_	4	17
5	*i*-Pr_2_NEt	Chloranil	CH_2_Cl_2_	4	23
6	Pyridine	Chloranil	CH_2_Cl_2_	12	71
7	DBU	Chloranil	CH_2_Cl_2_	2	24
8	DBN	Chloranil	CH_2_Cl_2_	2	26
9	Na_2_CO_3_	Chloranil	CH_2_Cl_2_	18	63
10	K_2_CO_3_	Chloranil	CH_2_Cl_2_	18	75
11	Cs_2_CO_3_	Chloranil	CH_2_Cl_2_	18	62
12	K_2_CO_3_	Chloranil	ClCH_2_ CH_2_Cl	18	45
13	K_2_CO_3_	Chloranil	CHCl_3_	18	37
14 ^c^	K_2_CO_3_	Chloranil	CH_3_CN	18	61
15 ^c^	K_2_CO_3_	Chloranil	THF	24	78
16 ^c^	K_2_CO_3_	Chloranil	1,4-Dioxane	24	68
17 ^c^	K_2_CO_3_	Chloranil	Toluene	24	52
18 ^c^	K_2_CO_3_	Chloranil	*p*-Xylene	24	73
19 ^c^	K_2_CO_3_	DDQ	THF	24	81
20 ^d^	K_2_CO_3_	DDQ	THF	24	80
21 ^e^	K_2_CO_3_	DDQ	THF	24	71
22 ^f^	K_2_CO_3_	DDQ	THF	24	65

^a^ The reactions were carried out in solvent (1.0 mL) with **1a** (0.10 mmol), **2a** (0.15 mmol), base (0.005 mmol), and oxidant (0.12 mmol) at room temperature. ^b^ Isolated yield after chromatographic purification. ^c^ Stirred at room temperature for 12 h and then heated to 50 °C and stirred for an additional 12 h. ^d^ DDQ (0.20 mmol, 2.0 equiv). ^e^ K_2_CO_3_ (0.01 mmol, 10 mol%). ^f^ K_2_CO_3_ (0.02 mmol, 20 mol%).

**Scheme 3:**
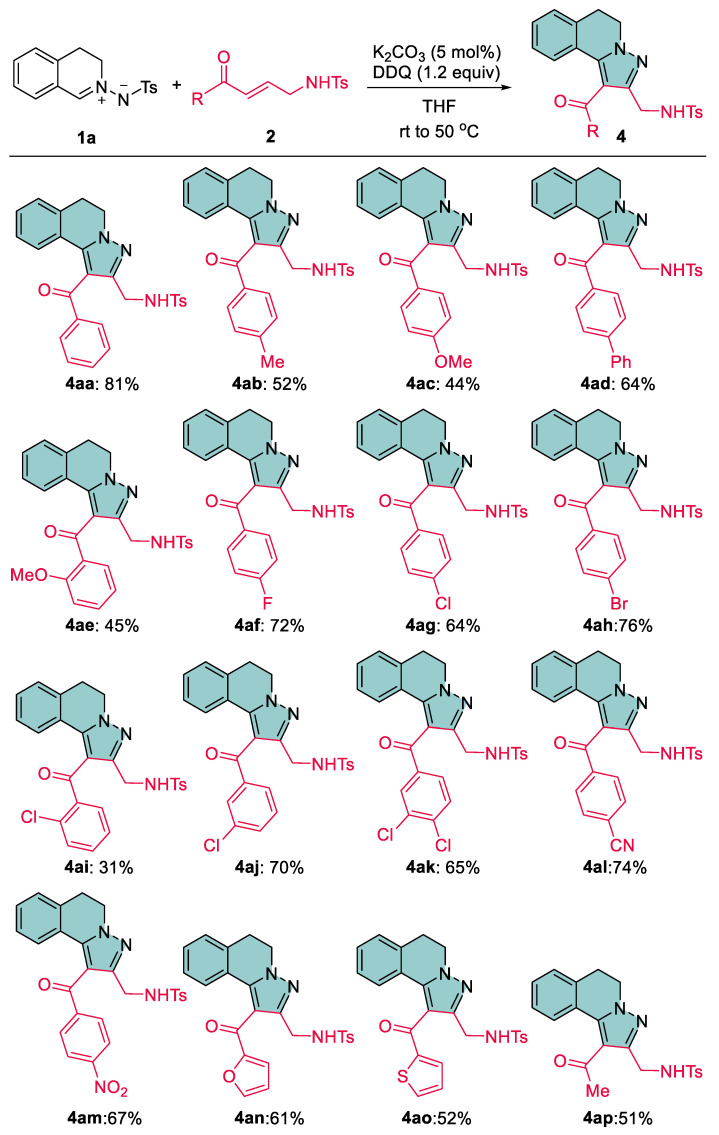
Substrate scope of γ-NHTs-α,β-unsaturated ketones ^a,b^. ^a^ Standard reaction conditions: **1a** (0.10 mmol), **2** (0.15 mmol), K_2_CO_3_ (0.005 mmol), DDQ (0.12 mmol), and THF (1.0 mL), stirred at rt for 12 h and then at 50 °C for an additional 12 h. ^b^ Isolated yield after chromatographic purification.

After optimizing the conditions, the reaction scope and versatility of γ-NHTs-α,β-unsaturated ketones were evaluated (Scheme 3). The one-pot reaction of γ-NHTs-α,β-unsaturated ketones **2a**–**m**, which possessed different electronic substituents on the aromatic ring of the enone unit, was successfully carried out, affording 5,6-dihydropyrazolo[5,1-*a*]isoquinoline products **4aa**–**am** in moderate to high yields. The electronic properties of the substituents had a subtle effect on the reaction outcome. Enones with electron-withdrawing groups at the *para*-position of the aryl ring showed higher yield efficiency than those with electron-donating substituents. *Ortho*-substituted chlorine on the enone aromatic ring resulted in a lower yield compared to the reaction with *meta*- and *para*-substituted analogs under the same reaction conditions (**4ai** vs. **4ag** and **4aj**). Additionally, γ-NHTs-α,β-unsaturated ketones bearing hetero-aromatic groups such as 2-furyl and 2-thiophenyl were well tolerated and produced the corresponding 5,6-dihydropyrazolo[5,1-*a*]isoquinoline products **4an** and **4ao** in yields of 61% and 52%, respectively. This tandem reaction was also compatible with aliphatic substituents, as demonstrated by substrate **2p**, which had a methyl group, yielding product **4ap** with an acceptable yield.

**Scheme 4:**
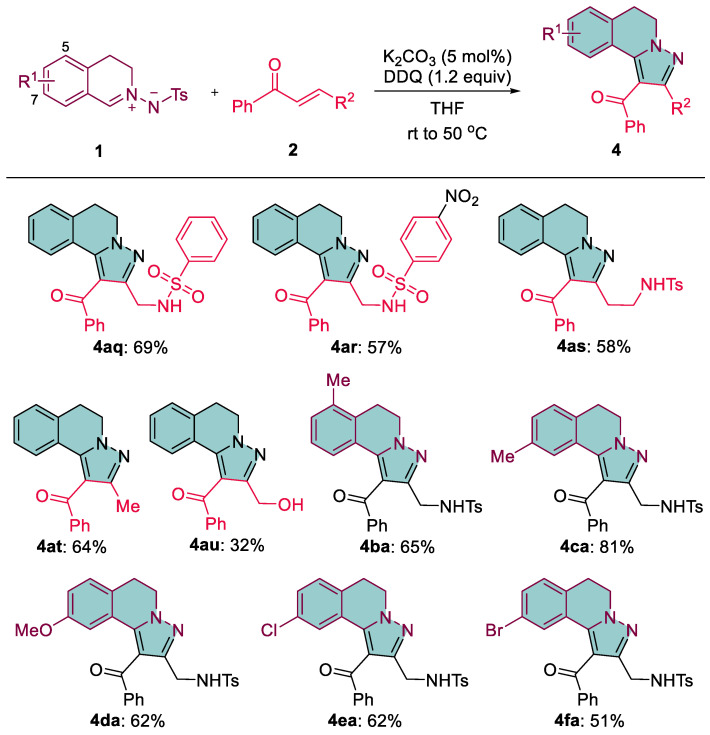
Substrate scope of β-substituted-α,β-unsaturated ketones and *C*,*N*-cyclic azomethine imines ^a,b^. ^a^ Standard reaction conditions: **1** (0.10 mmol), **2** (0.15 mmol), K_2_CO_3_ (0.005 mmol), DDQ (0.12 mmol), and THF (1.0 mL), stirred at rt for 12 h and then at 50 °C for an additional 12 h. ^b^ Isolated yield after chromatographic purification.

To expand the scope and versatility of the tandem reaction, different α,β-unsaturated ketones **2** were also examined under the optimized conditions (Scheme 4). Firstly, the impact of various arylsulfonyl protecting groups was explored. The substitution of the *p*-toluenesulfonyl group with benzenesulfonyl and *p*-nitrobenzenesulfonyl groups proved successful and resulted in good yields of the desired products **4aq** and **4ar**, at 69% and 57%, respectively. The reaction of δ-NHTs-α,β-unsaturated ketone **2s** with *N*-T-protected *C*,*N*-cyclic azomethine imine **1a** and resulted in the corresponding product **4as** in a reasonable yield. Additionally, the tandem reaction with 1-phenylbut-2-en-1-one (**2t**) was successful, producing the corresponding 5,6-dihydropyrazolo[5,1-*a*]isoquinoline product **4at** in a 65% yield. Furthermore, the reaction with γ-hydroxy-α,β-unsaturated ketone **2u** without a protecting group also proceeded, although the yield was relatively low (32%).

Finally, the substrate scope of *N*-T-protected *C*,*N*-cyclic azomethine imines **1** was also evaluated. Regardless of the electronic nature of the substituents on the aromatic ring, the reaction of *N*-T-protected *C*,*N*-cyclic azomethine imines **1** with γ-NHTs-α,β-unsaturated ketones **2a** and produced the corresponding 5,6-dihydropyrazolo[5,1-*a*]isoquinoline products **4ba**–**4fa** in good yields (51–81%). The position of the substituents on the substrate seemed to have a slight impact on the reaction yield, with C7-methyl-substituted substrate **1c** yielding the product in higher amounts compared to C5-methyl-substituted substrate **1b**.

**Scheme 5:**
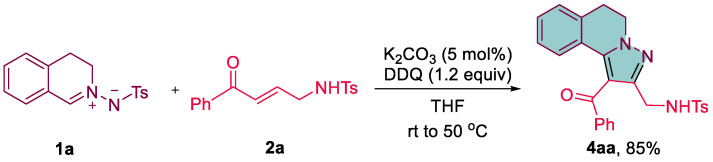
One-mmole scale synthesis of **4aa**.

To demonstrate the practical applications of this tandem reaction, the large-scale synthesis of 5,6-dihydropyrazolo[5,1-*a*]isoquinoline product **4aa** was successfully demonstrated through the one-mole-scale tandem reaction between **1a** and **2a** (Scheme 5). The reaction was conducted under the optimized conditions and resulted in a yield of 85%. This highlights the practical applications of this tandem reaction for the synthesis of biologically active compounds.

**Scheme 6:**
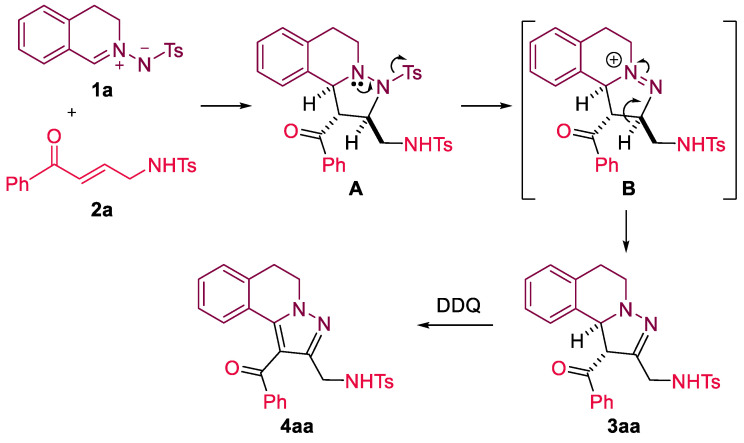
Plausible reaction mechanism.

Based on the experimental results and the previously reported literature [11,12,13,14], a plausible mechanism of the tandem reaction between *N*-T-protected *C*,*N*-cyclic azomethine imines and α,β-unsaturated ketones for the synthesis of 5,6-dihydropyrazolo[5,1-*a*]isoquinolines is proposed (Scheme 6). The reaction starts with the 1,3-dipolar cycloaddition of *C*,*N*-cyclic azomethine imine **1a** and α,β-unsaturated ketone **2a**, which produces intermediate **A**. This intermediate then undergoes elimination of the tosylate group to form intermediate **B**. Subsequently, the deprotonation of intermediate **B** generates the intermediate **3aa**, which is then subjected to oxidative aromatization using DDQ to afford the required product **4aa**.

## 3. Conclusions

In summary, we have developed an efficient and effective way to synthesize 5,6-dihydropyrazolo[5,1-*a*]isoquinolines. Combining the reaction between *C*,*N*-cyclic azomethine imines and α,β-unsaturated ketones, we have developed a one-pot procedure that includes a [3 + 2]-cycloaddition, detosylation, and oxidative aromatization. Using K_2_CO_3_ as a base and DDQ as an oxidant, we have obtained functionalized 5,6-dihydropyrazolo[5,1-*a*]isoquinolines in good yields. This method provides a convenient and straightforward approach to synthesize these compounds.

## 4. Experimental

### 4.1. General Information

Organic solvents were distilled prior to use. Organic solutions were concentrated under reduced pressure using a rotary evaporator. The chromatographic purification of products was accomplished using forced-flow chromatography on ICN 60 32–64 mesh silica gel 63. Thin-layer chromatography (TLC) was performed on EM Reagents 0.25 mm silica gel 60-F plates. The developed chromatograms were visualized by fluorescence quenching and with anisaldehyde staining. ^1^H, ^13^C{^1^H}, and ^19^F NMR spectra were recorded (400 MHz for ^1^H, 100 MHz for ^13^C, and 176 MHz for ^19^F) and were internally referenced to residual protic solvent signals. Data for ^1^H NMR are reported as follows: chemical shift (δ ppm), multiplicity (s = singlet, d = doublet, t = triplet, q = quartet, br = broad singlet, dd = doublet of doublets, dt = doublet of triplets, qd = quartet of doublets, ddd = doublet of doublet of doublets, m = multiplet), coupling constant (Hz), and integration. Data for ^13^C NMR are reported in terms of chemical shift. IR spectra were recorded on an FT IR spectrometer and are reported in wave numbers. High-resolution mass spectra (HRMS) were measured on a Q-TOF-MS with ESI using an electron impact ionization (EI-magnetic sector) mass spectrometer. γ-Sulfonamido-α,β-unsaturated ketones [26] and *C*,*N*-cyclic azomethine imines [27] were prepared according to the literature.

### 4.2. General Procedure for the Synthesis of 5,6-Dihydropyrazolo[5,1-a]isoquinolines 4

To a solution of *C*,*N*-azomethine imine **1** (0.10 mmol) and K_2_CO_3_ (0.005 mmol, 0.05 eq) in THF (1.0 mL, 0.10 M) was added α,β-unsaturated ketone **2** (0.15 mmol, 1.5 eq) at room temperature. After stirring for 10 min at room temperature, DDQ (0.12 mmol, 1.2 eq) was added. The reaction mixture was stirred for 12 h at room temperature and then heated in an oil bath at 50 °C. After stirring for 12 h, the resulting mixture was quenched with sat. NaHCO_3_ solution and the aqueous layer was extracted with EtOAc. The combined organic layer was washed with brine, dried over anhydrous Na_2_SO_4_, and concentrated in vacuo. The crude residue was purified by flash column chromatography with EtOAc/hexanes as eluent to afford desired product **4** (The spectra can be found in Supplementary Materials).

*N-((1-Benzoyl-5,6-dihydropyrazolo[5,1-a]isoquinolin-2-yl)methyl)-4-methylbenzenesulfonamide* (**4aa**). Following the general procedure; flash chromatography (EtOAc:hexane = 1:1, 37 mg, yield 81%), light-brown solid: m.p. 78–80 °C; ^1^H NMR (400 MHz, CDCl_3_) δ 7.72–7.57 (m, 4H), 7.46 (ddt, *J* = 8.8, 7.2, 1.3 Hz, 1H), 7.32–7.19 (m, 3H), 7.18–7.04 (m, 3H), 6.84 (td, *J* = 7.6, 1.4 Hz, 1H), 6.67 (dd, *J* = 7.9, 1.2 Hz, 1H), 6.24 (t, *J* = 6.4 Hz, 1H), 4.29–4.15 (m, 4H), 3.16 (t, *J* = 6.8 Hz, 2H), 2.13 (s, 3H); ^13^C{^1^H} NMR (100 MHz, CDCl_3_) δ 192.0, 149.4, 142.8, 140.4, 138.1, 137.4, 133.24, 133.19, 129.7, 129.2, 129.0, 128.6, 128.2, 128.1, 127.3, 127.0, 125.1, 114.9, 46.4, 41.1, 29.2, 21.3; IR (neat) 3255, 3058, 2922, 2853, 1684, 1596, 1576, 1540, 1507, 1486, 1425, 1305, 1278, 1224, 1091 cm^−1^; HRMS (EI) *m*/*z* calcd for [M]^+^ C_26_H_23_N_3_O_3_S: 457.1460, found: 457.1464.
*4-Methyl-N-((1-(4-methylbenzoyl)-5,6-dihydropyrazolo[5,1-a]isoquinolin-2-yl)methyl)benzenesulfonamide* (**4ab**). Following the general procedure; flash chromatography (EtOAc:hexane = 3:2, 25 mg, yield 52%), light-brown solid: m.p. 94–96 °C; ^1^H NMR (400 MHz, CDCl_3_) δ 7.67 (d, *J* = 8.3 Hz, 2H), 7.56 (d, *J* = 8.2 Hz, 2H), 7.23 (d, *J* = 7.0 Hz, 1H), 7.16 (td, *J* = 7.5, 1.2 Hz, 1H), 7.09 (dd, *J* = 8.0, 3.0 Hz, 4H), 6.88 (td, *J* = 7.8, 1.2 Hz, 1H), 6.76–6.70 (m, 1H), 6.20 (t, *J* = 6.4 Hz, 1H), 4.19 (dd, *J* = 12.6, 6.7 Hz, 4H), 3.16 (t, *J* = 6.8 Hz, 2H), 2.34 (s, 3H), 2.14 (s, 3H); ^13^C NMR (100 MHz, CDCl_3_) δ 191.7, 149.2, 144.3, 142.8, 140.1, 137.5, 135.5, 133.1, 130.0, 129.4, 129.1 (two peaks overlapping), 128.2, 128.1, 127.4, 126.9, 125.3, 115.2, 46.4, 41.0, 29.3, 21.8, 21.3.; IR (neat) 3217, 2947, 2842, 1680, 1455, 1424, 1375, 1338, 1278, 1225, 1155, 1019 cm^−1^; HRMS (EI) *m*/*z* calcd for [M]^+^ C_27_H_25_N_3_O_3_S: 471.1617, found: 471.1635.
*N-((1-(4-Methoxybenzoyl)-5,6-dihydropyrazolo[5,1-a]isoquinolin-2-yl)methyl)-4-methylbenzenesulfonamide* (**4ac**). Following the general procedure; flash chromatography (EtOAc:hexane = 3:2, 21 mg, yield 44%), light-brown solid: m.p. 80–82 °C; ^1^H NMR (400 MHz, CDCl_3_) δ 7.67 (dd, *J* = 8.5, 6.1 Hz, 4H), 7.24 (dd, *J* = 7.6, 1.2 Hz, 1H), 7.17 (td, *J* = 7.5, 1.3 Hz, 1H), 7.12–7.07 (m, 2H), 6.92 (td, *J* = 7.6, 1.4 Hz, 1H), 6.81–6.73 (m, 3H), 6.23 (t, *J* = 6.3 Hz, 1H), 4.26–4.10 (m, 4H), 3.80 (s, 3H), 3.16 (t, *J* = 6.8 Hz, 2H), 2.14 (s, 3H); ^13^C{^1^H} NMR (100 MHz, CDCl_3_) δ 190.6, 163.8, 149.1, 142.8, 139.8, 137.4, 133.1, 132.2, 130.1, 129.08, 129.06, 128.2, 128.1, 127.4, 127.1, 125.4, 115.2, 113.9, 55.6, 46.4, 41.0, 29.3, 21.3.; IR (neat) 3231, 2925, 2849, 1622, 1594, 1509, 1485, 1397, 1307, 1256, 1156, 1112, 1091, 1064, 1020 cm^−1^; HRMS (EI) *m*/*z* calcd for [M]^+^ C_27_H_25_N_3_O_4_S: 487.1566, found: 487.1545.
*N-((1-([1,1′-Biphenyl]-4-carbonyl)-5,6-dihydropyrazolo[5,1-a]isoquinolin-2-yl)methyl)-4-methylbenzenesulfonamide* (**4ad**). Following the general procedure; flash chromatography (EtOAc:hexane = 3:2, 34 mg, yield 64%), light-brown solid: m.p. 137–139 °C; ^1^H NMR (400 MHz, CDCl_3_) δ 7.77–7.64 (m, 4H), 7.59–7.49 (m, 4H), 7.47–7.41 (m, 2H), 7.40–7.34 (m, 1H), 7.24 (dd, *J* = 7.6, 1.2 Hz, 1H), 7.18–7.06 (m, 3H), 6.86 (td, *J* = 7.6, 1.3 Hz, 1H), 6.75 (dd, *J* = 7.9, 1.3 Hz, 1H), 6.28 (t, *J* = 6.4 Hz, 1H), 4.30–4.13 (m, 4H), 3.18 (t, *J* = 6.8 Hz, 2H), 2.12 (s, 3H); ^13^C{^1^H} NMR (100 MHz, CDCl_3_) δ 191.5, 149.4, 145.8, 142.8, 140.2, 139.7, 137.4, 136.7, 133.2, 130.4, 129.2, 129.1, 129.0, 128.4, 128.2, 128.2, 127.4, 127.3, 127.2, 126.9, 125.2, 115.0, 46.4, 41.1, 29.2, 21.3; IR (neat) 3269, 2922, 2854, 1627, 1535, 1484, 1454, 1425, 1316, 1227, 1155, 1091, 1059, 1019 cm^−1^; HRMS (EI) *m*/*z* calcd for [M]^+^ C_32_H_27_N_3_O_4_S: 533.1773, found: 533.1743.
*N-((1-(2-Methoxybenzoyl)-5,6-dihydropyrazolo[5,1-a]isoquinolin-2-yl)methyl)-4-methylbenzenesulfonamide* (**4ae**). Following the general procedure; flash chromatography (EtOAc:hexane = 1:1, 22 mg, yield 45%), light-brown solid: m.p. 82–84 °C; ^1^H NMR (400 MHz, CDCl_3_) δ 7.70–7.64 (m, 2H), 7.42–7.32 (m, 2H), 7.20 (dt, *J* = 7.5, 1.0 Hz, 1H), 7.17–7.07 (m, 3H), 6.92 (dd, *J* = 7.5, 0.9 Hz, 1H), 6.82 (dd, *J* = 3.8, 0.9 Hz, 2H), 6.68 (dd, *J* = 8.4, 0.9 Hz, 1H), 6.31 (t, *J* = 6.5 Hz, 1H), 4.22 (d, *J* = 6.5 Hz, 2H), 4.14 (dd, *J* = 7.2, 6.1 Hz, 2H), 3.46 (s, 3H), 3.07 (t, *J* = 6.7 Hz, 2H), 2.14 (s, 3H); ^13^C{^1^H} NMR (100 MHz, CDCl_3_) δ 190.4, 157.8, 149.7, 142.8, 141.0, 137.5, 133.4 (two peaks overlapping), 130.7, 129.2 (two peaks overlapping), 129.1, 127.7, 127.40, 127.38, 126.6, 125.4, 120.9, 116.9, 111.6, 55.4, 46.3, 41.5, 29.5, 21.3; IR (neat) 3270, 2971, 2901, 1613, 1598, 1515, 1410, 1327, 1281, 1185, 1092, 1066 cm^−1^; HRMS (EI) *m*/*z* calcd for [M]^+^ C_27_H_25_N_3_O_4_S: 487.1566, found: 487.1570.
*N-((1-(4-Fluorobenzoyl)-5,6-dihydropyrazolo[5,1-a]isoquinolin-2-yl)methyl)-4-methylbenzenesulfonamide* (**4af**). Following the general procedure; flash chromatography (EtOAc:hexane = 3:2, 34 mg, yield 72%), light-brown solid: m.p. 83–85 °C; ^1^H NMR (400 MHz, CDCl_3_) δ 7.75–7.61 (m, 4H), 7.27–7.22 (m, 1H), 7.18 (td, *J* = 7.5, 1.2 Hz, 1H), 7.13–7.07 (m, 2H), 7.00–6.86 (m, 3H), 6.67 (dd, *J* = 7.8, 1.2 Hz, 1H), 6.22 (t, *J* = 6.4 Hz, 1H), 4.28–4.11 (m, 4H), 3.17 (t, *J* = 6.8 Hz, 2H), 2.14 (s, 3H); ^13^C{^1^H} NMR (100 MHz, CDCl_3_) δ 190.4, 165.8(d, *J*^1^ = 255.4 Hz), 149.4, 142.9, 140.2, 137.4, 134.4(d, *J*^4^ = 2.9 Hz), 133.3, 132.4(d, *J*^3^ = 9.3 Hz), 129.3, 129.1, 128.3, 128.1, 127.3, 126.9, 125.0, 115.8 (d, *J*^2^ = 22.0 Hz), 114.7, 46.4, 41.0, 29.2, 21.3; ^19^F NMR (376 MHz, CDCl_3_) −105.5; IR (neat) 3131, 2922, 2858, 1626, 1596, 1505, 1456, 1401, 1328, 1278, 1224, 1154, 1092, 1065 cm^−1^; HRMS (EI) *m*/*z* calcd for [M]^+^ C_26_H_22_FN_3_O_3_S: 475.1366, found: 475.1383.
*N-((1-(4-Chlorobenzoyl)-5,6-dihydropyrazolo[5,1-a]isoquinolin-2-yl)methyl)-4-methylbenzenesulfonamide* (**4ag**). Following the general procedure; flash chromatography (EtOAc:hexane = 3:2, 32 mg, yield 64%), light-brown solid: m.p. 87–89 °C; ^1^H NMR (400 MHz, CDCl_3_) δ 7.71–7.64 (m, 2H), 7.63–7.57 (m, 2H), 7.30–7.22 (m, 3H), 7.19 (td, *J* = 7.5, 1.3 Hz, 1H), 7.14–7.07 (m, 2H), 6.91 (td, *J* = 7.6, 1.4 Hz, 1H), 6.68 (dd, *J* = 7.8, 1.2 Hz, 1H), 6.12 (t, *J* = 6.4 Hz, 1H), 4.28–4.12 (m, 4H), 3.17 (t, *J* = 6.8 Hz, 2H), 2.15 (s, 3H); ^13^C{^1^H} NMR (100 MHz, CDCl_3_) δ 190.6, 149.5, 142.9, 140.3, 139.7, 137.4, 136.4, 133.3, 131.2, 129.4, 129.1, 129.0, 128.3, 128.2, 127.4, 127.0, 125.0, 114.6, 46.4, 41.0, 29.3, 21.3; IR (neat) 3271, 3064, 2888, 1684, 1628, 1597, 1533, 1487, 1419, 1327, 1279, 1157, 1090, 1067, 1014 cm^−1^; HRMS (EI) *m*/*z* calcd for [M]^+^ C_26_H_22_ClN_3_O_3_S: 491.1070, found: 491.1047.
*N-((1-(4-Bromobenzoyl)-5,6-dihydropyrazolo[5,1-a]isoquinolin-2-yl)methyl)-4-methylbenzenesulfonamide* (**4ah**). Following the general procedure; flash chromatography (EtOAc:hexane = 3:2, 41 mg, yield 76%), light-brown solid: m.p. 90–92 °C; ^1^H NMR (400 MHz, CDCl_3_) δ 7.66 (d, *J* = 8.3 Hz, 2H), 7.57–7.49 (m, 2H), 7.45–7.39 (m, 2H), 7.27–7.23 (m, 1H), 7.18 (td, *J* = 7.5, 1.3 Hz, 1H), 7.14–7.05 (m, 2H), 6.91 (td, *J* = 7.6, 1.4 Hz, 1H), 6.67 (dd, *J* = 7.9, 1.2 Hz, 1H), 6.17 (t, *J* = 6.4 Hz, 1H), 4.19 (dd, *J* = 14.0, 6.9 Hz, 4H), 3.16 (t, *J* = 6.8 Hz, 2H), 2.14 (s, 3H); ^13^C{^1^H} NMR (100 MHz, CDCl_3_) δ 190.7, 149.4, 142.9, 140.3, 137.3, 136.8, 133.3, 131.9, 131.2, 129.4, 129.1, 128.32, 128.27, 128.1, 127.3, 126.9, 124.9, 114.5, 46.3, 40.9, 29.2, 21.3; IR (neat) 3270, 2972, 2923, 1627, 1597, 1533, 1487, 1426, 1320, 1225, 1157, 1092, 1066, 1052, 1020 cm^−1^; HRMS (EI) *m*/*z* calcd for [M]^+^ C_26_H_22_BrN_3_O_3_S: 535.0565, found: 535.0573.
*N-((1-(2-Chlorobenzoyl)-5,6-dihydropyrazolo[5,1-a]isoquinolin-2-yl)methyl)-4-methylbenzenesulfonamide* (**4ai**). Following the general procedure; flash chromatography (EtOAc:hexane = 2:3, 15 mg, yield 31%), light-brown solid: m.p. 127–129 °C; ^1^H NMR (400 MHz, CDCl_3_) δ 7.67 (d, *J* = 8.4 Hz, 2H), 7.32–7.28 (m, 2H), 7.27 (d, *J* = 3.1 Hz, 1H), 7.22–7.07 (m, 5H), 6.93–6.73 (m, 2H), 6.19 (t, *J* = 6.5 Hz, 1H), 4.29–4.12 (m, 4H), 3.08 (t, *J* = 6.7 Hz, 2H), 2.12 (s, 3H); ^13^C{^1^H} NMR (100 MHz, CDCl_3_) δ 189.9, 150.2, 142.9, 141.7, 139.0, 137.5, 133.7, 132.3, 132.2, 130.7, 130.5, 129.5, 129.1, 128.0, 127.7, 127.4, 127.0, 126.7, 124.9, 115.8, 46.4, 41.5, 29.4, 21.4; IR (neat) 3307, 2923, 2901, 1638, 1547, 1515, 1385, 1310, 1220, 1184, 1151, 1091, 1019 cm^−1^; HRMS (EI) *m*/*z* calcd for [M]^+^ C_26_H_22_ClN_3_O_3_S: 491.1070, found: 491.1096.
*N-((1-(3-Chlorobenzoyl)-5,6-dihydropyrazolo[5,1-a]isoquinolin-2-yl)methyl)-4-methylbenzenesulfonamide* (**4aj**). Following the general procedure; flash chromatography (EtOAc:hexane = 3:2, 35 mg, yield 70%), white solid: m.p. 84–86 °C; ^1^H NMR (400 MHz, CDCl_3_) δ 7.71–7.61 (m, 3H), 7.51–7.38 (m, 2H), 7.25 (dd, *J* = 7.6, 1.3 Hz, 1H), 7.22–7.16 (m, 2H), 7.11 (d, *J* = 8.0 Hz, 2H), 6.90 (td, *J* = 7.6, 1.4 Hz, 1H), 6.66 (d, *J* = 7.8 Hz, 1H), 6.13 (td, *J* = 6.4, 2.0 Hz, 1H), 4.20 (dd, *J* = 16.6, 6.6 Hz, 4H), 3.17 (t, *J* = 6.8 Hz, 2H), 2.15 (s, 3H); ^13^C{^1^H} NMR (100 MHz, CDCl_3_) δ 190.4, 149.5, 142.9, 140.5, 139.6, 137.3, 134.8, 133.3, 133.0, 129.9, 129.5, 129.4, 129.1, 128.2, 128.1, 128.0, 127.3, 126.9, 124.9, 114.4, 46.4, 41.0, 29.2, 21.3; IR (neat) 2993, 2984, 2921, 2898, 1683, 1635, 1616, 1539, 1506, 1457, 1411, 1394, 1330, 1288, 1204, 1155, 1091, 1075 cm^−1^; HRMS (EI) *m*/*z* calcd for [M]^+^ C_26_H_22_ClN_3_O_3_S: 491.1070, found: 491.1063.
*N-((1-(3,4-Dichlorobenzoyl)-5,6-dihydropyrazolo[5,1-a]isoquinolin-2-yl)methyl)-4-methylbenzenesulfonamide* (**4ak**). Following the general procedure; flash chromatography (EtOAc:hexane = 3:2, 34 mg, yield 65%), brown solid: m.p. 92–94 °C; ^1^H NMR (400 MHz, CDCl_3_) δ 7.77 (d, *J* = 2.0 Hz, 1H), 7.73–7.59 (m, 2H), 7.43 (dd, *J* = 8.3, 2.0 Hz, 1H), 7.34 (d, *J* = 8.3 Hz, 1H), 7.30–7.25 (m, 1H), 7.21 (td, *J* = 7.5, 1.2 Hz, 1H), 7.12 (d, *J* = 8.0 Hz, 2H), 6.94 (td, *J* = 7.6, 1.5 Hz, 1H), 6.67 (dd, *J* = 7.8, 1.2 Hz, 1H), 6.09 (t, *J* = 6.4 Hz, 1H), 4.33–4.06 (m, 4H), 3.18 (t, *J* = 6.8 Hz, 2H), 2.16 (s, 3H); ^13^C{^1^H} NMR (100 MHz, CDCl_3_) δ 189.2, 149.5, 143.0, 140.4, 137.62, 137.57, 137.2, 133.4, 133.2, 131.5, 130.6, 129.6, 129.1, 129.0, 128.4, 128.1, 127.3, 127.0, 124.8, 114.1, 46.4, 40.9, 29.2, 21.3; IR (neat) 3268, 2968, 2901, 1641, 1598, 1549, 1486, 1415, 1396, 1328, 1220, 1186, 1158, 1066, 1020 cm^−1^; HRMS (EI) *m*/*z* calcd for [M]^+^ C_26_H_21_Cl_2_N_3_O_3_S_1_: 525.0681, found: 525.0674.
*N-((1-(4-Cyanobenzoyl)-5,6-dihydropyrazolo[5,1-a]isoquinolin-2-yl)methyl)-4-methylbenzenesulfonamide* (**4al**). Following the general procedure; flash chromatography (EtOAc:hexane = 3:2, 36 mg, yield 74%), light-brown solid: m.p. 107–109 °C; ^1^H NMR (400 MHz, CDCl_3_) δ 7.76–7.70 (m, 2H), 7.71–7.65 (m, 2H), 7.61–7.54 (m, 2H), 7.31–7.23 (m, 1H), 7.20 (td, *J* = 7.5, 1.3 Hz, 1H), 7.15–7.09 (m, 2H), 6.86 (td, *J* = 7.6, 1.4 Hz, 1H), 6.54 (dd, *J* = 7.9, 1.2 Hz, 1H), 6.11 (t, *J* = 6.5 Hz, 1H), 4.21 (dd, *J* = 14.1, 6.9 Hz, 4H), 3.18 (t, *J* = 6.8 Hz, 2H), 2.15 (s, 3H); ^13^C{^1^H} NMR (100 MHz, CDCl_3_) δ 190.1, 149.9, 143.0, 141.5, 140.7, 137.3, 133.5, 132.4, 130.1, 129.7, 129.1, 128.4, 128.2, 127.3, 126.9, 124.6, 117.9, 116.1, 114.1, 46.4, 40.9, 29.2, 21.3; IR (neat) 3252, 2921, 2856, 2228, 1631, 1601, 1533, 1486, 1455, 1401, 1316, 1277, 1221, 1155, 1091, 1065, 1018 cm^−1^; HRMS (EI) *m*/*z* calcd for [M]^+^ C_27_H_22_N_4_O_3_S: 482.1413, found: 482.1402.
4-Methyl-N-((1-(4-nitrobenzoyl)-5,6-dihydropyrazolo[5,1-a]isoquinolin-2-yl)methyl)benzenesulfonamide (**4am**). Following the general procedure; flash chromatography (EtOAc:hexane = 3:2, 34 mg, yield 67%), light-brown solid: m.p. 109–111 °C; ^1^H NMR (400 MHz, CDCl_3_) δ 8.15–8.09 (m, 2H), 7.85–7.76 (m, 2H), 7.71–7.63 (m, 2H), 7.28–7.25 (m, 1H), 7.19 (td, *J* = 7.5, 1.3 Hz, 1H), 7.16–7.08 (m, 2H), 6.85 (td, *J* = 7.6, 1.3 Hz, 1H), 6.57 (dd, *J* = 7.9, 1.2 Hz, 1H), 6.11 (t, *J* = 6.4 Hz, 1H), 4.31–4.14 (m, 4H), 3.19 (t, *J* = 6.8 Hz, 2H), 2.16 (s, 3H); ^13^C{^1^H} NMR (100 MHz, CDCl_3_) δ 189.9, 150.1, 149.9, 143.09, 143.06, 140.8, 137.3, 133.5, 130.7, 129.8, 129.2, 128.5, 128.2, 127.3, 126.9, 124.6, 123.8, 114.2, 46.4, 40.9, 29.1, 21.3; IR (neat) 2966, 2921, 1642, 1598, 1548, 1520, 1486, 1470, 1415, 1342, 1312, 1277, 1220, 1155, 1091, 1065, 1018 cm^−1^; HRMS (EI) *m*/*z* calcd for [M]^+^ C_26_H_22_N_4_O_5_S: 502.1311, found: 502.1329.
*N-((1-(Furan-2-carbonyl)-5,6-dihydropyrazolo[5,1-a]isoquinolin-2-yl)methyl)-4-methylbenzenesulfonamide* (**4an**). Following the general procedure; flash chromatography (EtOAc:hexane = 3:2, 27 mg, yield 61%), light-brown solid: m.p. 84–86 °C; ^1^H NMR (400 MHz, CDCl_3_) δ 7.67 (d, *J* = 8.3 Hz, 2H), 7.35 (dd, *J* = 1.7, 0.8 Hz, 1H), 7.29–7.20 (m, 2H), 7.10–7.00 (m, 4H), 6.80 (dd, *J* = 7.8, 1.2 Hz, 1H), 6.44 (dd, *J* = 3.6, 1.7 Hz, 1H), 6.26 (t, *J* = 6.5 Hz, 1H), 4.25 (d, *J* = 6.5 Hz, 2H), 4.16 (dd, *J* = 7.4, 6.3 Hz, 2H), 3.17 (t, *J* = 6.8 Hz, 2H), 2.09 (s, 3H); ^13^C{^1^H} NMR (100 MHz, CDCl_3_) δ 178.4, 152.5, 149.2, 146.8, 142.8, 140.4, 137.5, 133.1, 129.2, 129.0, 128.2, 127.4, 127.2 (two peaks overlapping), 125.8, 120.0, 114.3, 112.8, 46.4, 41.0, 29.2, 21.3; IR (neat) 3261, 2971, 2924, 1613, 1581, 1535, 1462, 1393, 1320, 1282, 1156, 1091, 1066, 1017 cm^−1^; HRMS (EI) *m*/*z* calcd for [M]^+^ C_24_H_21_N_3_O_4_S: 447.1253, found: 447.1270.
*4-Methyl-N-((1-(thiophene-2-carbonyl)-5,6-dihydropyrazolo[5,1-a]isoquinolin-2-yl)methyl)benzenesulfonamide* (**4ao**). Following the general procedure; flash chromatography (EtOAc:hexane = 3:2, 24 mg, yield 52%), light-brown solid: m.p. 89–91 °C; ^1^H NMR (400 MHz, CDCl_3_) δ 7.71–7.64 (m, 2H), 7.62 (dd, *J* = 4.9, 1.2 Hz, 1H), 7.30–7.23 (m, 2H), 7.21 (td, *J* = 7.4, 1.3 Hz, 1H), 7.13–7.05 (m, 2H), 7.01 (td, *J* = 7.6, 1.4 Hz, 1H), 6.91 (ddd, *J* = 14.3, 6.4, 2.5 Hz, 2H), 6.15 (t, *J* = 6.4 Hz, 1H), 4.24 (d, *J* = 6.4 Hz, 2H), 4.21–4.13 (m, 2H), 3.17 (t, *J* = 6.8 Hz, 2H); ^13^C{^1^H} NMR (100 MHz, CDCl_3_) δ 183.6, 148.8, 144.0, 142.8, 139.7, 137.4, 135.2, 134.8, 133.1, 129.2, 129.0, 128.3, 128.2, 127.9, 127.4, 127.1, 125.4, 115.1, 46.4, 40.9, 29.2, 21.3; IR (neat) 3253, 2971, 2924, 1613, 1565, 1471, 1463, 1409, 1326, 1281, 1185, 1091, 1045 cm^−1^; HRMS (EI) *m*/*z* calcd for [M]^+^ C_24_H_21_N_3_O_3_S_2_: 463.1024, found: 463.1028.
*N-((1-Acetyl-5,6-dihydropyrazolo[5,1-a]isoquinolin-2-yl)methyl)-4-methylbenzenesulfonamide* (**4ap**). Following the general procedure; flash chromatography (EtOAc:hexane = 1:1, 20 mg, yield 51%), light-brown solid: m.p. 67–69 °C; ^1^H NMR (400 MHz, CDCl_3_) δ 7.69–7.56 (m, 2H), 7.42–7.32 (m, 4H), 7.14–7.02 (m, 2H), 6.21 (t, *J* = 6.5 Hz, 1H), 4.24 (d, *J* = 6.5 Hz, 2H), 4.16–4.07 (m, 2H), 3.11 (t, *J* = 6.7 Hz, 2H), 2.42 (s, 3H), 2.13 (s, 3H); ^13^C{^1^H} NMR (100 MHz, CDCl_3_) δ 196.3, 148.7, 142.9, 140.6, 137.5, 134.1, 129.9, 129.0, 128.5, 128.0, 127.5, 127.4, 125.5, 117.6, 46.2, 41.5, 30.2, 29.4, 21.3.; IR (neat) 2987, 2902, 1659, 1642, 1530, 1453, 1558, 1405, 1395, 1209, 1089, 1079, 1019 cm^−1^; HRMS (EI) *m*/*z* calcd for [M]^+^ C_21_H_21_N_3_O_3_S: 395.1304, found: 395.1279.
*N-((1-Benzoyl-5,6-dihydropyrazolo[5,1-a]isoquinolin-2-yl)methyl)benzenesulfonamide* (**4aq**). Following the general procedure; flash chromatography (EtOAc:hexane = 3:2, 31 mg, yield 69%), light-brown solid: m.p. 88–90 °C; ^1^H NMR (400 MHz, CDCl_3_) δ 7.85–7.78 (m, 2H), 7.68–7.61 (m, 2H), 7.51–7.42 (m, 1H), 7.38–7.31 (m, 2H), 7.35–7.25 (m, 3H), 7.25–7.19 (m, 1H), 7.15 (td, *J* = 7.5, 1.3 Hz, 1H), 6.83 (td, *J* = 7.6, 1.4 Hz, 1H), 6.69 (dd, *J* = 7.9, 1.2 Hz, 1H), 6.27 (t, *J* = 6.3 Hz, 1H), 4.24 (d, *J* = 6.4 Hz, 2H), 4.16 (dd, *J* = 7.3, 6.3 Hz, 2H), 3.15 (t, *J* = 6.8 Hz, 2H); ^13^C{^1^H} NMR (100 MHz, CDCl_3_) δ 192.1, 149.4, 140.4, 140.3, 138.0, 133.3, 133.2, 132.1, 129.8, 129.2, 128.6, 128.6, 128.23, 128.16, 127.3, 126.8, 125.1, 115.0, 46.3, 41.0, 29.3; IR (neat) 2987, 2901, 1640, 1628, 1596, 1531, 1485, 1446, 1404, 1318, 1278, 1224, 1090, 1056 cm^−1^; HRMS (EI) *m*/*z* calcd for [M]^+^ C_25_H_21_N_3_O_3_S: 443.1304, found: 443.1292.
*N-((1-Benzoyl-5,6-dihydropyrazolo[5,1-a]isoquinolin-2-yl)methyl)-4-nitrobenzenesulfonamide* (**4ar**). Following the general procedure; flash chromatography (EtOAc:hexane = 3:2, 28 mg, yield 57%), light-brown solid: m.p. 103–105 °C; ^1^H NMR (400 MHz, CDCl_3_) δ 8.19–8.05 (m, 2H), 8.03–7.92 (m, 2H), 7.68–7.56 (m, 2H), 7.52–7.42 (m, 1H), 7.31–7.25 (m, 2H), 7.22 (dd, *J* = 7.7, 1.3 Hz, 1H), 7.15 (td, *J* = 7.5, 1.2 Hz, 1H), 6.81 (td, *J* = 7.6, 1.4 Hz, 1H), 6.63–6.49 (m, 2H), 4.32 (d, *J* = 6.4 Hz, 2H), 4.22–4.10 (m, 2H), 3.16 (t, *J* = 6.8 Hz, 2H); ^13^C{^1^H} NMR (100 MHz, CDCl_3_) δ 192.2, 149.6, 149.3, 146.5, 140.6, 137.8, 133.5, 133.1, 129.8, 129.5, 128.74, 128.72, 128.3, 128.2, 127.0, 124.7, 123.6, 114.9, 46.5, 41.1, 29.2; IR (neat) 2988, 2885, 1642, 1608, 1527, 1486, 1403, 1346, 1311, 1278, 1224, 1162, 1057, 1027 cm^−1^; HRMS (EI) *m*/*z* calcd for [M]^+^ C_25_H_20_N_4_O_5_S: 488.1154, found: 488.1136.
*N-(2-(1-Benzoyl-5,6-dihydropyrazolo[5,1-a]isoquinolin-2-yl)ethyl)-4-methylbenzenesulfonamide* (**4as**). Following the general procedure; flash chromatography (EtOAc:hexane = 3:2, 27 mg, yield 58%), light-brown solid: m.p. 115–117 °C; ^1^H NMR (400 MHz, CDCl_3_) δ 7.79–7.64 (m, 4H), 7.54–7.46 (m, 1H), 7.39–7.29 (m, 2H), 7.28–7.23 (m, 1H), 7.18 (ddd, *J* = 7.6, 5.2, 3.4 Hz, 1H), 7.12 (d, *J* = 8.1 Hz, 2H), 6.98–6.88 (m, 2H), 6.29 (t, *J* = 5.2 Hz, 1H), 4.27–4.19 (m, 2H), 3.32 (dt, *J* = 6.6, 5.3 Hz, 2H), 3.21 (t, *J* = 6.9 Hz, 2H), 2.73 (dd, *J* = 6.6, 5.4 Hz, 2H), 2.25 (s, 3H); ^13^C{^1^H} NMR (100 MHz, CDCl_3_) δ 192.4, 150.7, 142.8, 139.4, 138.2, 137.3, 133.4, 133.0, 130.0, 129.5, 129.1, 128.7, 128.2, 127.7, 127.07, 127.05, 125.4, 115.7, 46.4, 42.8, 29.3, 26.7, 21.5; IR (neat) 3276, 3057, 2923, 1638, 1596, 1579, 1536, 1489, 1470, 1409, 1339, 1312, 1303, 1224, 1200, 1119, 1080, 1047 cm^−1^; HRMS (EI) *m*/*z* calcd for [M]^+^ C_27_H_25_N_3_O_3_S: 471.1617, found: 471.1617.
*(2-Methyl-5,6-dihydropyrazolo[5,1-a]isoquinolin-1-yl)(phenyl)methanone* (**4at**). Following the general procedure; flash chromatography (EtOAc:hexane = 2:3, 18 mg, yield 64%), white solid: m.p. 75–77 °C; ^1^H NMR (400 MHz, CDCl_3_) δ 7.89–7.79 (m, 2H), 7.58–7.49 (m, 1H), 7.44–7.37 (m, 2H), 7.36–7.31 (m, 1H), 7.26–7.17 (m, 2H), 7.04 (td, *J* = 7.6, 1.5 Hz, 1H), 4.32 (t, *J* = 6.9 Hz, 2H), 3.22 (t, *J* = 6.8 Hz, 2H), 2.19 (s, 3H); ^13^C NMR (100 MHz, CDCl_3_) δ 193.1, 149.4, 139.5, 139.0, 133.08, 133.06, 129.8, 128.9, 128.7, 128.2, 127.2, 127.0, 126.0, 116.1, 46.4, 29.5, 13.7; IR (neat) 2972, 2901, 1612, 1596, 1578, 1531, 1487, 1477, 1423, 1394, 1349, 1340, 1314, 1276, 1254, 1195, 1137, 1026 cm^−1^; HRMS (EI) *m*/*z* calcd for [M]^+^ C_19_H_16_N_2_O: 288.1263, found: 288.1260.
*(2-(Hydroxymethyl)-5,6-dihydropyrazolo[5,1-a]isoquinolin-1-yl)(phenyl)methanone* (**4au**). Following the general procedure; flash chromatography (EtOAc:hexane = 3:2, 10 mg, yield 32%), light-brown gum; ^1^H NMR (400 MHz, CDCl_3_) δ 7.83–7.71 (m, 2H), 7.54–7.43 (m, 1H), 7.36–7.28 (m, 2H), 7.26–7.22 (m, 1H), 7.16 (td, *J* = 7.3, 1.6 Hz, 1H), 6.91–6.76 (m, 2H), 4.73–4.62 (m, 2H), 4.40–4.27 (m, 2H), 3.92 (s, 1H), 3.23 (t, *J* = 6.8 Hz, 2H); ^13^C{^1^H} NMR (100 MHz, CDCl_3_) δ 192.9, 154.5, 140.6, 138.3, 133.4, 133.3, 129.9, 129.2, 128.7, 128.5, 128.2, 126.9, 125.4, 115.6, 58.5, 46.6, 29.5; IR (neat) 3384, 3059, 2923, 1643, 1596, 1577, 1536, 1486, 1463, 1426, 1403, 1320, 1278, 1224, 1199 1138, 1094, 1026 cm^−1^; HRMS (EI) *m*/*z* calcd for [M]^+^ C_19_H_16_N_2_O_2_: 304.1212, found: 304.1223.
*N-((1-Benzoyl-7-methyl-5,6-dihydropyrazolo[5,1-a]isoquinolin-2-yl)methyl)-4-methylbenzenesulfonamide* (**4ba**). Following the general procedure; flash chromatography (EtOAc:hexane = 1:1, 31 mg, yield 65%), light-brown solid: m.p. 112–114 °C; ^1^H NMR (400 MHz, CDCl_3_) δ 7.72–7.57 (m, 4H), 7.44 (ddt, *J* = 8.7, 7.1, 1.3 Hz, 1H), 7.32–7.21 (m, 2H), 7.16–7.06 (m, 2H), 7.02 (dt, *J* = 7.6, 1.1 Hz, 1H), 6.72 (t, *J* = 7.7 Hz, 1H), 6.53 (dd, *J* = 8.0, 1.3 Hz, 1H), 6.24 (t, *J* = 6.4 Hz, 1H), 4.27–4.08 (m, 4H), 3.11 (t, *J* = 6.8 Hz, 2H), 2.33 (s, 3H), 2.15 (s, 3H); ^13^C{^1^H} NMR (100 MHz, CDCl_3_) δ 192.2, 149.5, 142.8, 140.6, 138.1, 137.4 135.5, 133.2, 131.7, 131.0, 129.8, 129.1, 128.6, 127.4, 126.4, 126.3, 125.0, 115.0, 46.0, 41.1, 25.4, 21.4, 19.8.; IR (neat) 3262, 2971, 2922, 1627, 1597, 1577, 1531, 1448, 1321, 1254, 1236, 1091, 1066, 1058 cm^−1^; HRMS (EI) *m*/*z* calcd for [M]^+^ C_27_H_25_N_3_O_3_S: 471.1617, found: 471.1603.
*N-((1-Benzoyl-9-methyl-5,6-dihydropyrazolo[5,1-a]isoquinolin-2-yl)methyl)-4-methylbenzenesulfonamide* (**4ca**). Following the general procedure; flash chromatography (EtOAc:hexane = 1:1, 38 mg, yield 81%), light-brown solid: m.p. 83–85 °C; ^1^H NMR (400 MHz, CDCl_3_) δ 7.72–7.65 (m, 2H), 7.65–7.58 (m, 2H), 7.46 (ddt, *J* = 8.7, 7.1, 1.3 Hz, 1H), 7.28 (td, *J* = 7.4, 1.4 Hz, 2H), 7.10 (ddd, *J* = 7.6, 3.0, 2.0 Hz, 3H), 6.94 (ddd, *J* = 7.7, 1.8, 0.9 Hz, 1H), 6.41–6.36 (m, 1H), 6.30 (t, *J* = 6.5 Hz, 1H), 4.25 (d, *J* = 6.5 Hz, 2H), 4.16 (dd, *J* = 7.3, 6.3 Hz, 2H), 3.10 (t, *J* = 6.8 Hz, 2H), 2.14 (s, 3H), 1.88 (s, 3H); ^13^C{^1^H} NMR (100 MHz, CDCl_3_) δ 192.2, 149.7, 142.8, 140.7, 138.5, 137.5, 136.4, 133.0, 130.2, 129.9, 129.7, 129.3, 129.1, 128.6, 127.8, 127.4, 124.8, 114.9, 46.6, 41.2, 28.8, 21.3, 20.8; IR (neat) 2920, 2853, 1623, 1596, 1576, 1533, 1494, 1454, 1396, 1319, 1280, 1232, 1195, 1185, 1156, 1092, 1064 cm^−1^; HRMS (EI) *m*/*z* calcd for [M]^+^ C_27_H_25_N_3_O_3_S: 471.1617, found: 471.1631.
*N-((1-Benzoyl-9-methoxy-5,6-dihydropyrazolo[5,1-a]isoquinolin-2-yl)methyl)-4-methylbenzenesulfonamide* (**4da**). Following the general procedure; flash chromatography (EtOAc:hexane = 1:1, 30 mg, yield 62%), light-brown solid: m.p. 116–118 °C; ^1^H NMR (400 MHz, CDCl_3_) δ 7.73–7.64 (m, 4H), 7.48 (ddt, *J* = 8.7, 7.3, 1.3 Hz, 1H), 7.34–7.28 (m, 2H), 7.12 (dd, *J* = 8.2, 3.6 Hz, 3H), 6.71 (dd, *J* = 8.3, 2.6 Hz, 1H), 6.26–6.13 (m, 2H), 4.24 (d, *J* = 6.3 Hz, 2H), 4.17 (dd, *J* = 7.4, 6.3 Hz, 2H), 3.24 (s, 3H), 3.10 (t, *J* = 6.8 Hz, 2H), 2.17 (s, 3H); ^13^C{^1^H} NMR (100 MHz, CDCl_3_) δ 191.8, 158.1, 149.7, 142.9, 140.3, 138.2, 137.5, 133.4, 129.8, 129.1 (two peaks overlapping), 128.8, 127.4, 125.9, 125.3, 116.7, 114.9, 112.5, 55.1, 46.8, 41.0, 28.4, 21.3; IR (neat) 3266, 2954, 2926, 1614, 1597, 1577, 1535, 1495, 1455, 1399, 1320, 1244, 1212, 1183, 1156, 1092, 1065, 1027 cm^−1^; HRMS (EI) *m*/*z* calcd for [M]^+^ C_27_H_25_N_3_O_4_S: 487.1566, found: 487.1567.
*N-((1-Benzoyl-9-chloro-5,6-dihydropyrazolo[5,1-a]isoquinolin-2-yl)methyl)-4-methylbenzenesulfonamide* (**4ea**). Following the general procedure; flash chromatography (EtOAc:hexane = 3:2, 31 mg, yield 62%), light-brown solid: m.p. 86–88 °C; ^1^H NMR (400 MHz, CDCl_3_) δ 7.70–7.59 (m, 4H), 7.56–7.48 (m, 1H), 7.37–7.29 (m, 2H), 7.20–7.06 (m, 4H), 6.61 (d, *J* = 2.1 Hz, 1H), 6.19 (t, *J* = 6.4 Hz, 1H), 4.27–4.14 (m, 4H), 3.13 (t, *J* = 6.8 Hz, 2H), 2.17 (s, 3H); ^13^C{^1^H} NMR (100 MHz, CDCl_3_) δ 191.9, 149.7, 142.9, 139.1, 138.2, 137.4, 133.5, 132.7, 131.5, 129.5, 129.3, 129.1, 129.0, 128.8, 128.1, 127.4, 126.6, 115.5, 46.3, 41.0, 28.8, 21.3; IR (neat) 3263, 2921, 1628, 1597, 1533, 1452, 1403, 1318, 1225, 1106, 1091, 1065, 1051 cm^−1^; HRMS (EI) *m*/*z* calcd for [M]^+^ C_26_H_22_ClN_3_O_3_S: 491.1070, found: 491.1083.
*N-((1-Benzoyl-9-bromo-5,6-dihydropyrazolo[5,1-a]isoquinolin-2-yl)methyl)-4-methylbenzenesulfonamide* (**4fa**). Following the general procedure; flash chromatography (EtOAc:hexane = 3:2, 27 mg, yield 51%), light-brown solid: m.p. 90–92 °C; ^1^H NMR (400 MHz, CDCl_3_) δ 7.64 (ddd, *J* = 21.9, 7.5, 1.6 Hz, 4H), 7.55–7.48 (m, 1H), 7.36–7.29 (m, 2H), 7.27–7.22 (m, 1H), 7.11 (dd, *J* = 8.1, 3.2 Hz, 3H), 6.74 (d, *J* = 2.0 Hz, 1H), 6.21 (t, *J* = 6.4 Hz, 1H), 4.28–4.10 (m, 4H), 3.11 (t, *J* = 6.8 Hz, 2H), 2.16 (s, 3H); ^13^C{^1^H} NMR (100 MHz, CDCl_3_) δ 191.9, 149.8, 142.9, 139.0, 138.2, 137.4, 133.5, 132.0, 131.9, 131.1, 129.6, 129.5, 129.1, 128.8, 127.4, 126.9, 120.5, 115.5, 46.3, 41.1, 28.8, 21.3; IR (neat) 3269, 2922, 1629, 1596, 1577, 1531, 1453, 1319, 1225, 1158, 1092, 1066 cm^−1^; HRMS (EI) *m*/*z* calcd for [M]^+^ C_26_H_22_BrN_3_O_3_S: 535.0565, found: 535.0557.

## Data Availability

Not applicable.

## Supplementary Materials

The following supporting information can be downloaded at https://www.mdpi.com/article/10.3390/molecules28093710/s1, NMR spectra of the products (**4aa**–**4fa**).

## Sample Availability

Samples of all obtained compounds are available from the authors.
