# Supplementary material for: Synthesis of 5,6-Dihydropyrazolo[5,1-a]isoquinolines through Tandem Reaction of C,N-Cyclic Azomethine Imines with α,β-Unsaturated Ketones
Source: Molecules. 2023 Apr 25;28(9):3710. doi: 10.3390/molecules28093710 (PMC10180063; doi:10.3390/molecules28093710)

Supporting Information

Synthesis of 5,6-Dihydropyrazolo[5,1-a]isoquinolines through Tandem Reaction of C,N-Cyclic Azomethine Imines with α,β-Unsaturated Ketones

Young Jae Yun and Sung-Gon Kim *

Department of Chemistry, Kyonggi University, 154-42, Gwanggyosan-ro, Yeongtong-gu, Suwon 16227, Republic of Korea
* Correspondence: sgkim123@kyonggi.ac.kr

^{1}H NMR (400 MHz) in $CDCl_3$

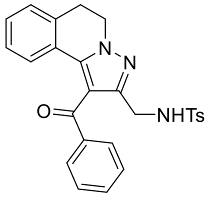

4aa

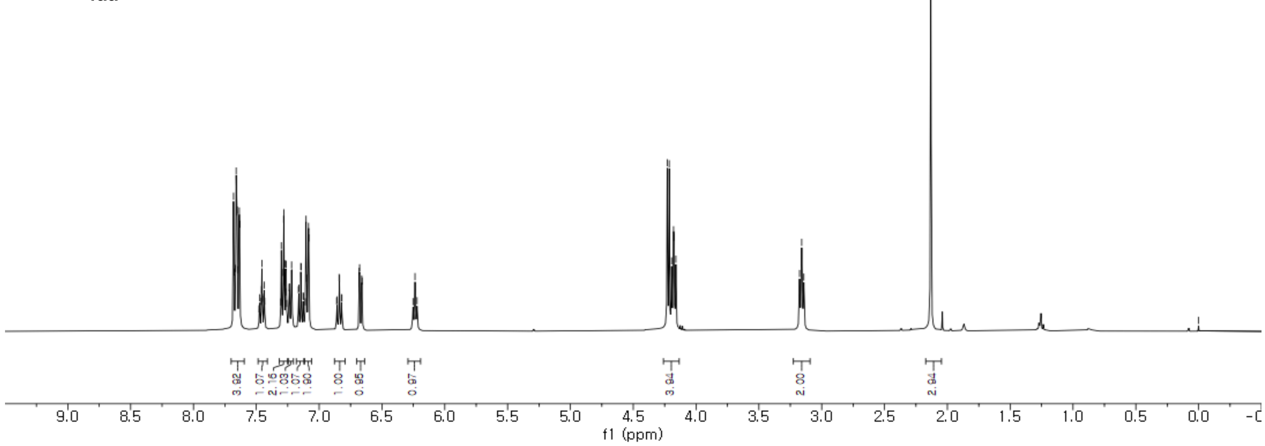

^{13}C NMR (100 MHz) in $CDCl_3$

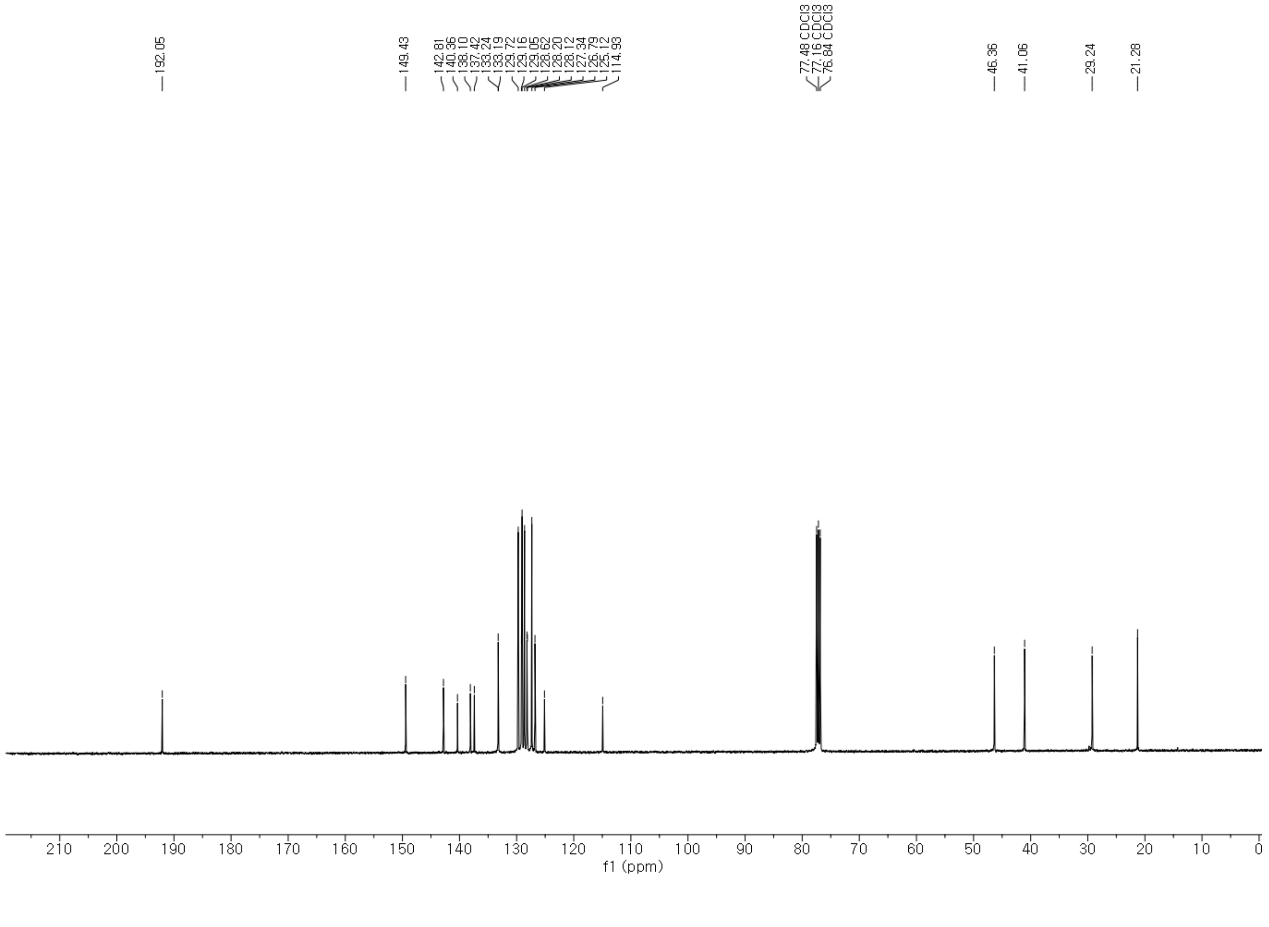

^{1}H NMR (400 MHz) in $CDCl_3$

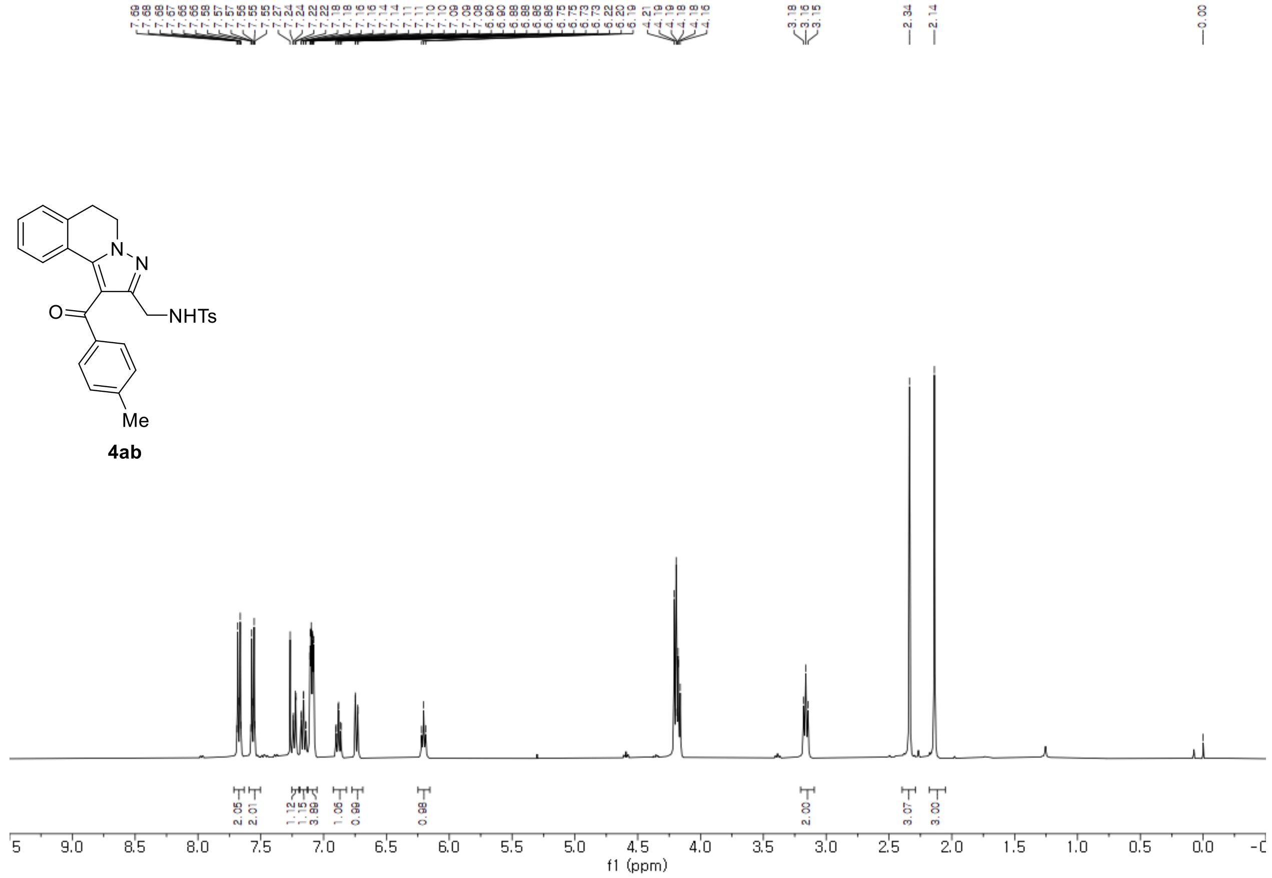

^{13}C NMR (100 MHz) in $CDCl_3$

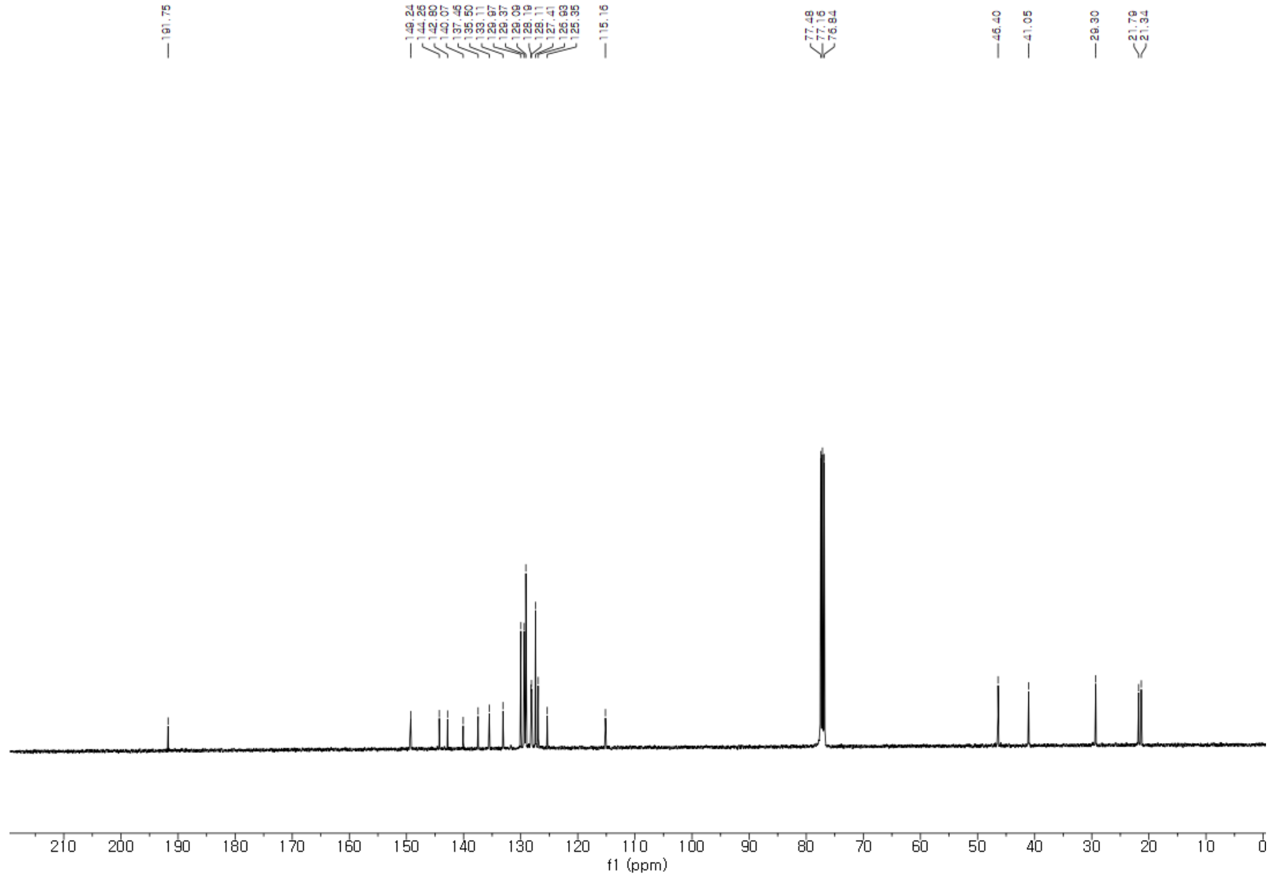

^{1}H NMR (400 MHz) in $CDCl_3$

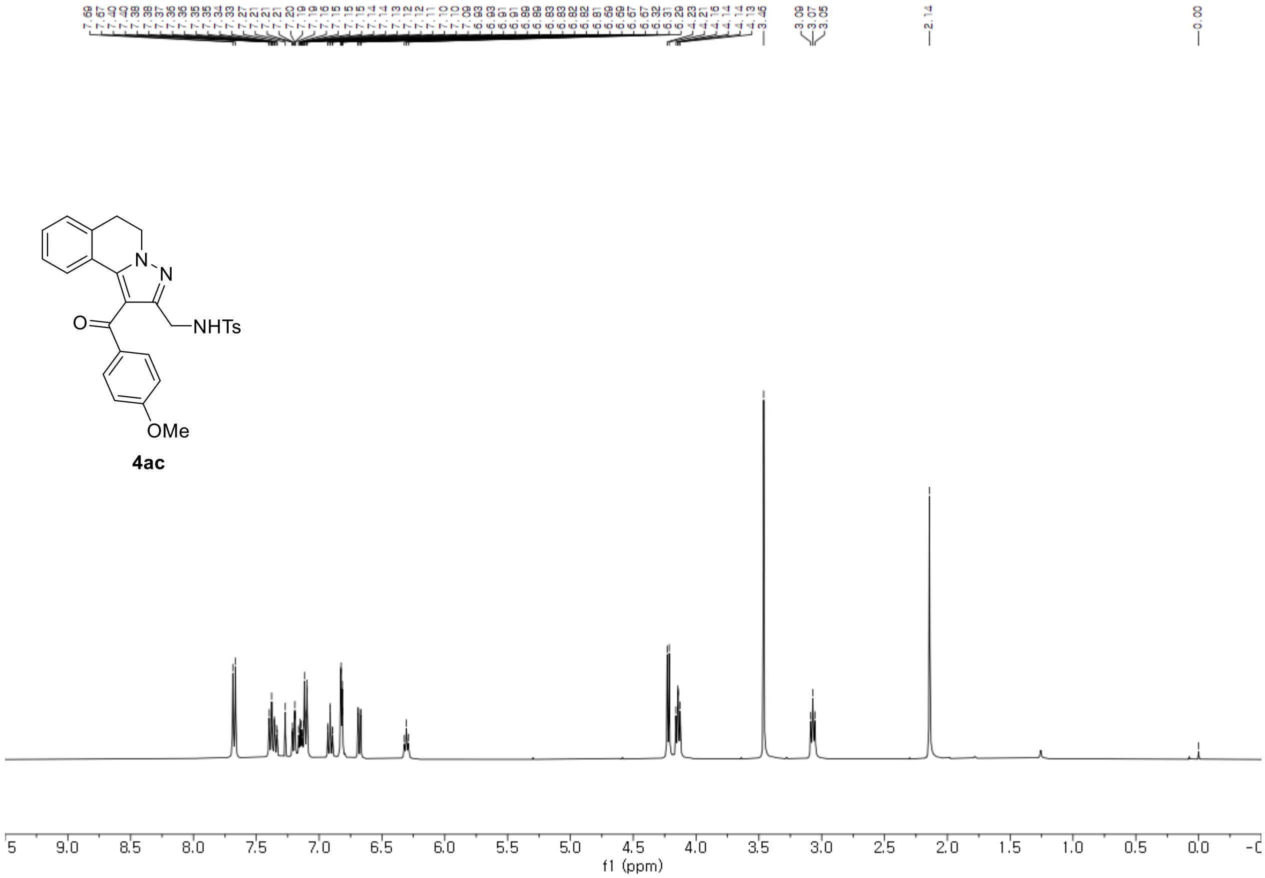

^{13}C NMR (100 MHz) in $CDCl_3$

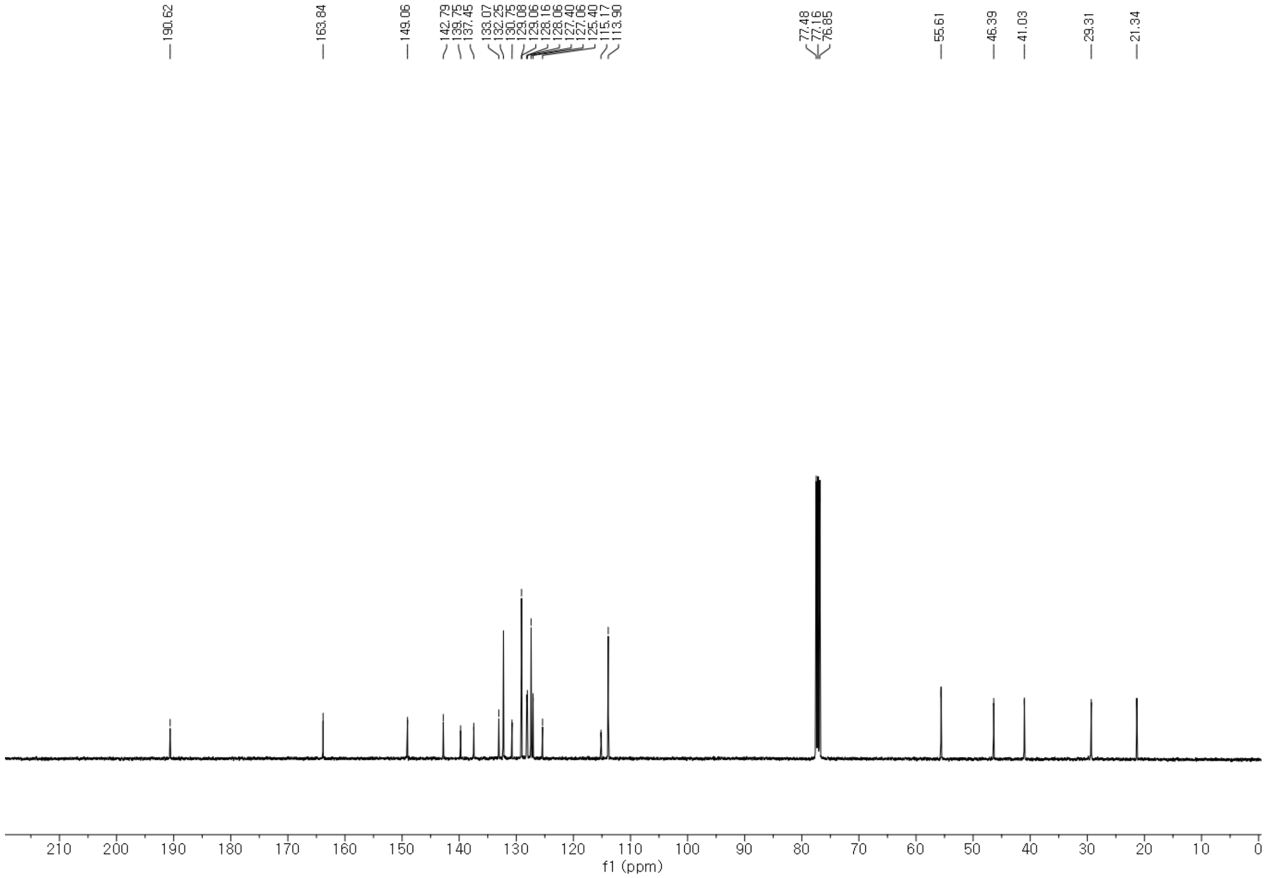

^{1}H NMR (400 MHz) in $CDCl_3$

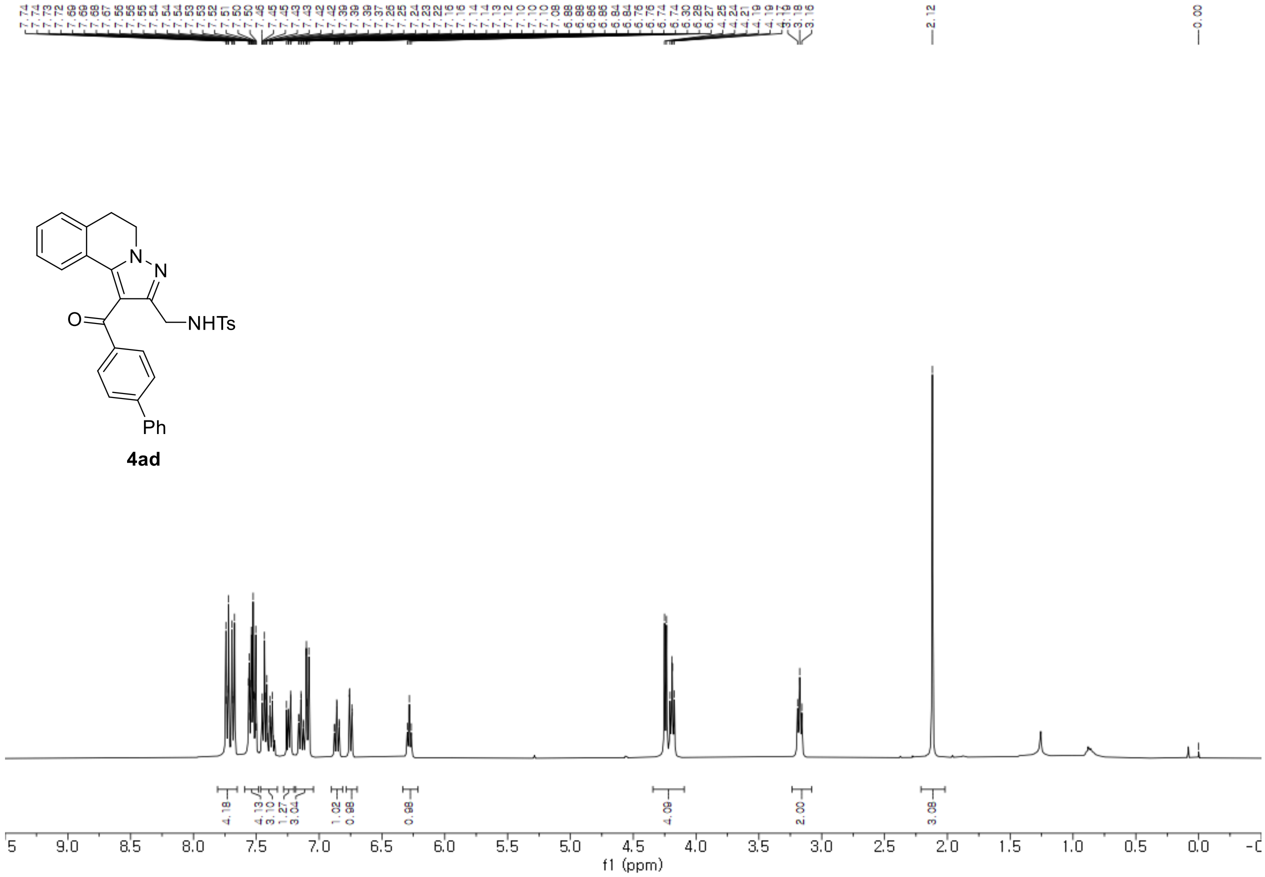

4ad

^{13}C NMR (100 MHz) in $CDCl_3$

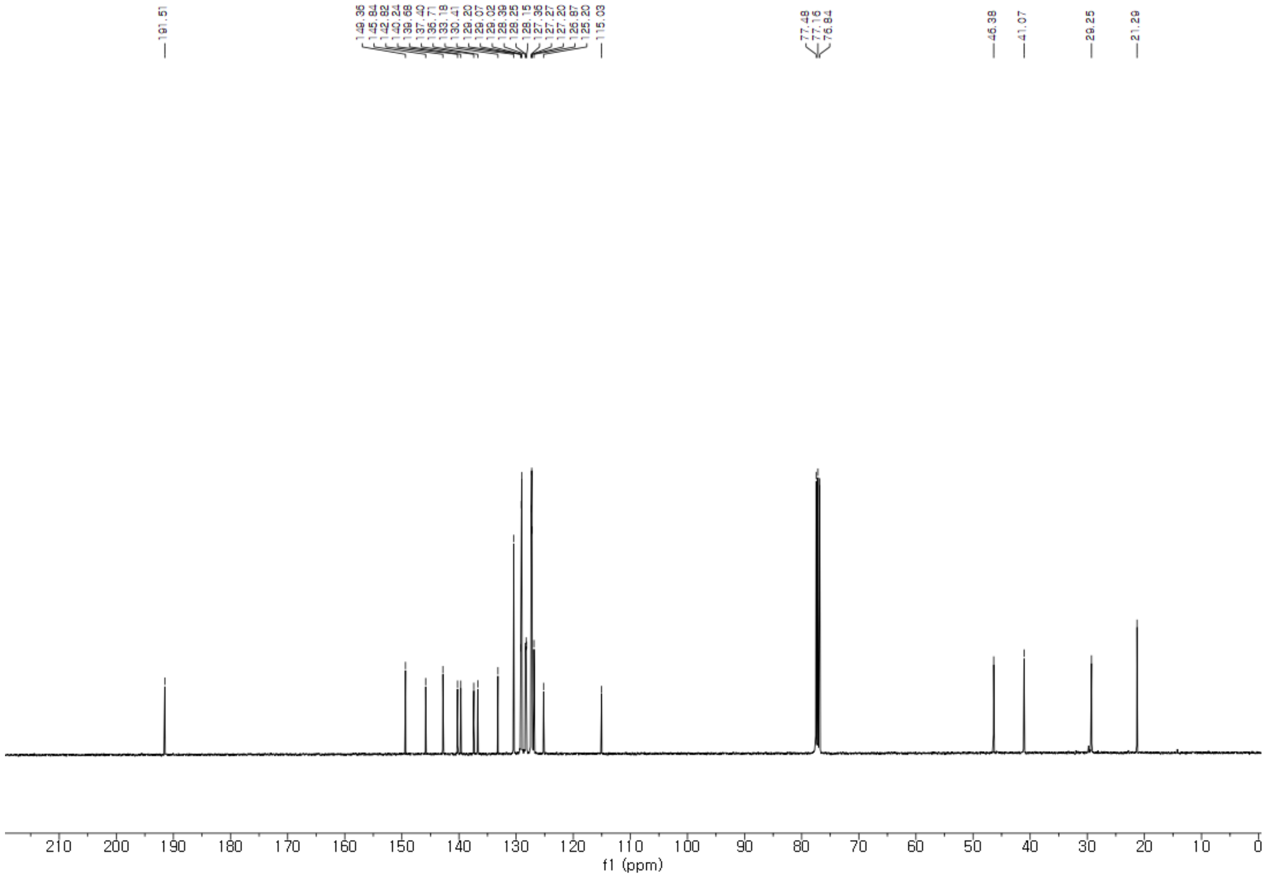

^{1}H NMR (400 MHz) in $CDCl_3$

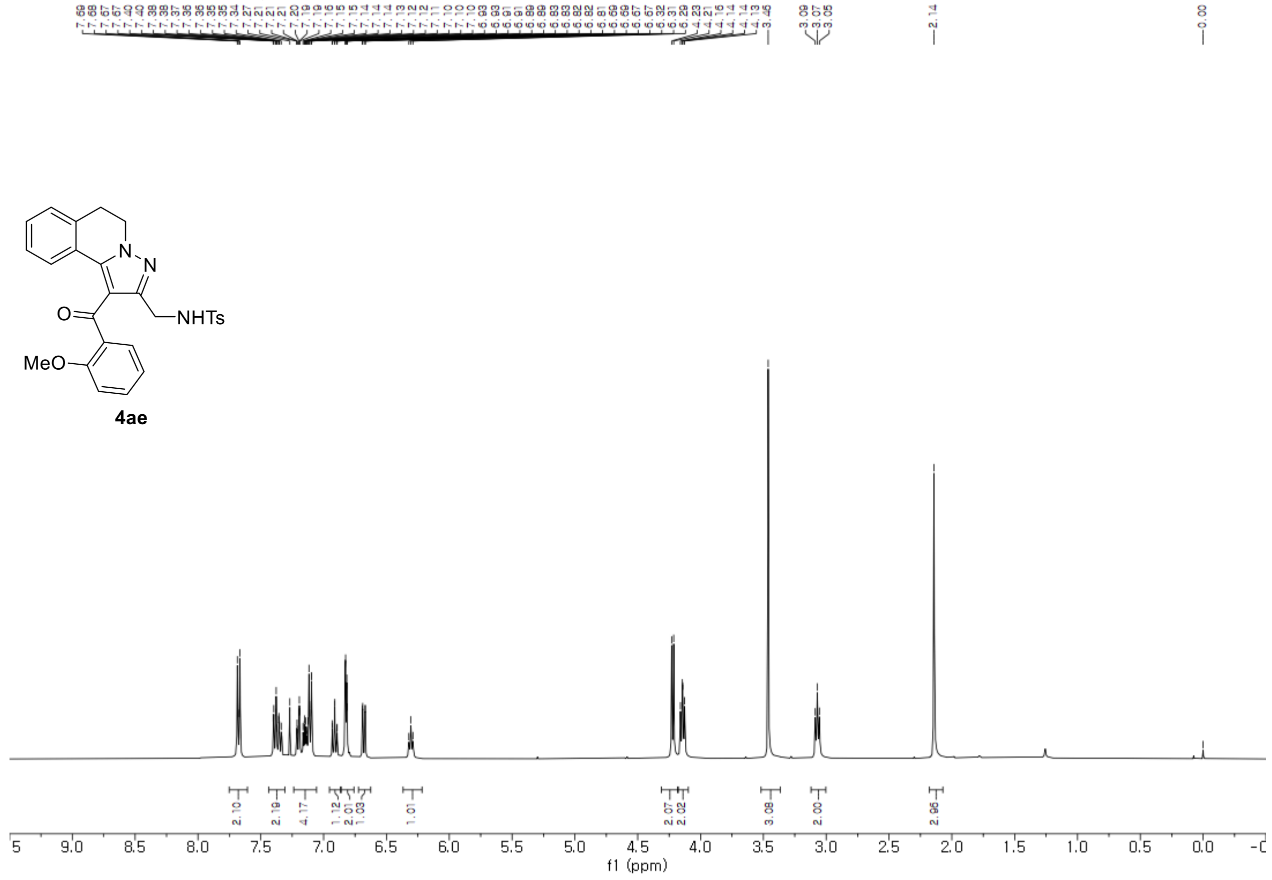

^{13}C NMR (100 MHz) in $CDCl_3$

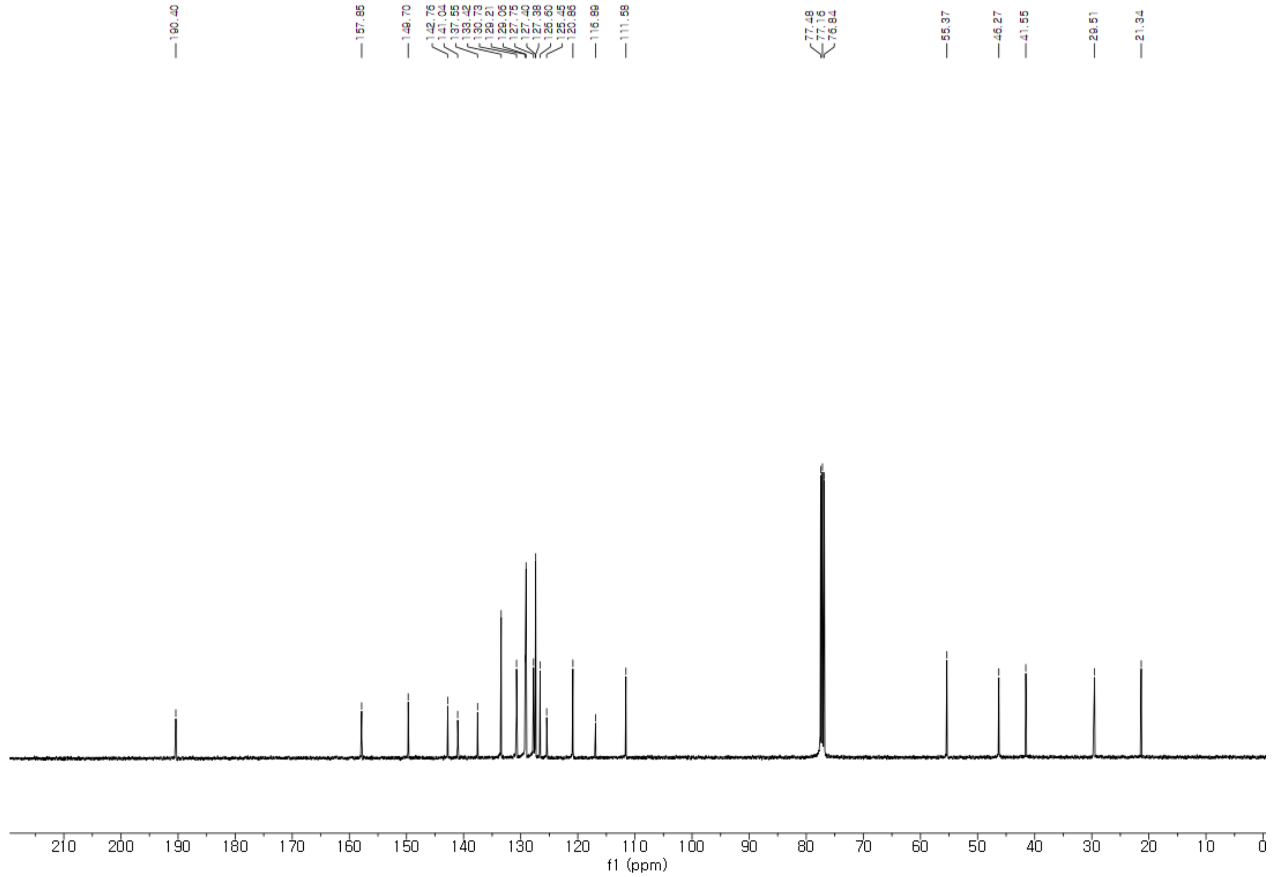

^{1}H NMR (400 MHz) in $CDCl_3$

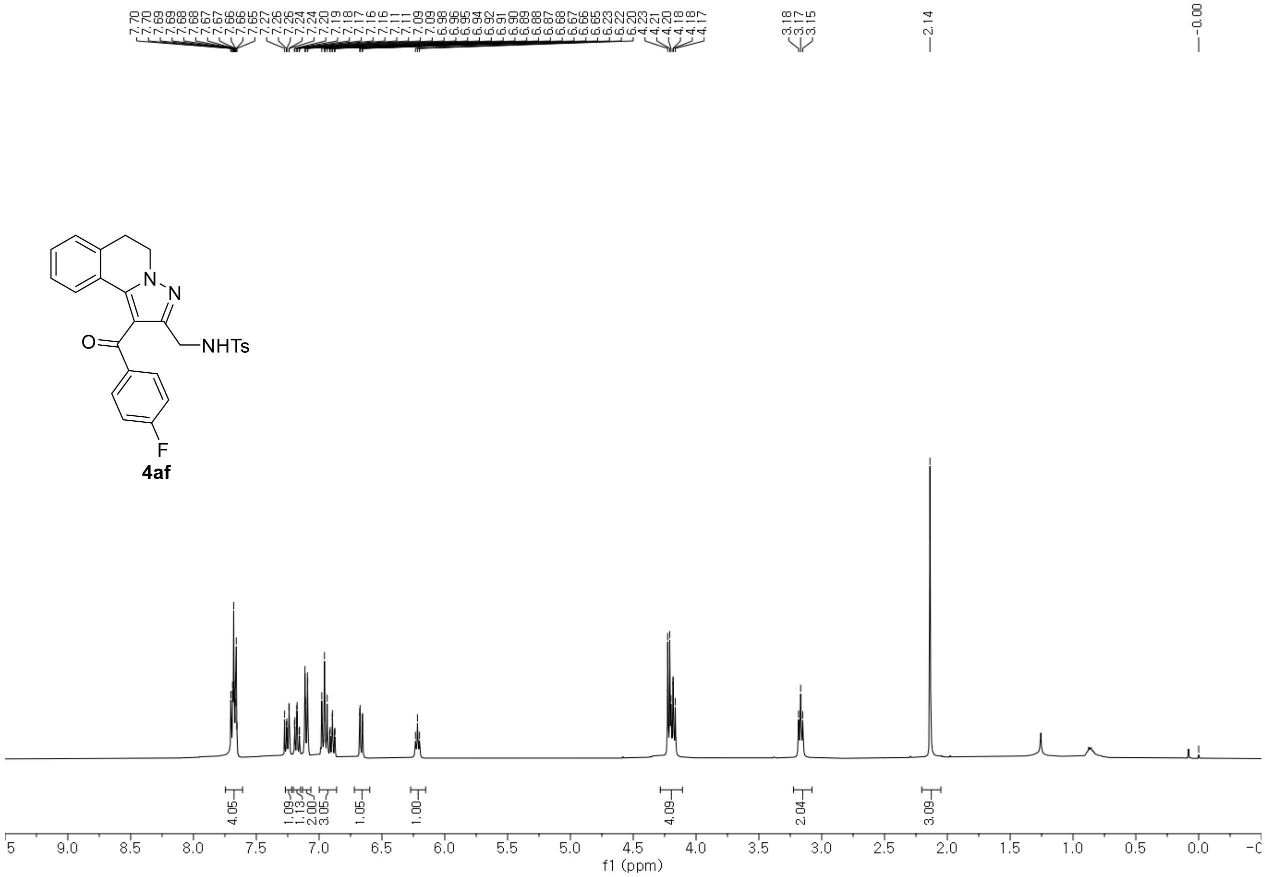

^{13}C NMR (100 MHz) in $CDCl_3$

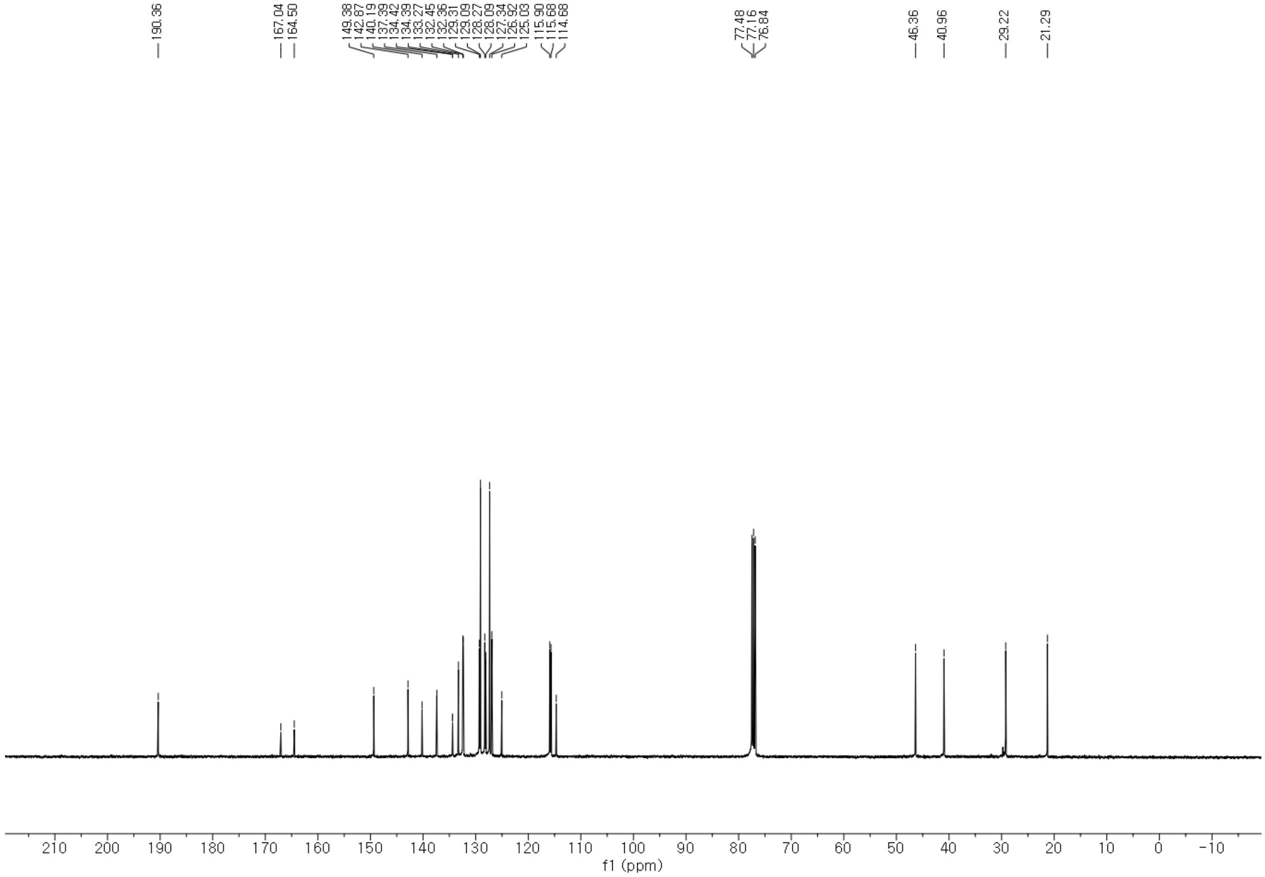

^{19}F NMR (376 MHz) in $CDCl_3$

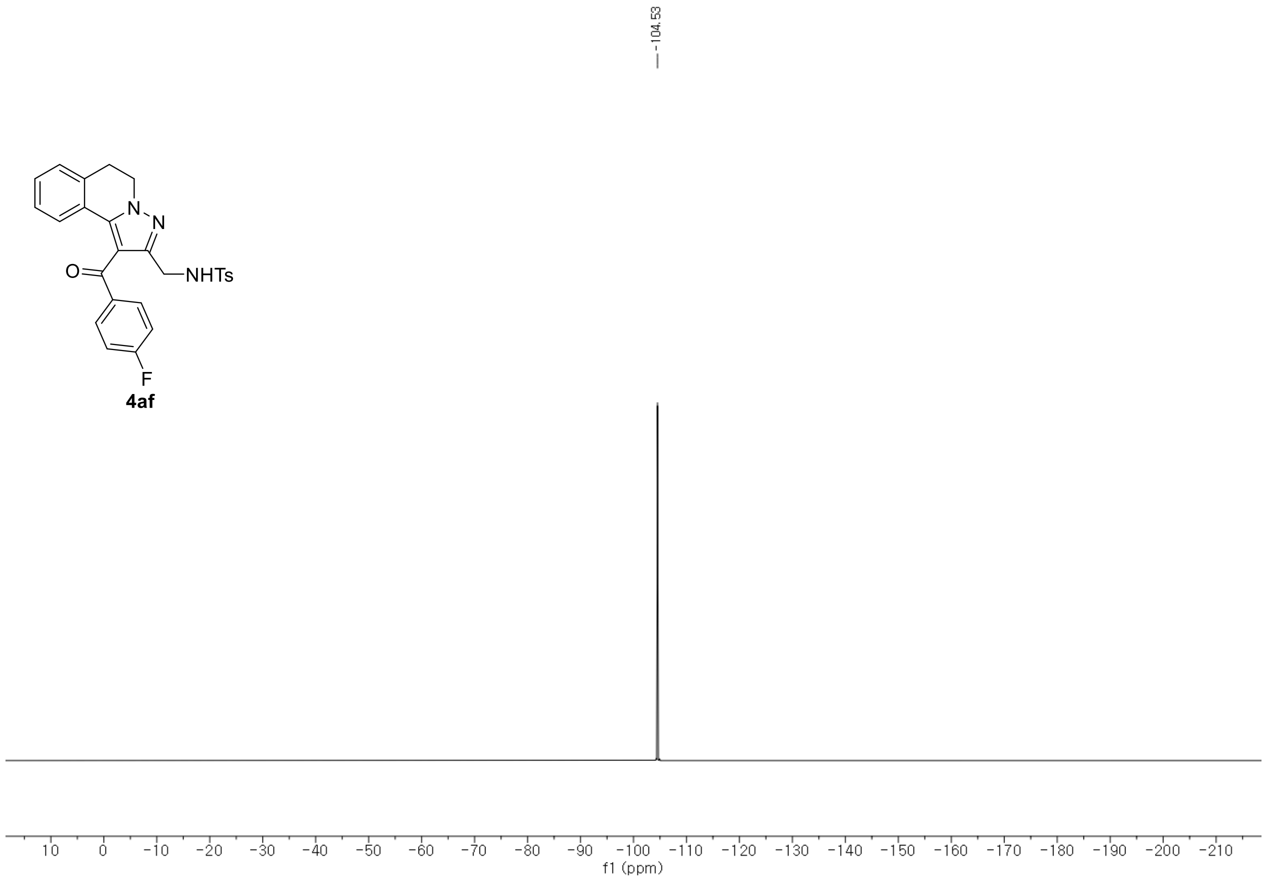

^{1}H NMR (400 MHz) in $CDCl_3$

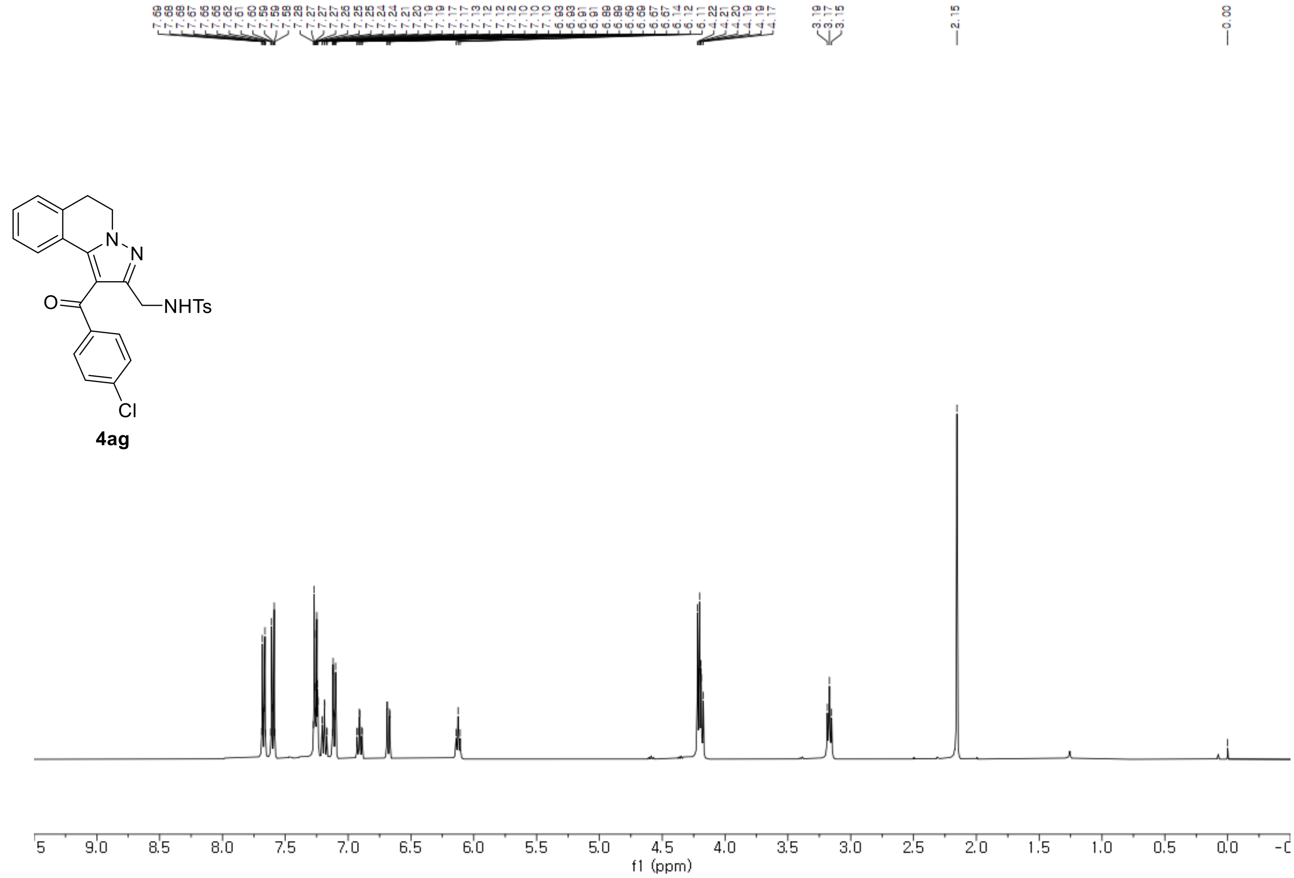

^{13}C NMR (100 MHz) in $CDCl_3$

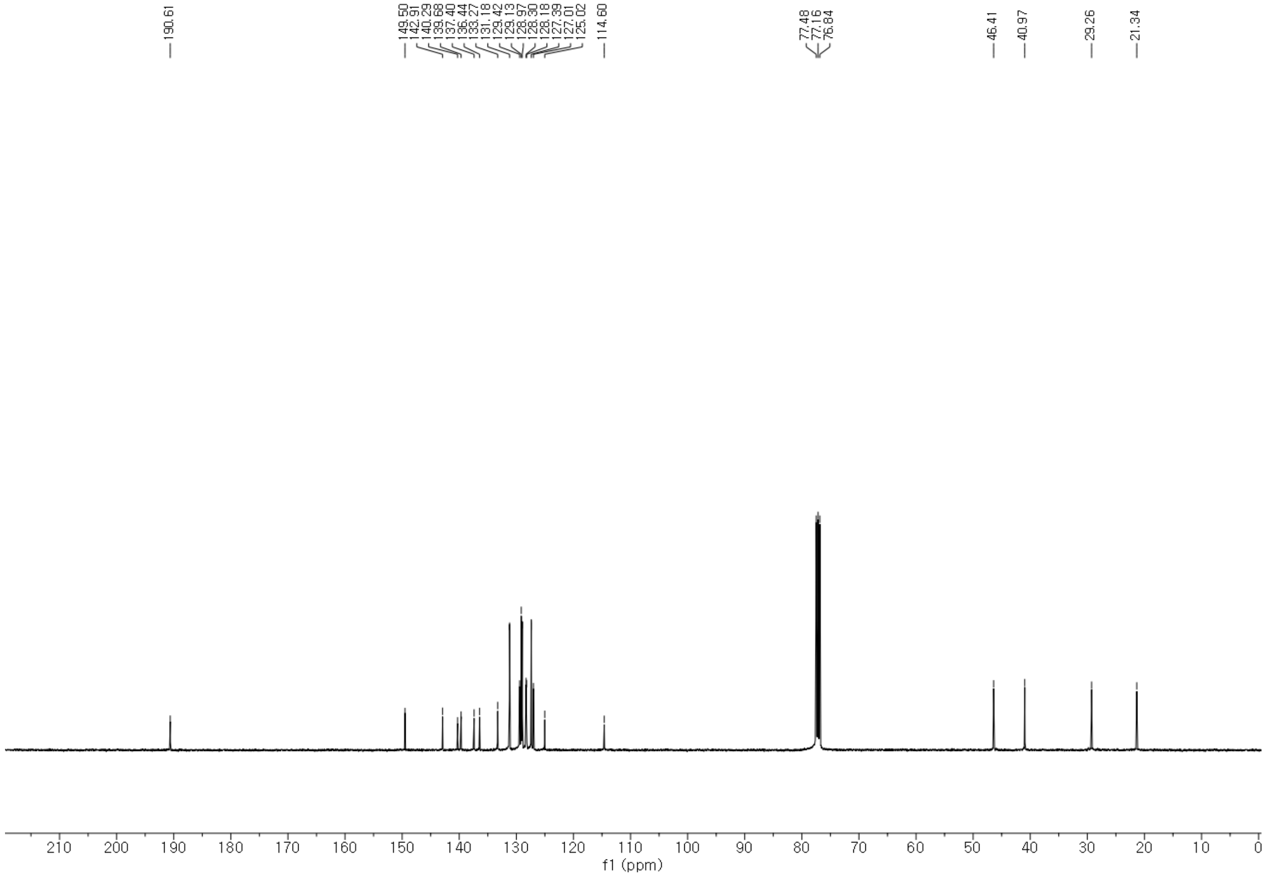

^{1}H NMR (400 MHz) in $CDCl_3$

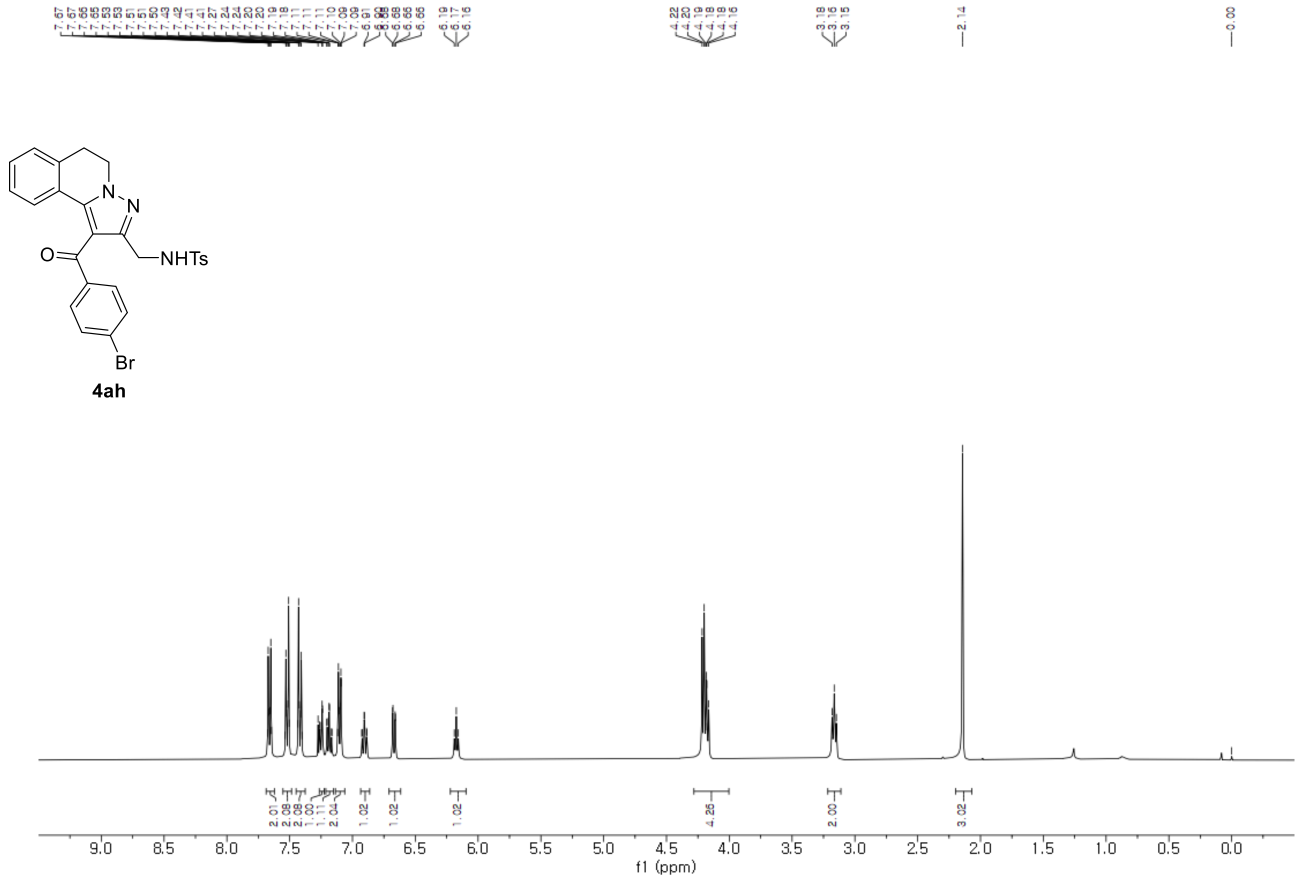

^{13}C NMR (100 MHz) in $CDCl_3$

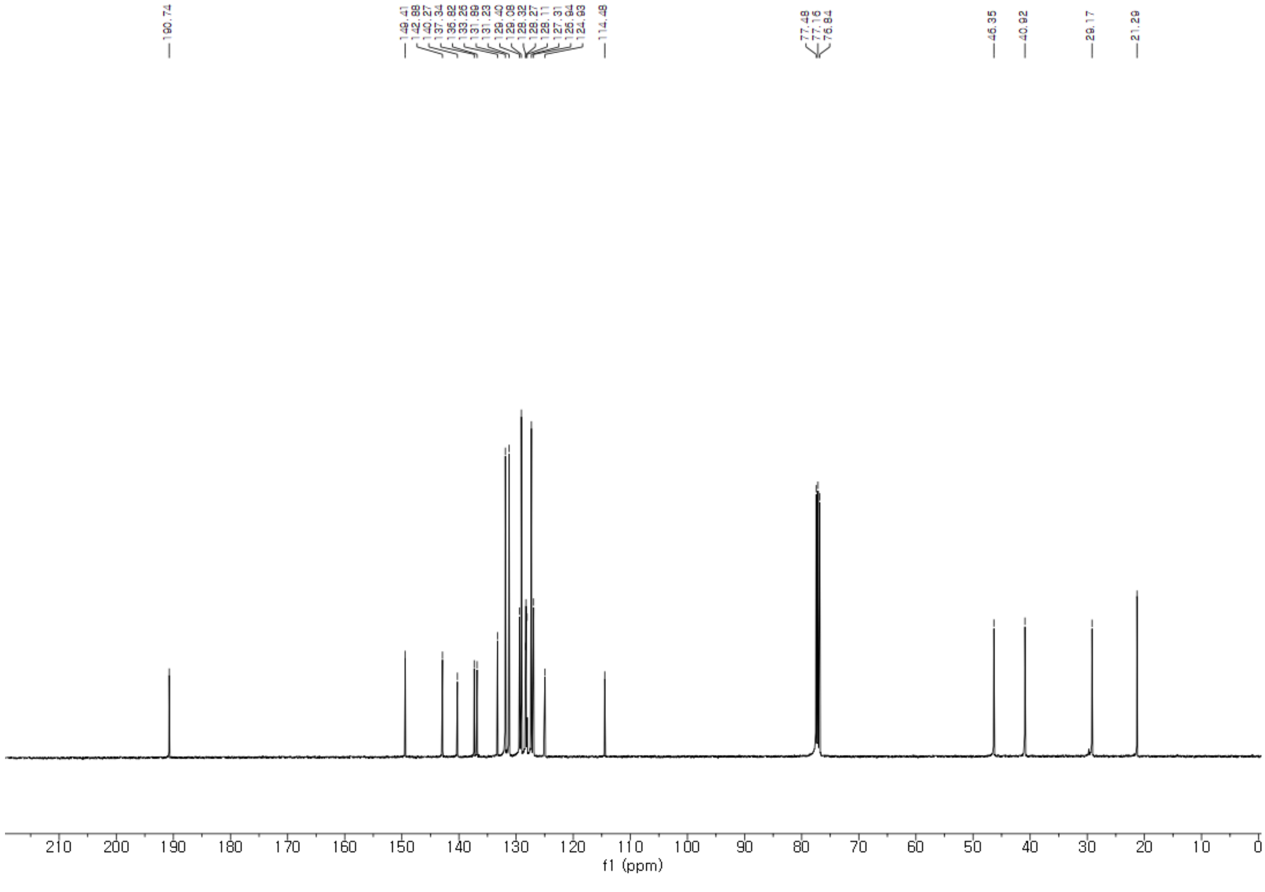

^{1}H NMR (400 MHz) in $CDCl_3$

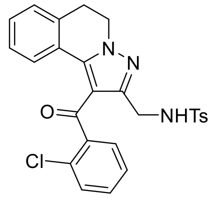

4ai

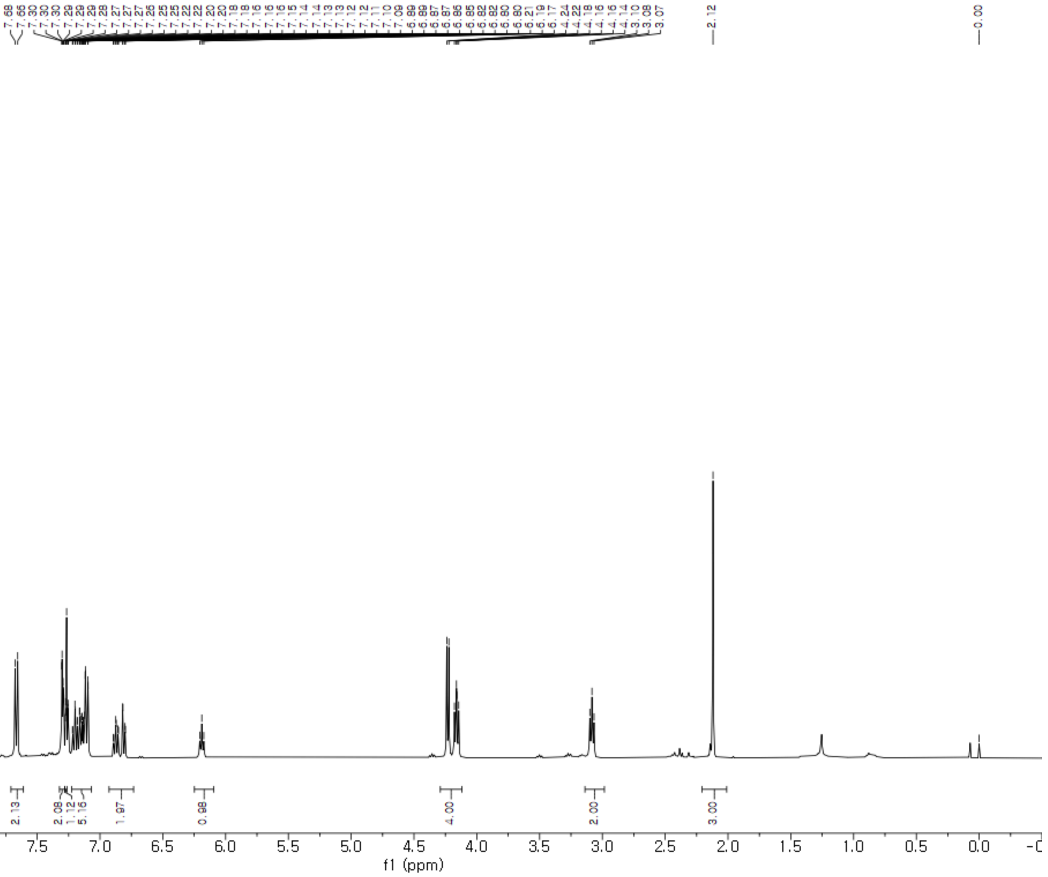

^{13}C NMR (100 MHz) in $CDCl_3$

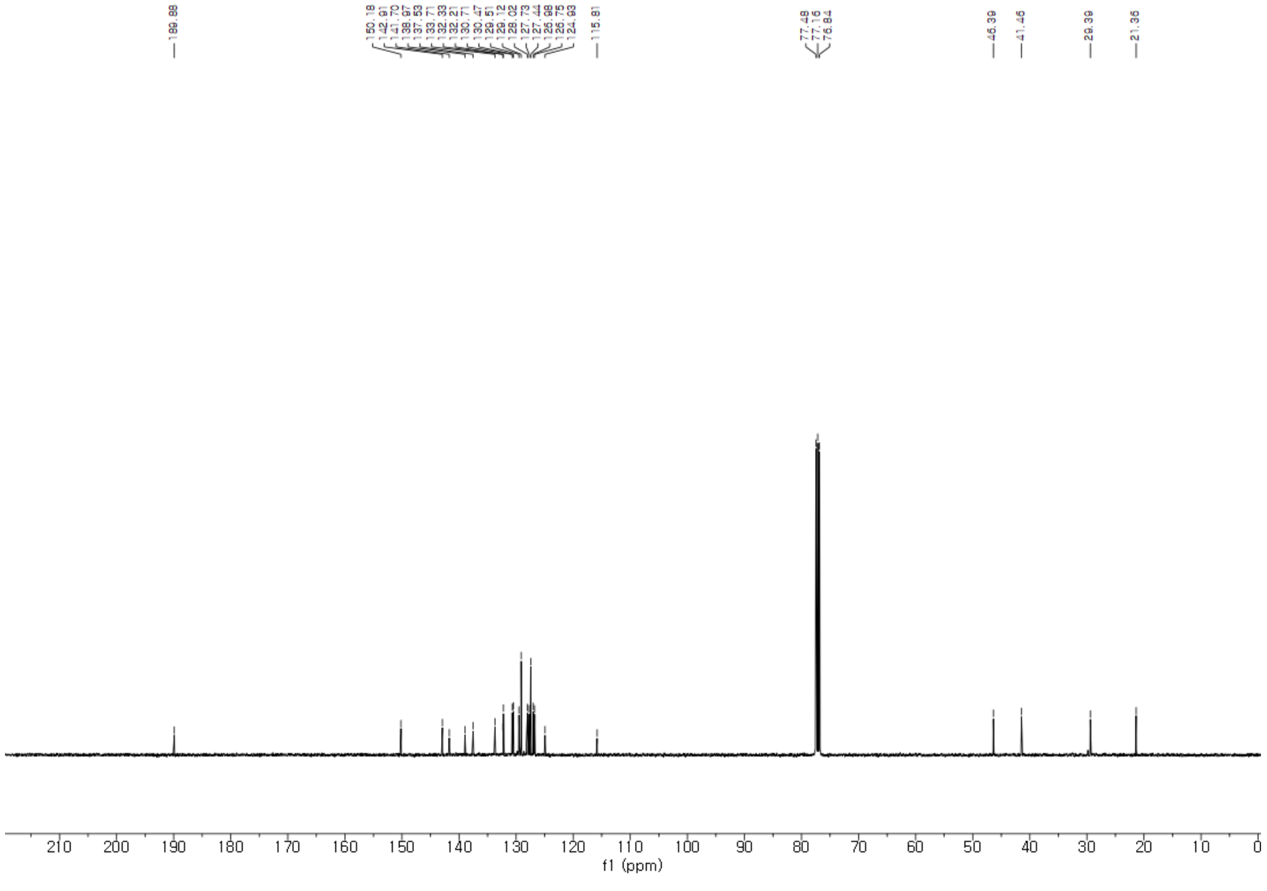

^{1}H NMR (400 MHz) in $CDCl_3$

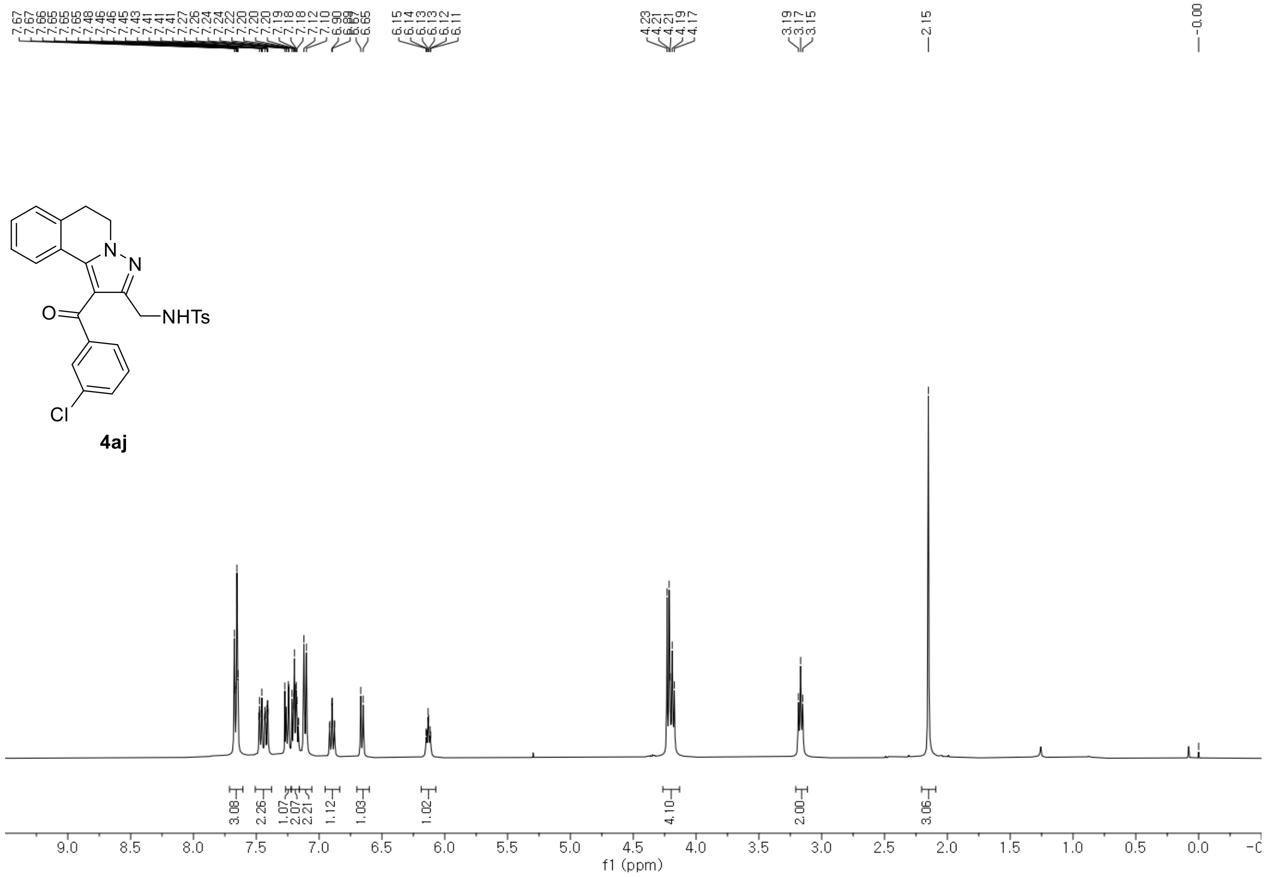

^{13}C NMR (100 MHz) in $CDCl_3$

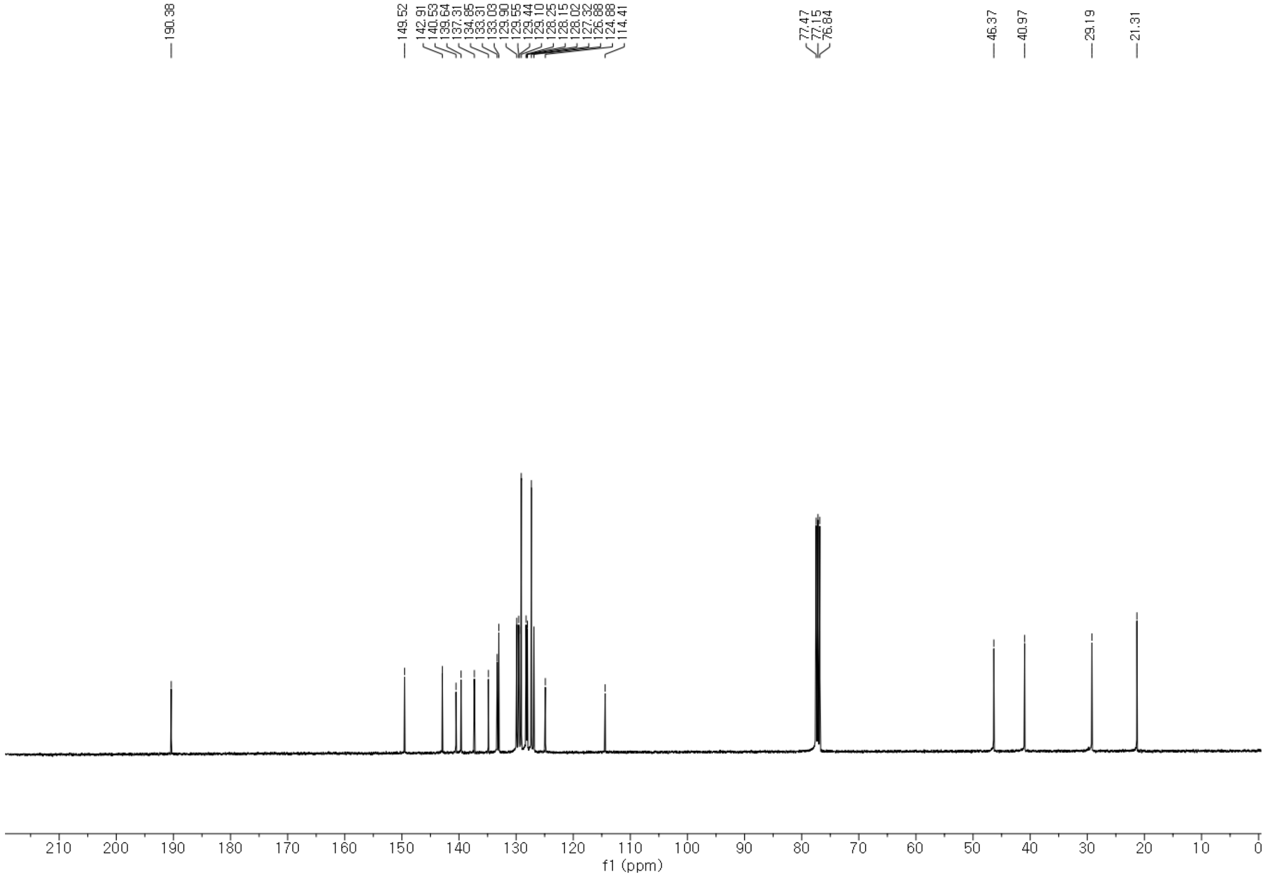

^{1}H NMR (400 MHz) in $CDCl_3$

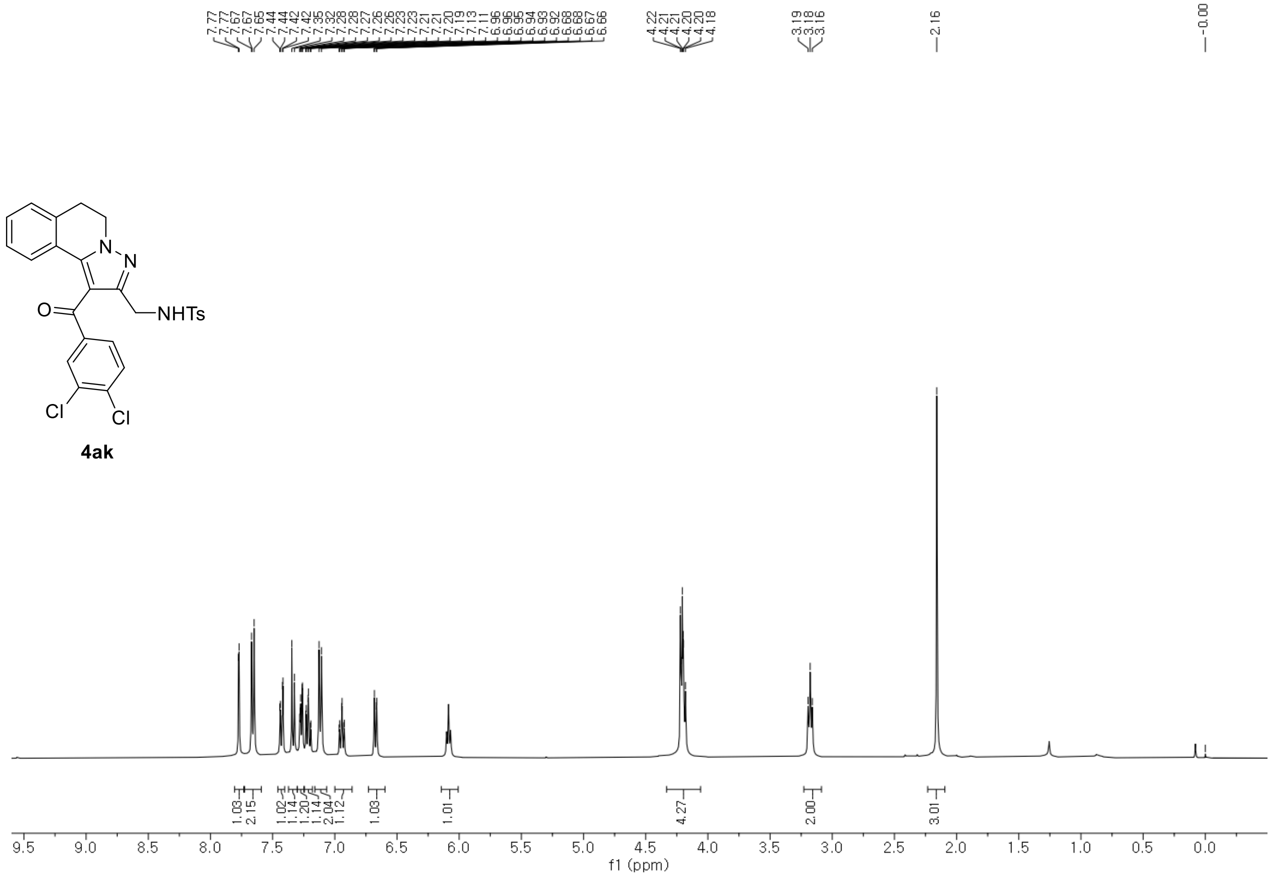

^{13}C NMR (100 MHz) in $CDCl_3$

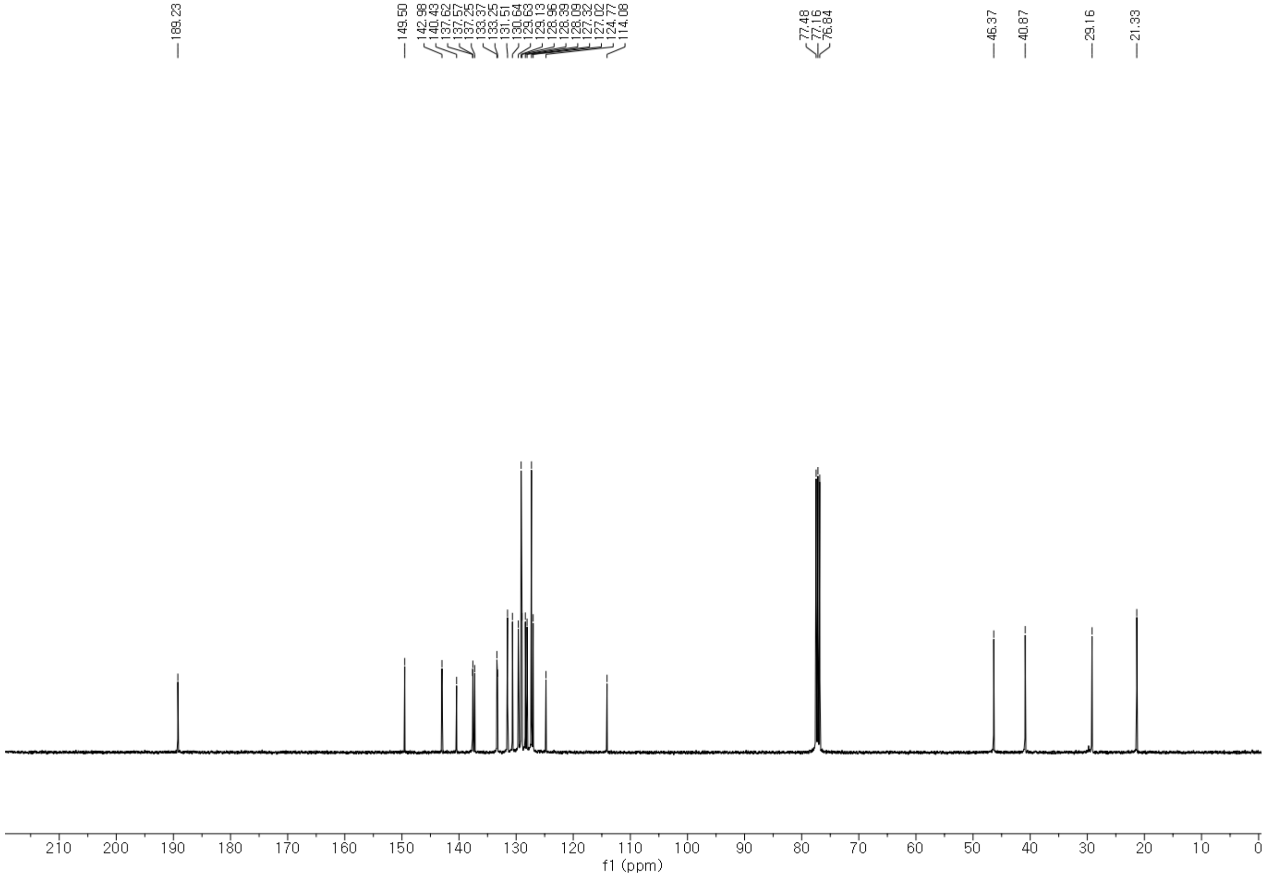

^{1}H NMR (400 MHz) in $CDCl_3$

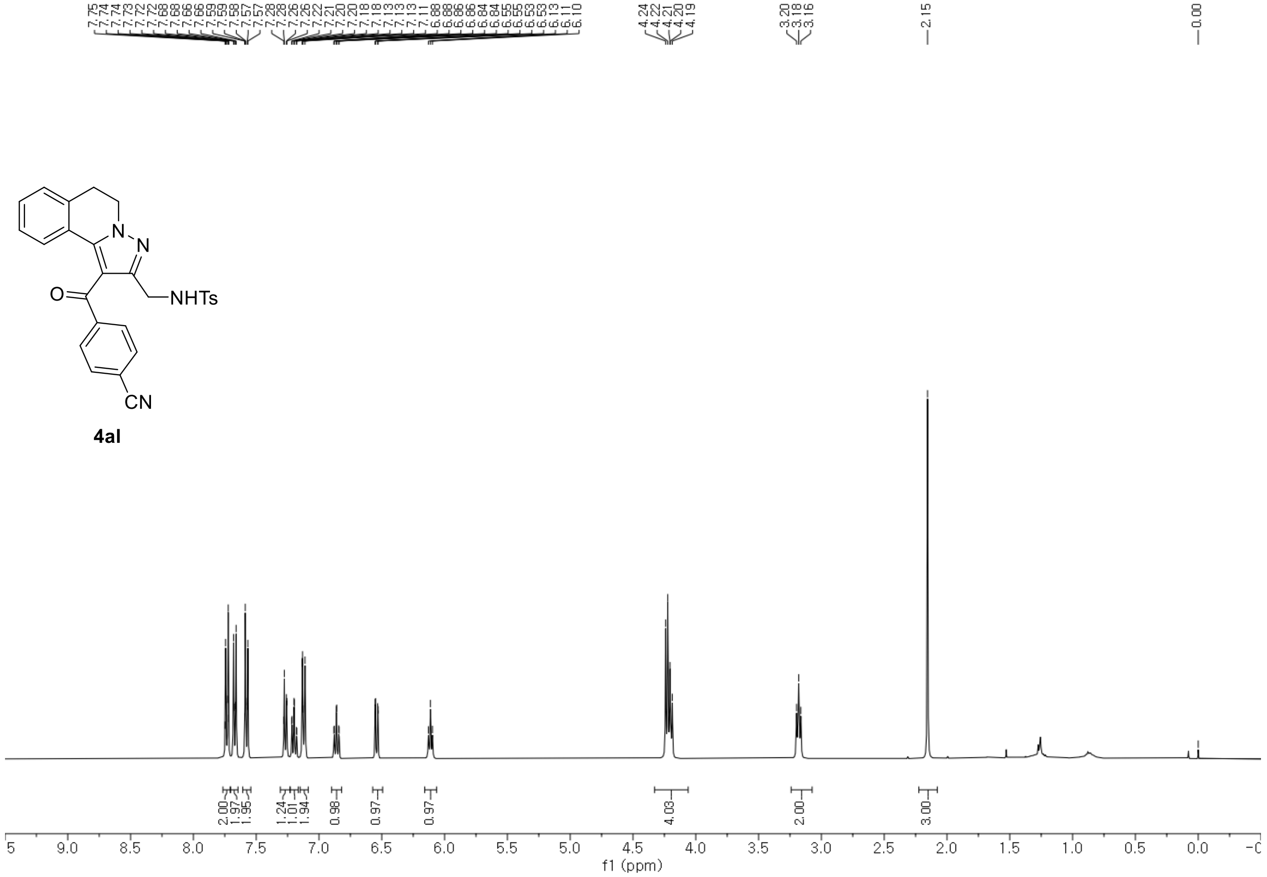

^{13}C NMR (100 MHz) in $CDCl_3$

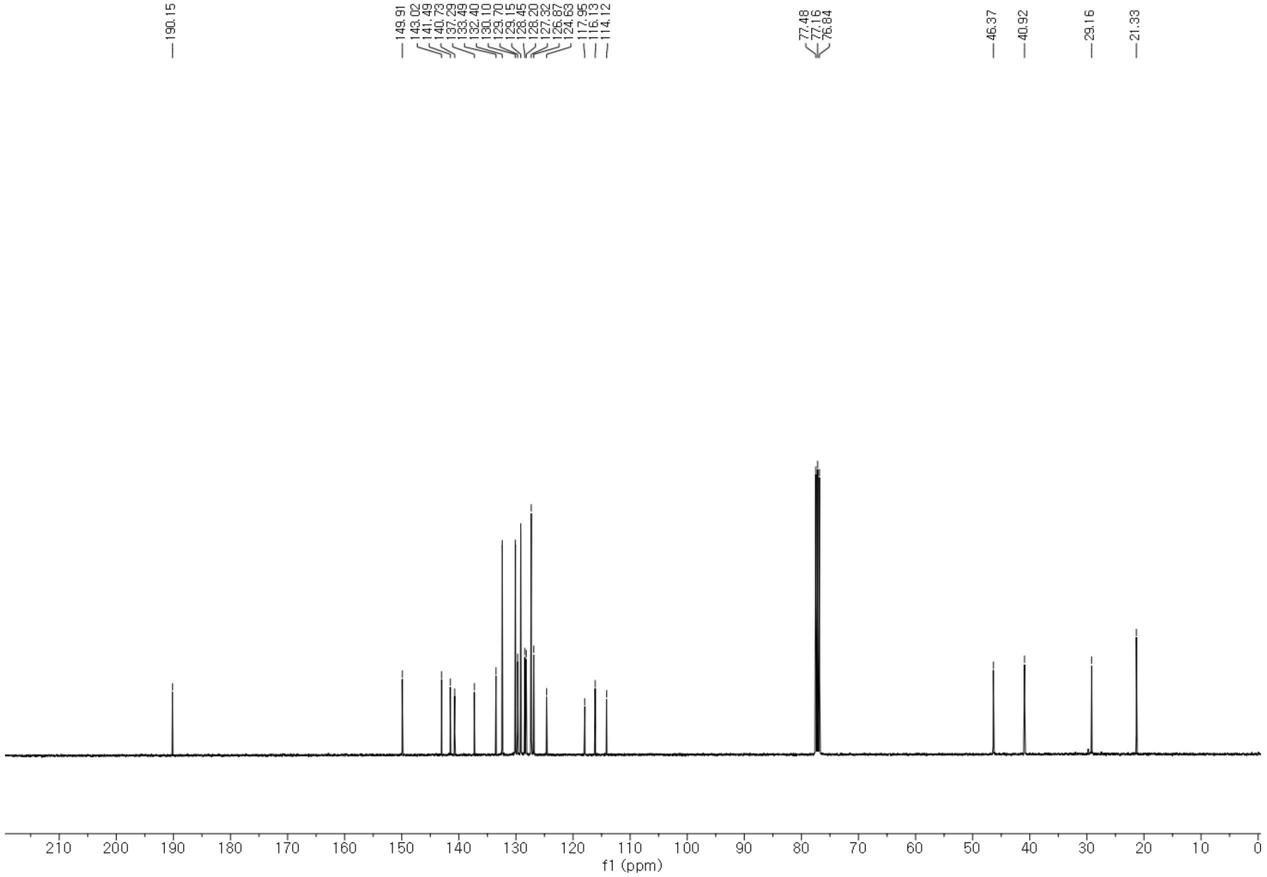

^{1}H NMR (400 MHz) in $CDCl_3$

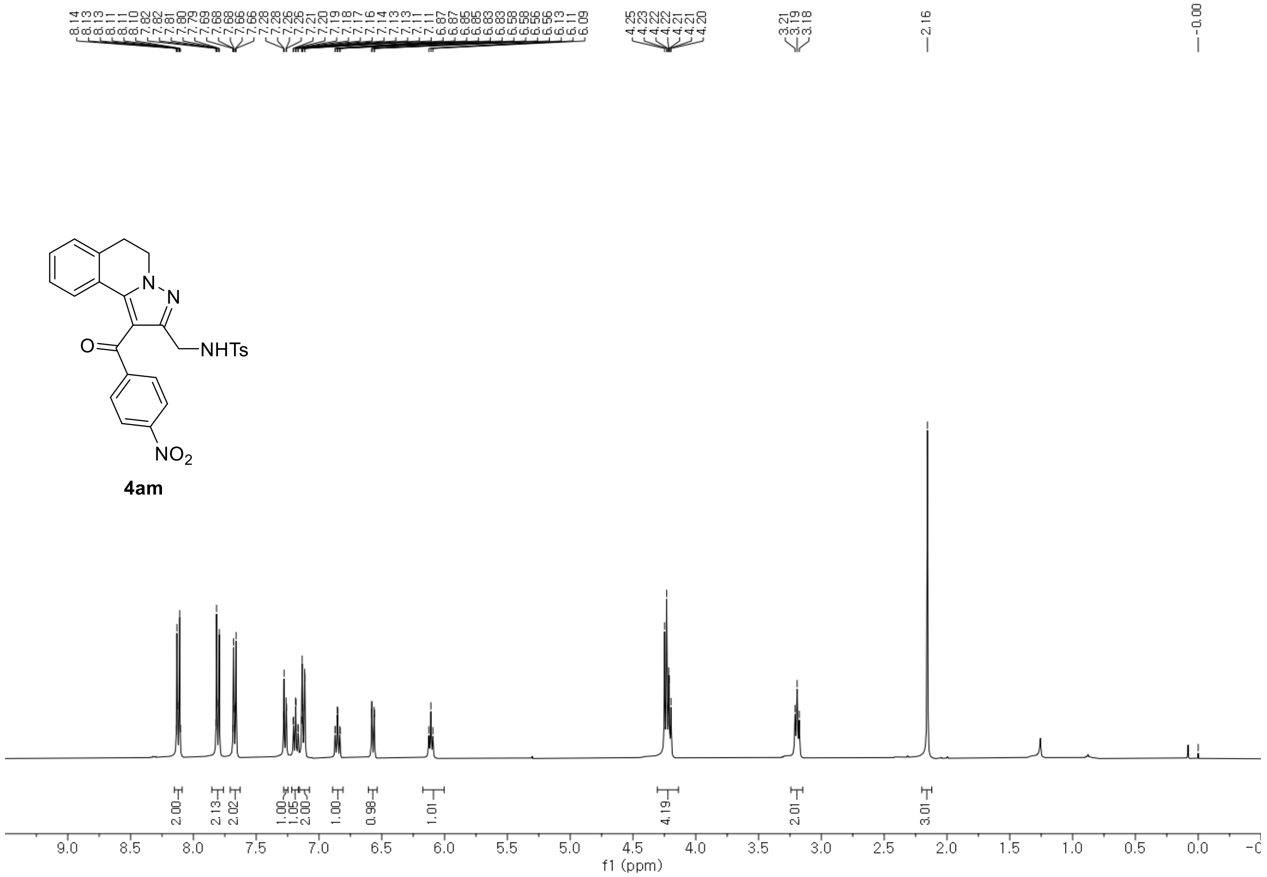

^{13}C NMR (100 MHz) in $CDCl_3$

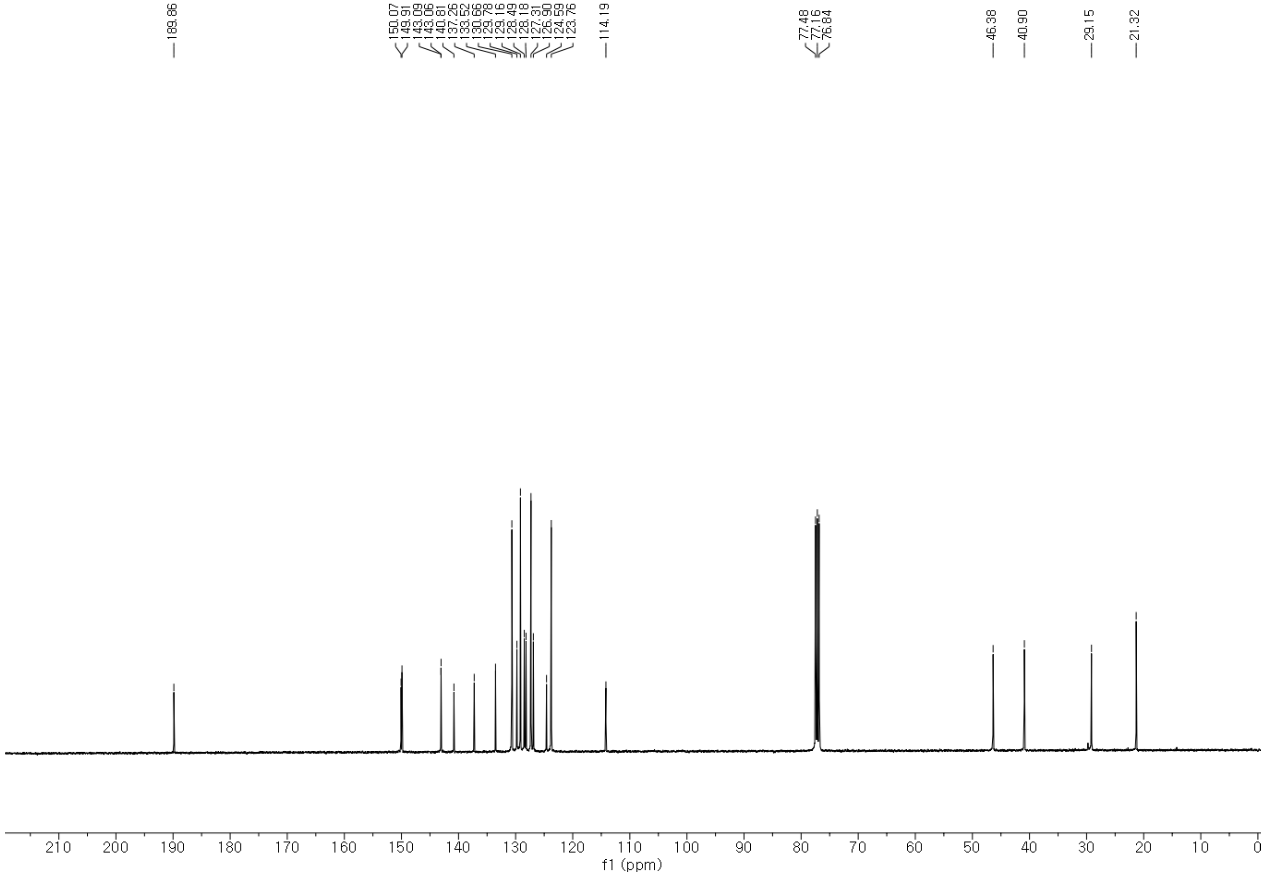

^{1}H NMR (400 MHz) in $CDCl_3$

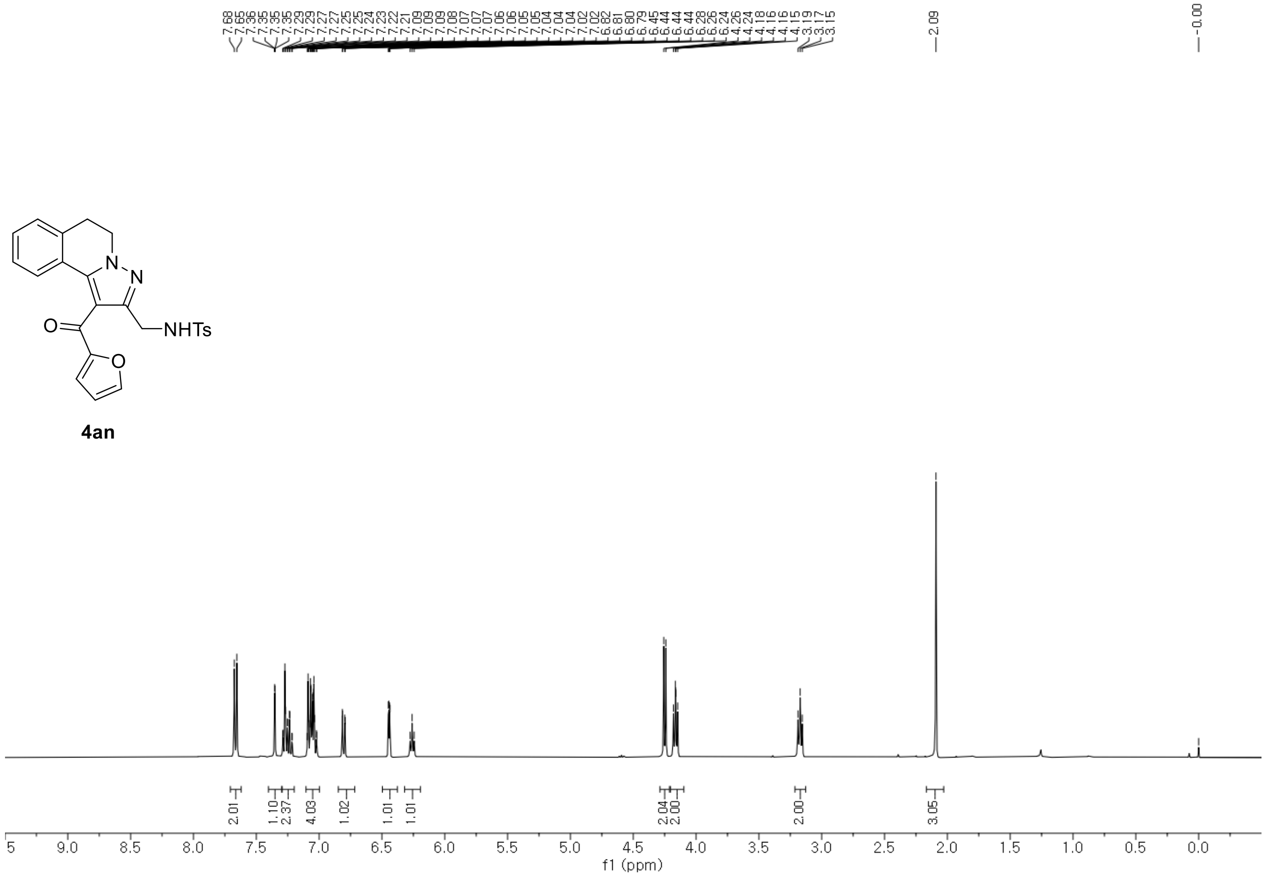

4an

^{13}C NMR (100 MHz) in $CDCl_3$

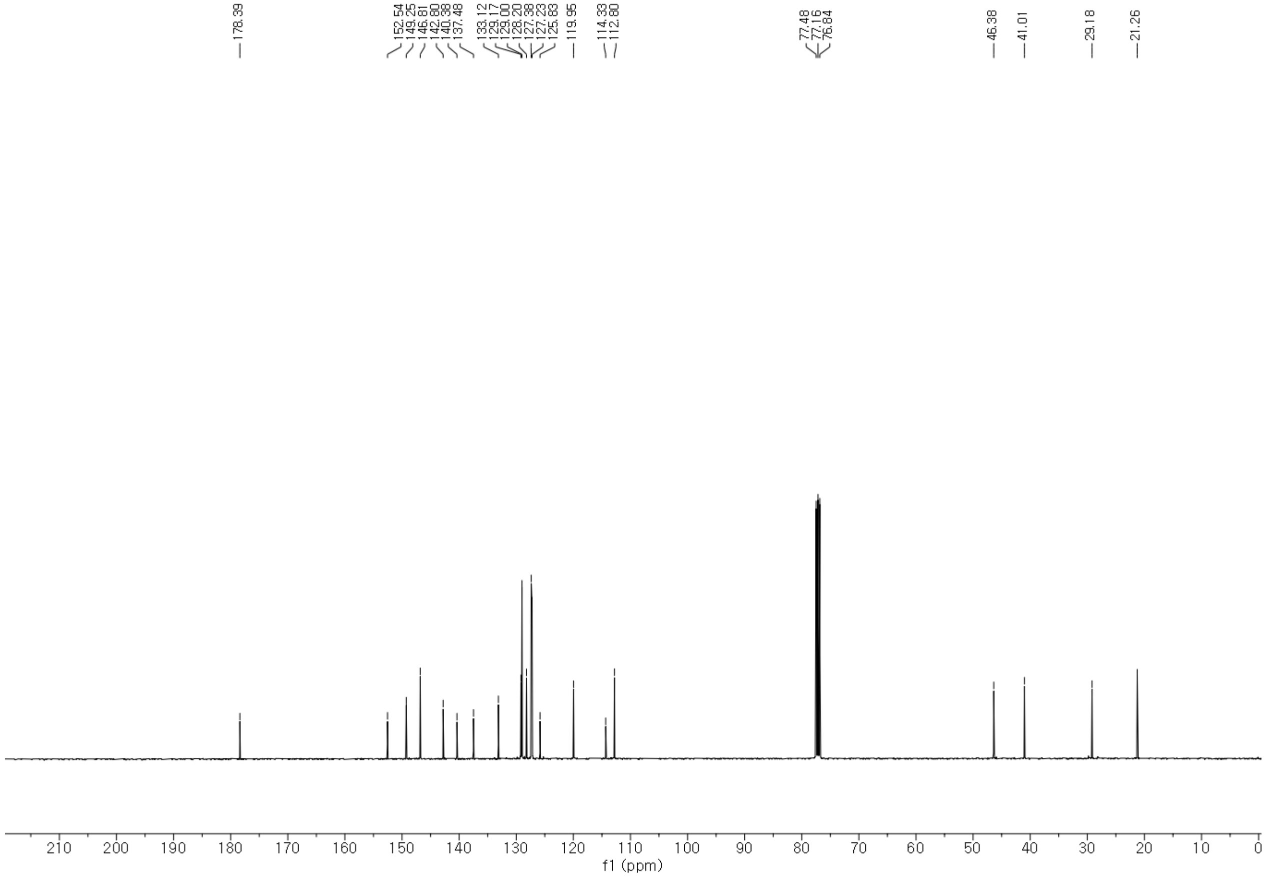

^{1}H NMR (400 MHz) in $CDCl_3$

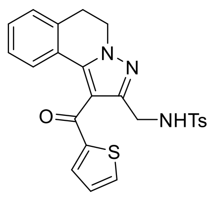

4ao

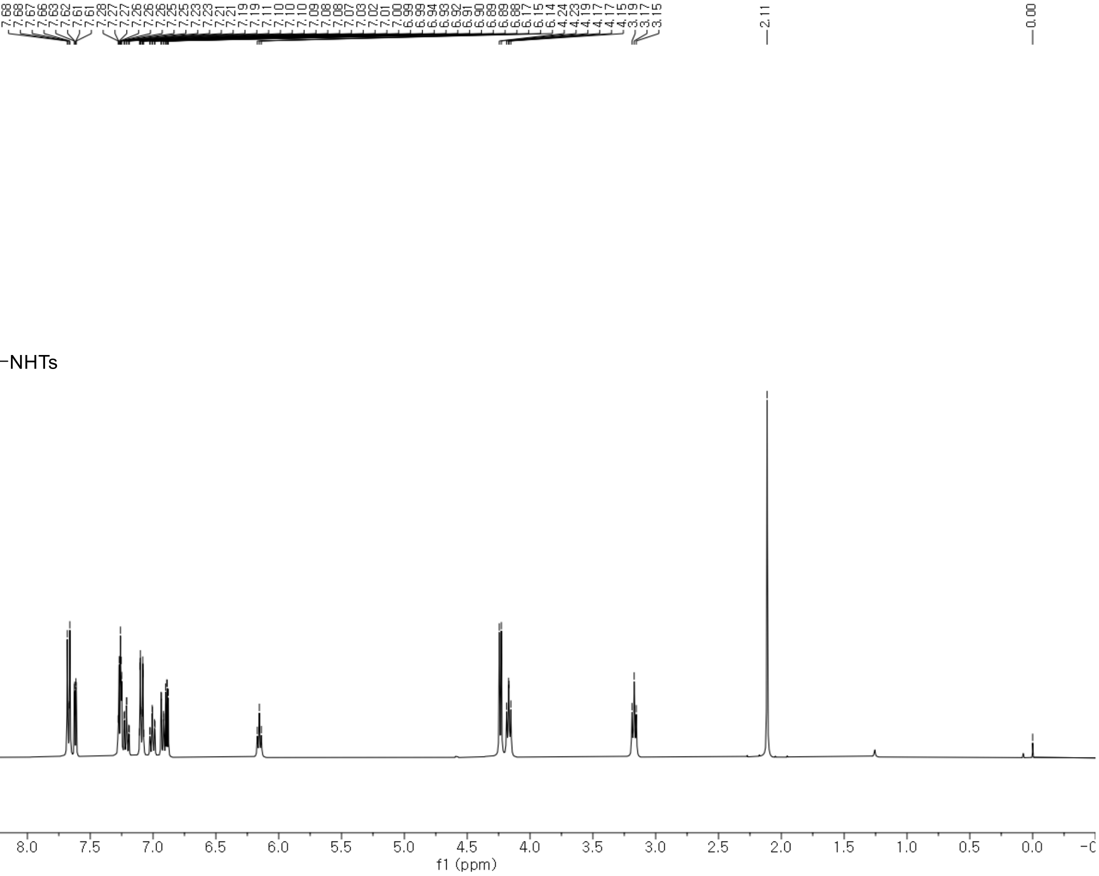

^{13}C NMR (100 MHz) in $CDCl_3$

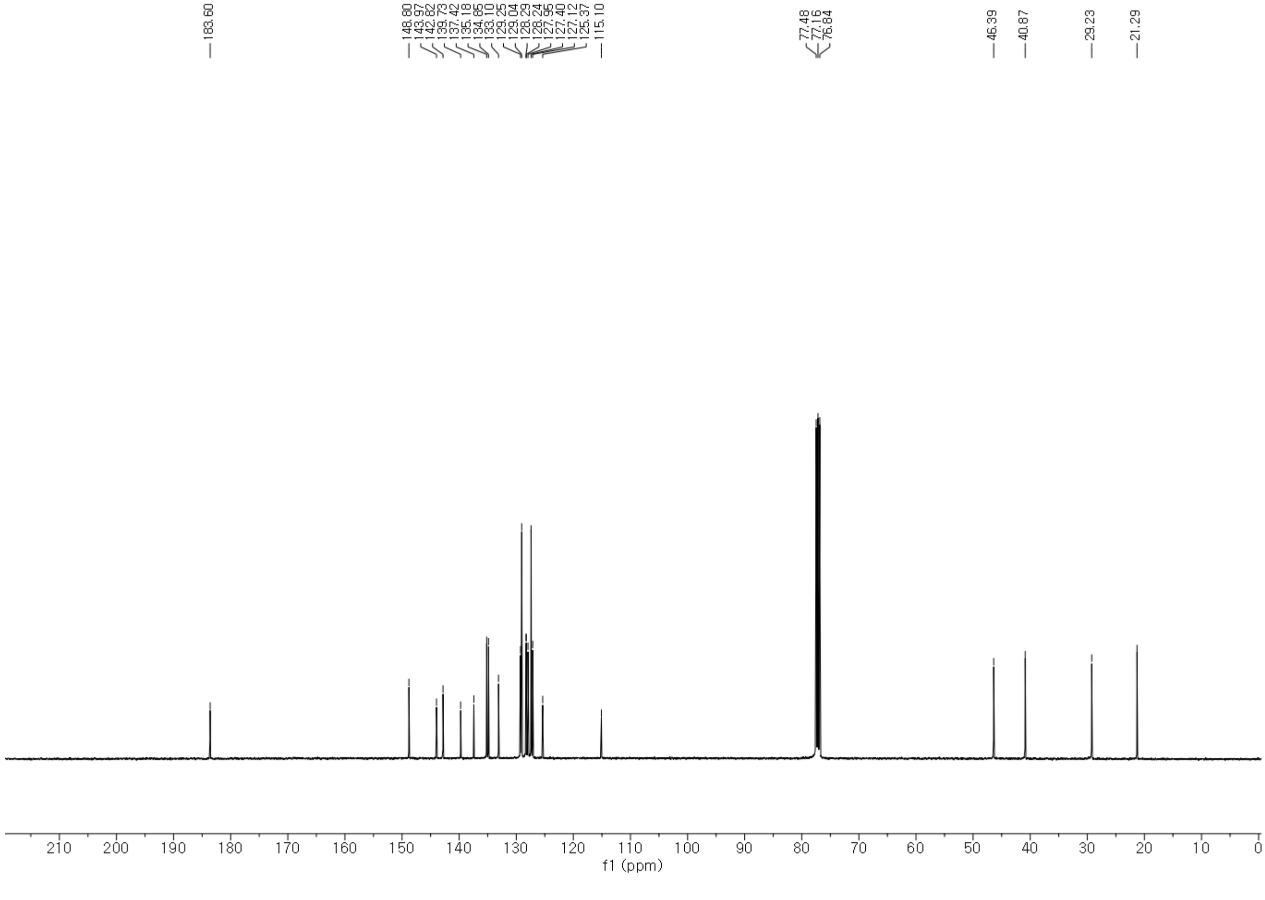

^{1}H NMR (400 MHz) in $CDCl_3$

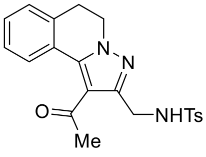

4ap

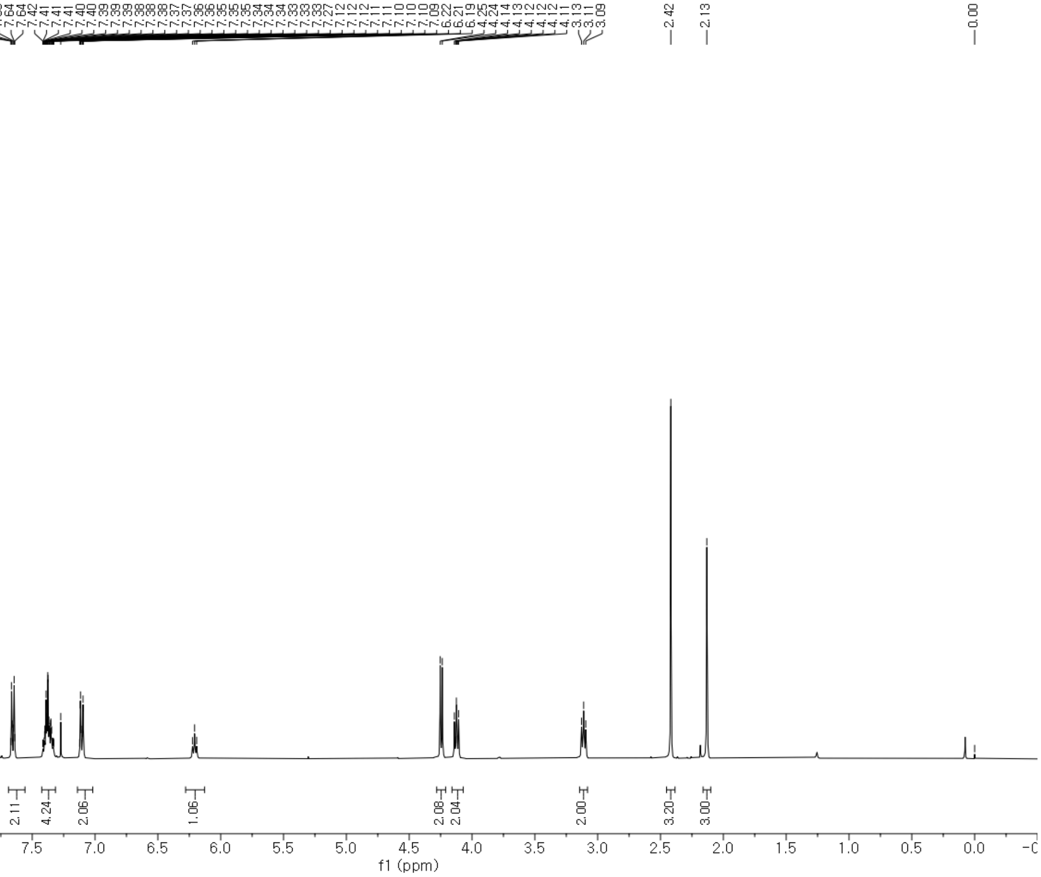

^{13}C NMR (100 MHz) in $CDCl_3$

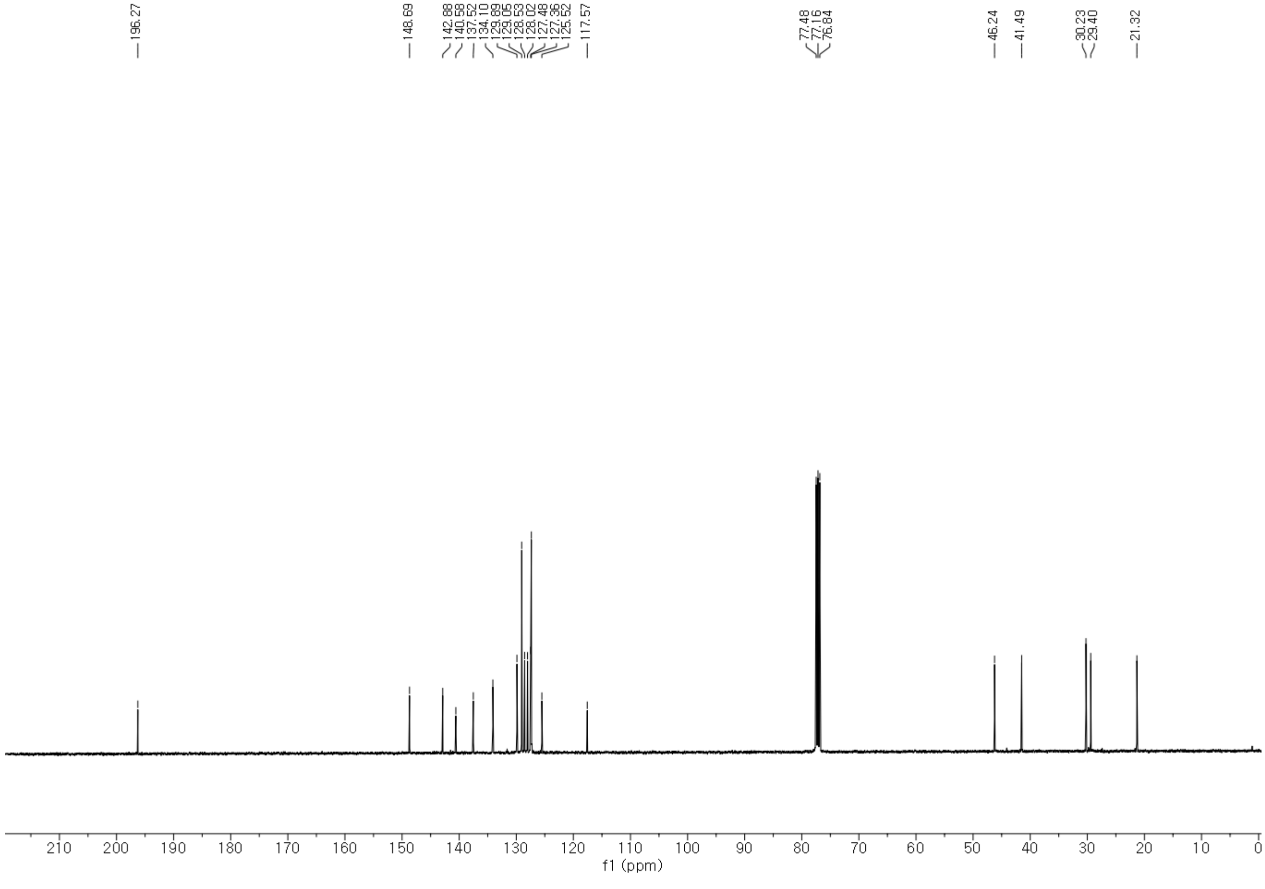

^{1}H NMR (400 MHz) in $CDCl_3$

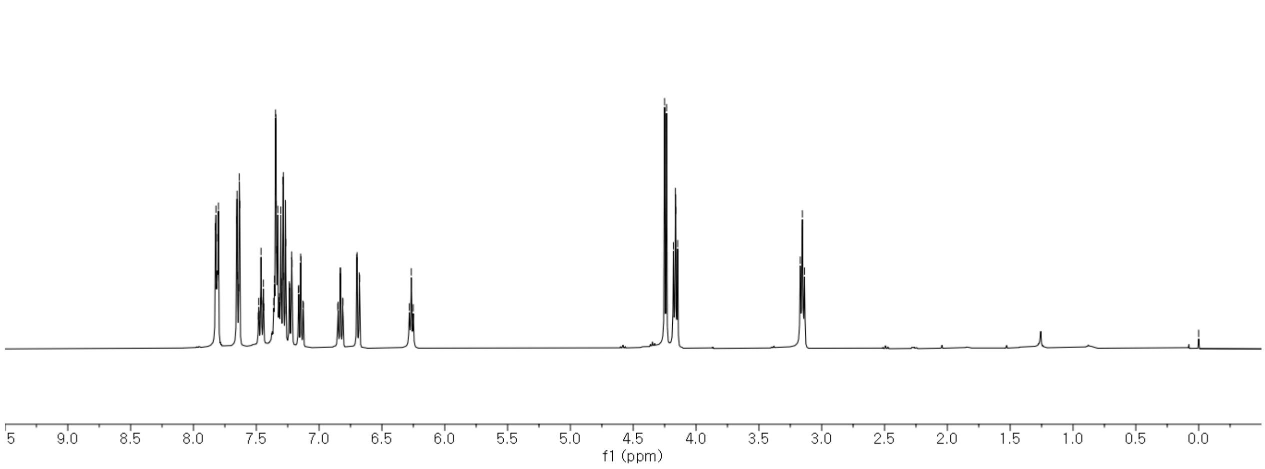

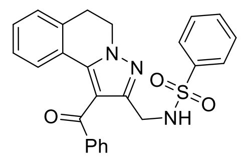

4aq

^{13}C NMR (100 MHz) in $CDCl_3$

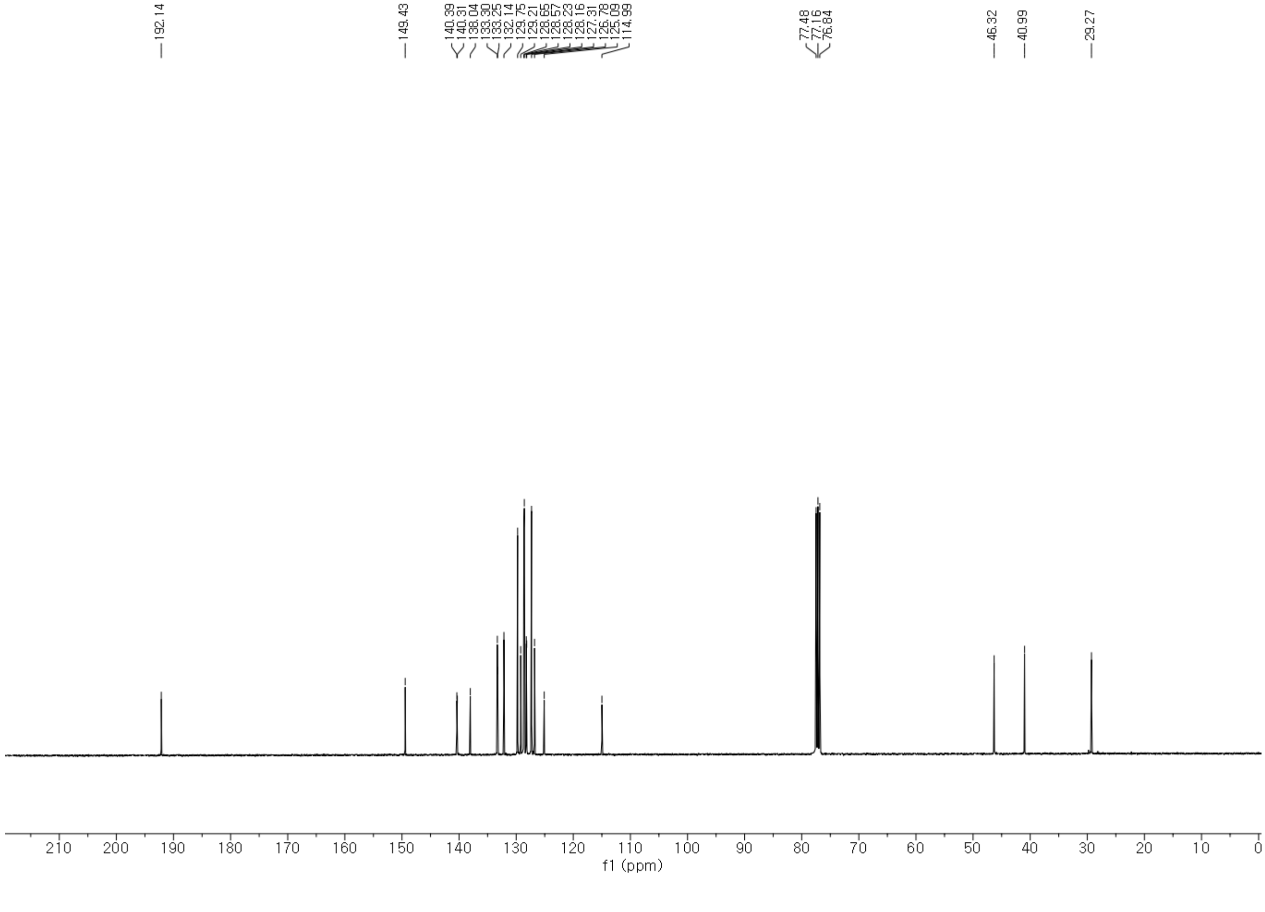

^{1}H NMR (400 MHz) in $CDCl_3$

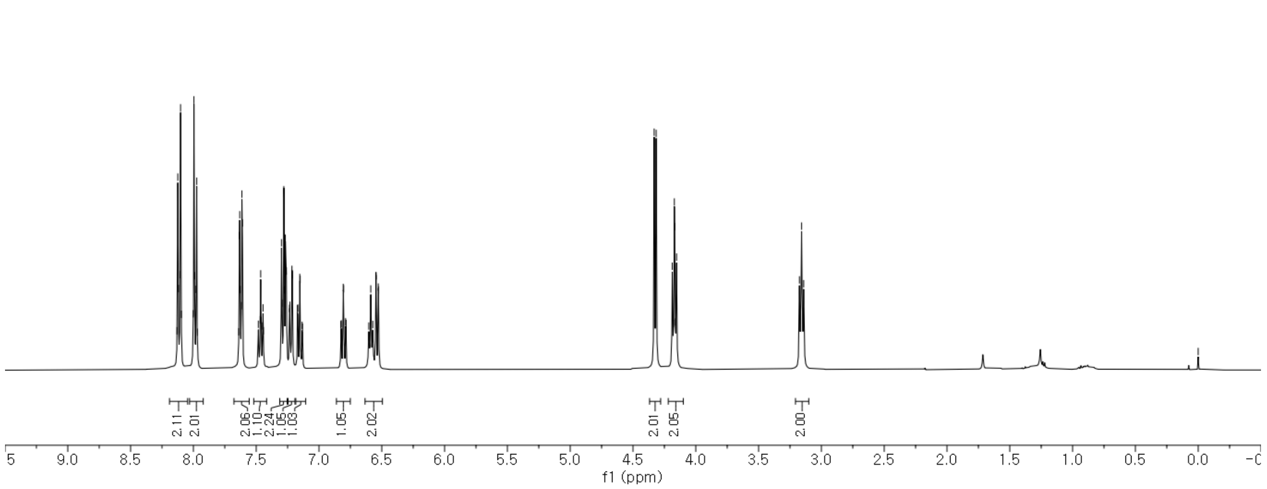

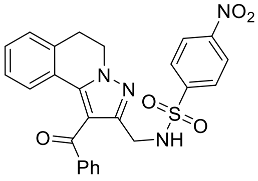

4ar

^{13}C NMR (100 MHz) in $CDCl_3$

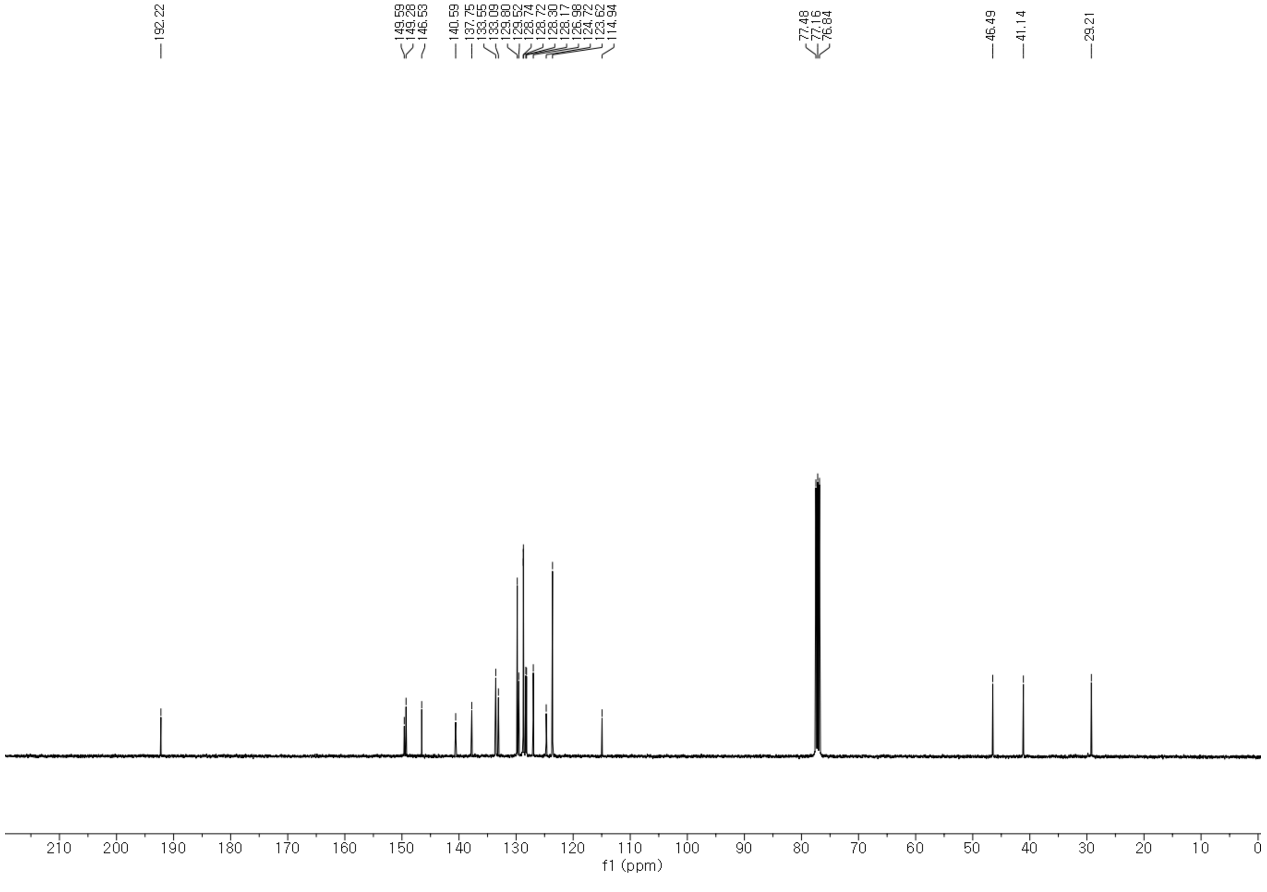

^{1}H NMR (400 MHz) in $CDCl_3$

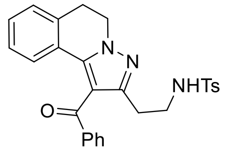

4as

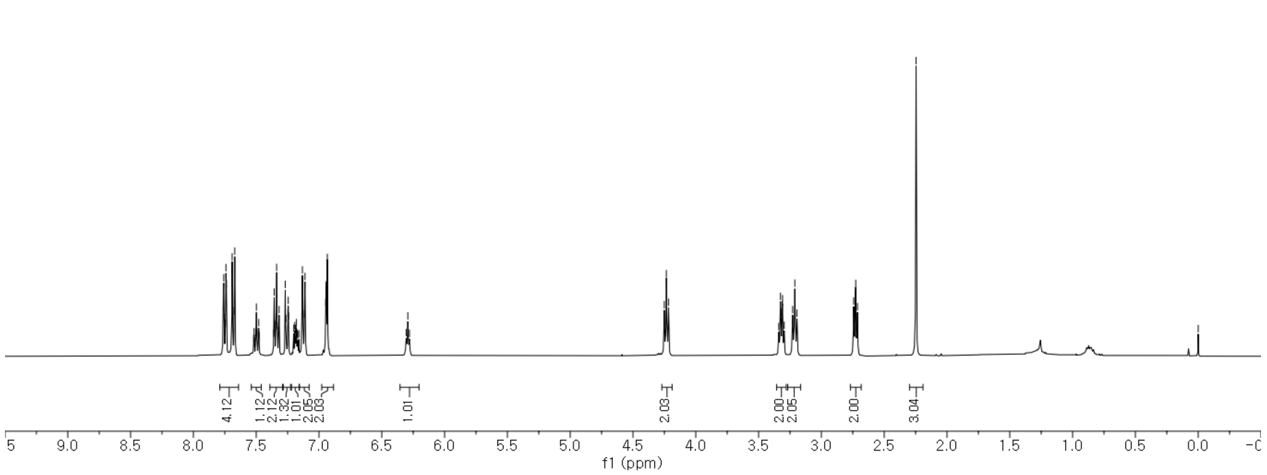

^{13}C NMR (100 MHz) in $CDCl_3$

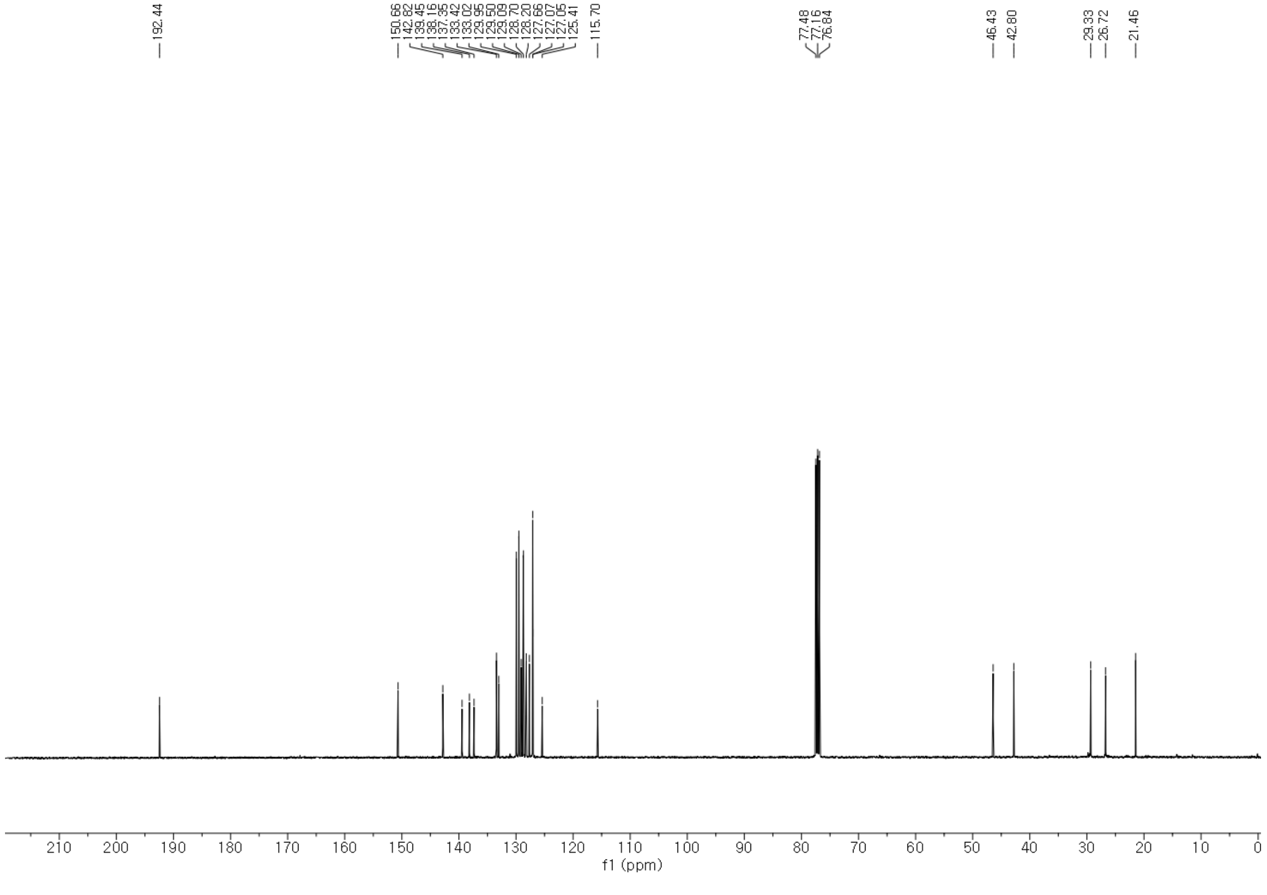

^{1}H NMR (400 MHz) in $CDCl_3$

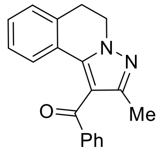

4at

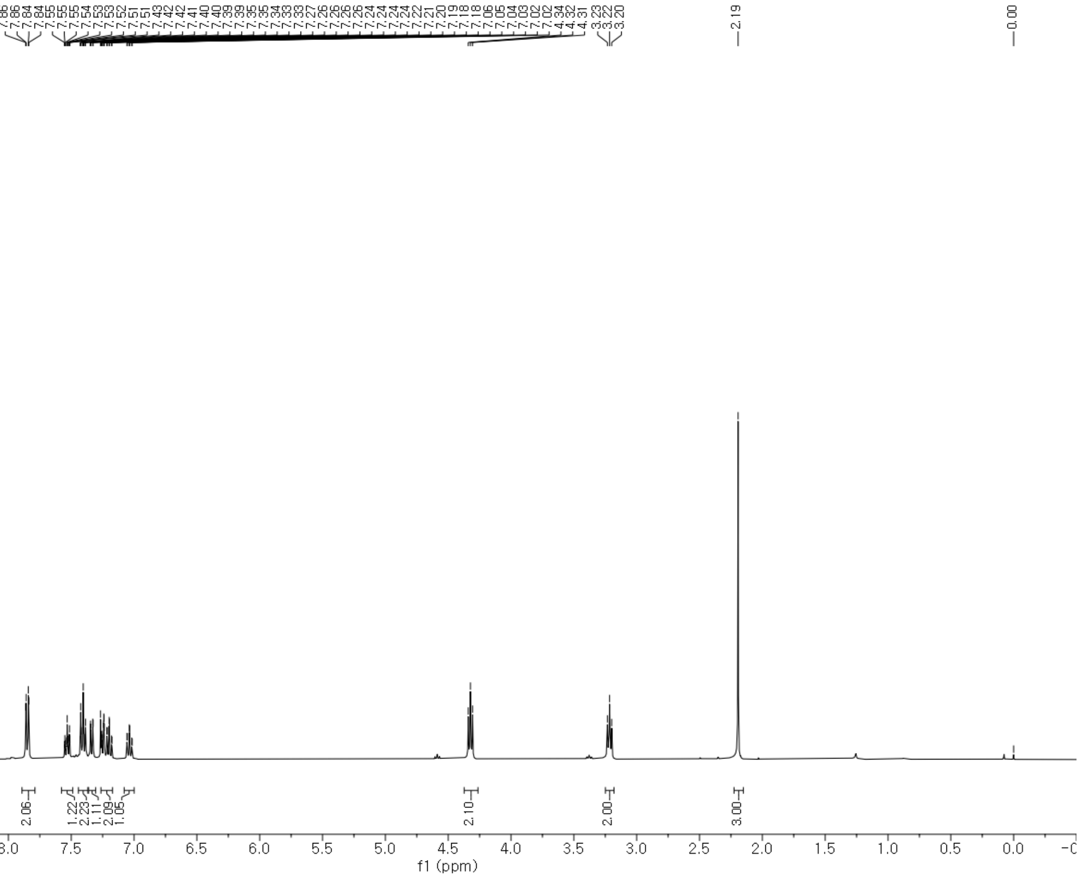

^{13}C NMR (100 MHz) in $CDCl_3$

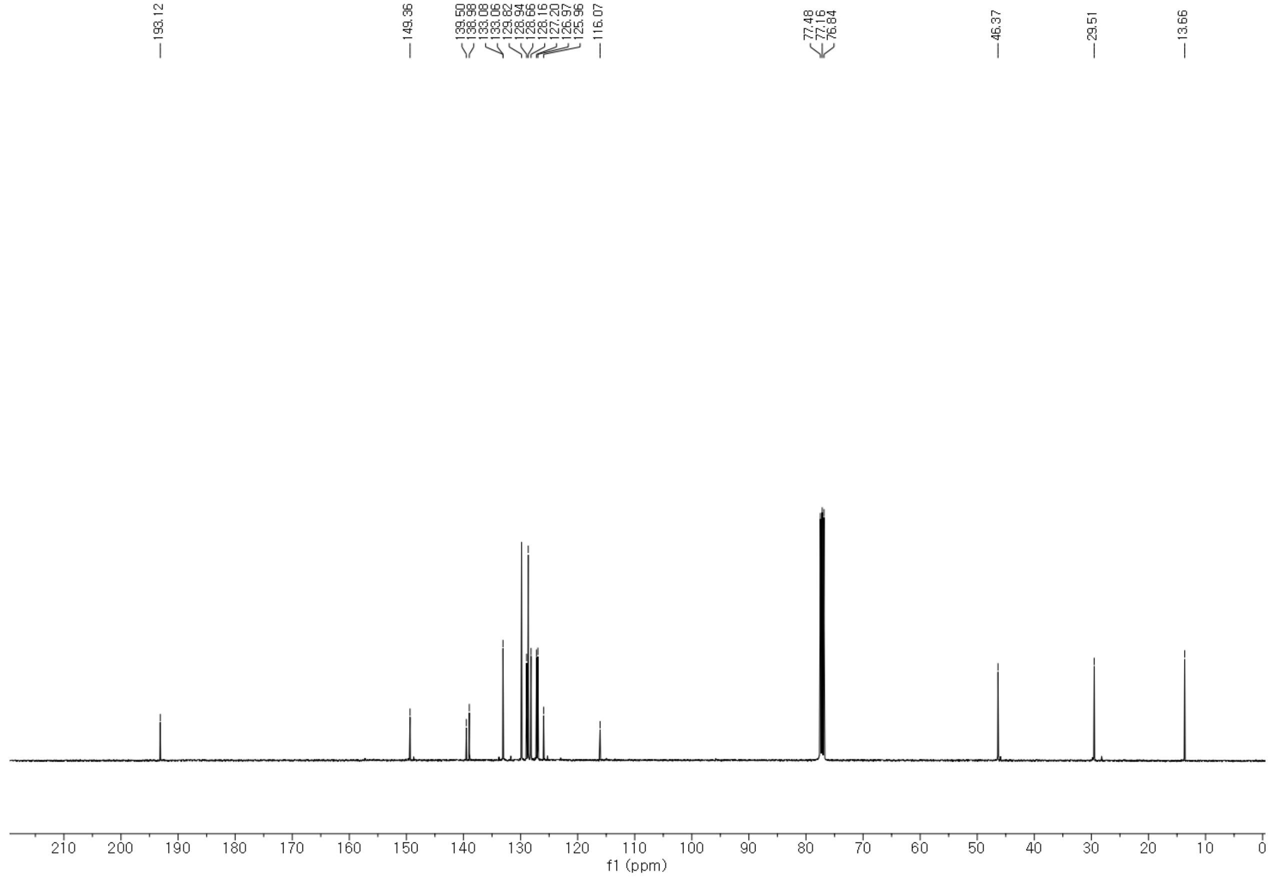

^{1}H NMR (400 MHz) in $CDCl_3$

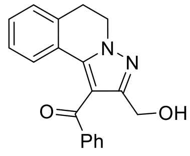

4au

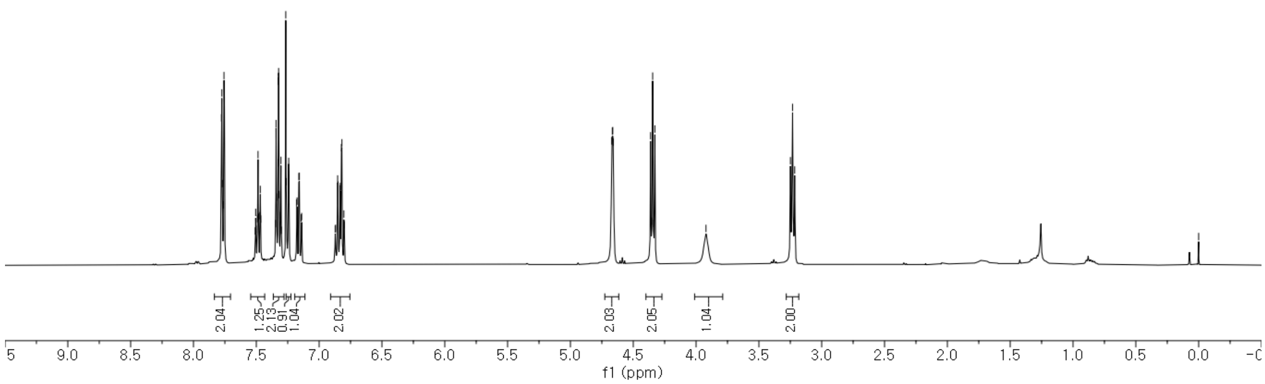

^{13}C NMR (100 MHz) in $CDCl_3$

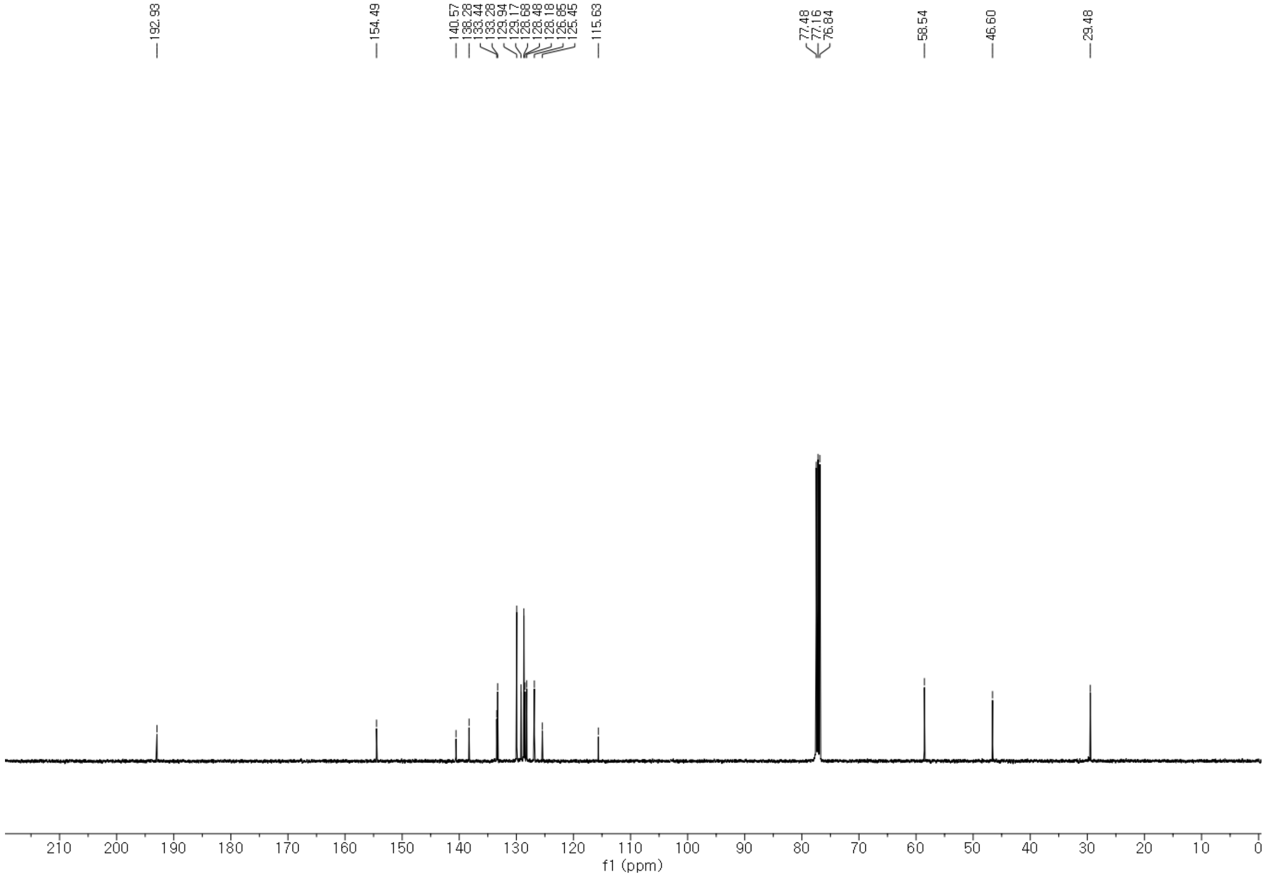

^{1}H NMR (400 MHz) in $CDCl_3$

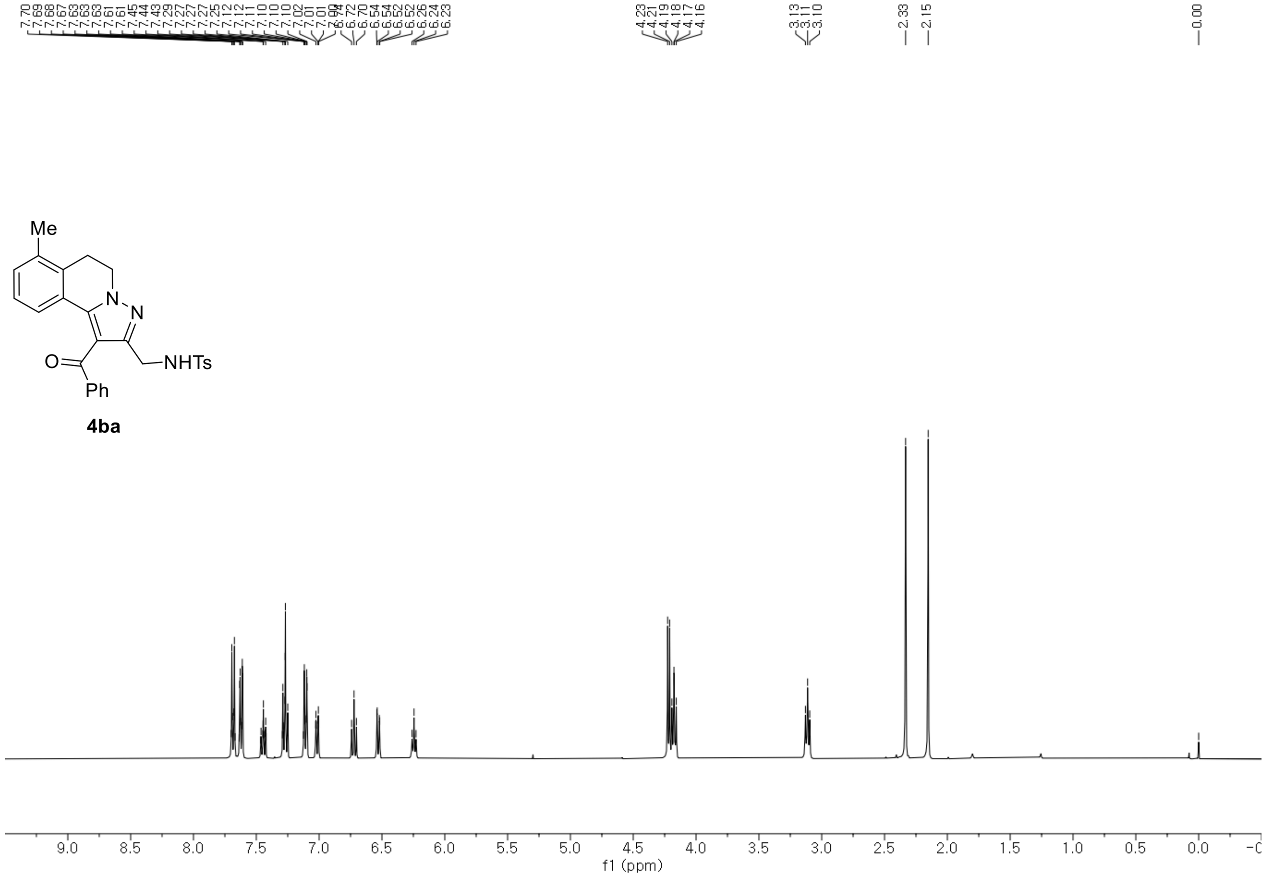

^{13}C NMR (100 MHz) in $CDCl_3$

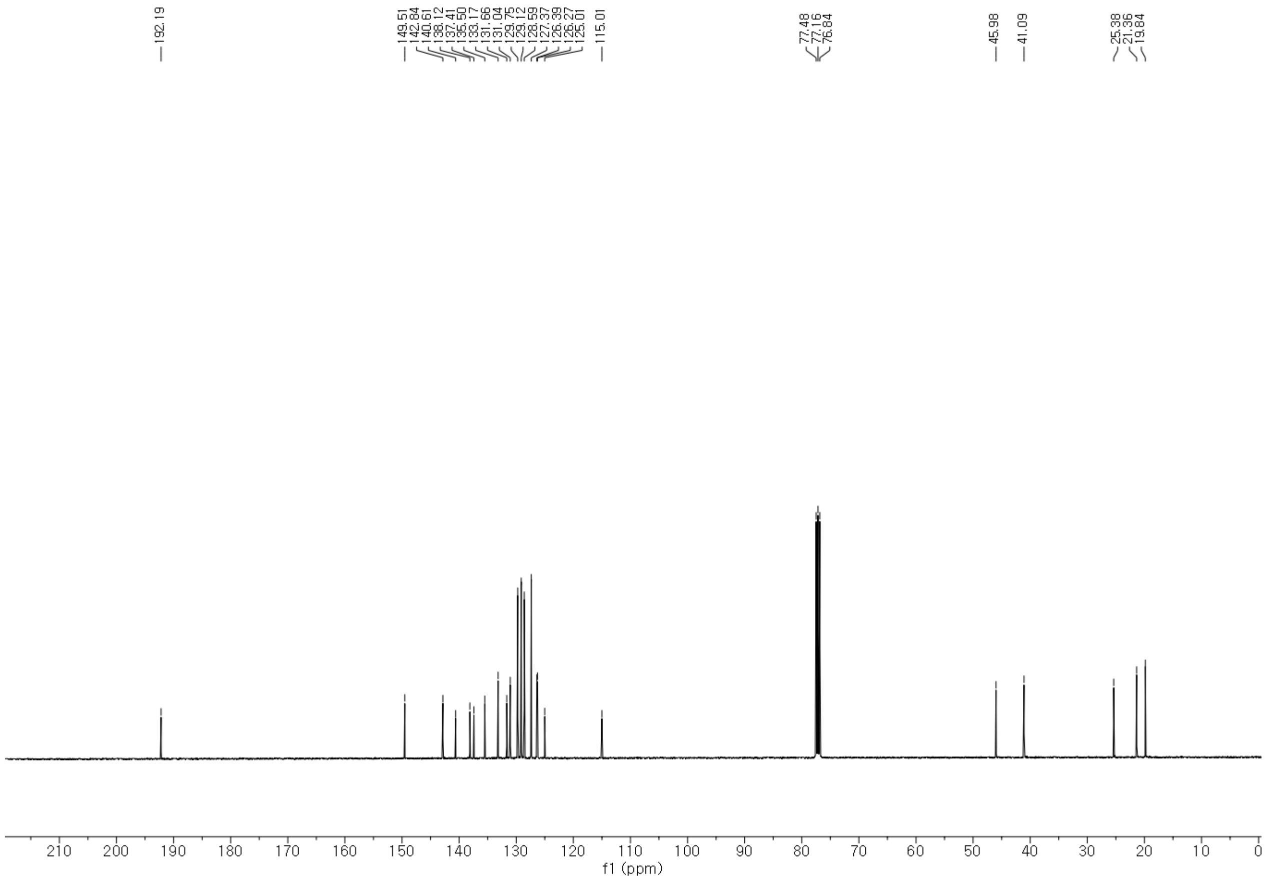

^{1}H NMR (400 MHz) in $CDCl_3$

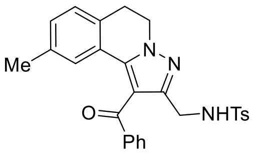

4ca

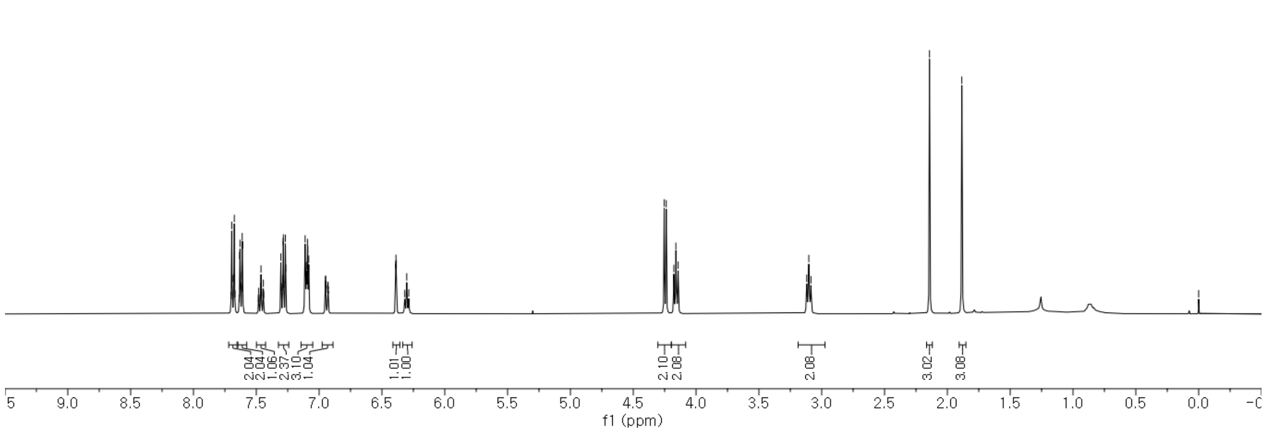

^{13}C NMR (100 MHz) in $CDCl_3$

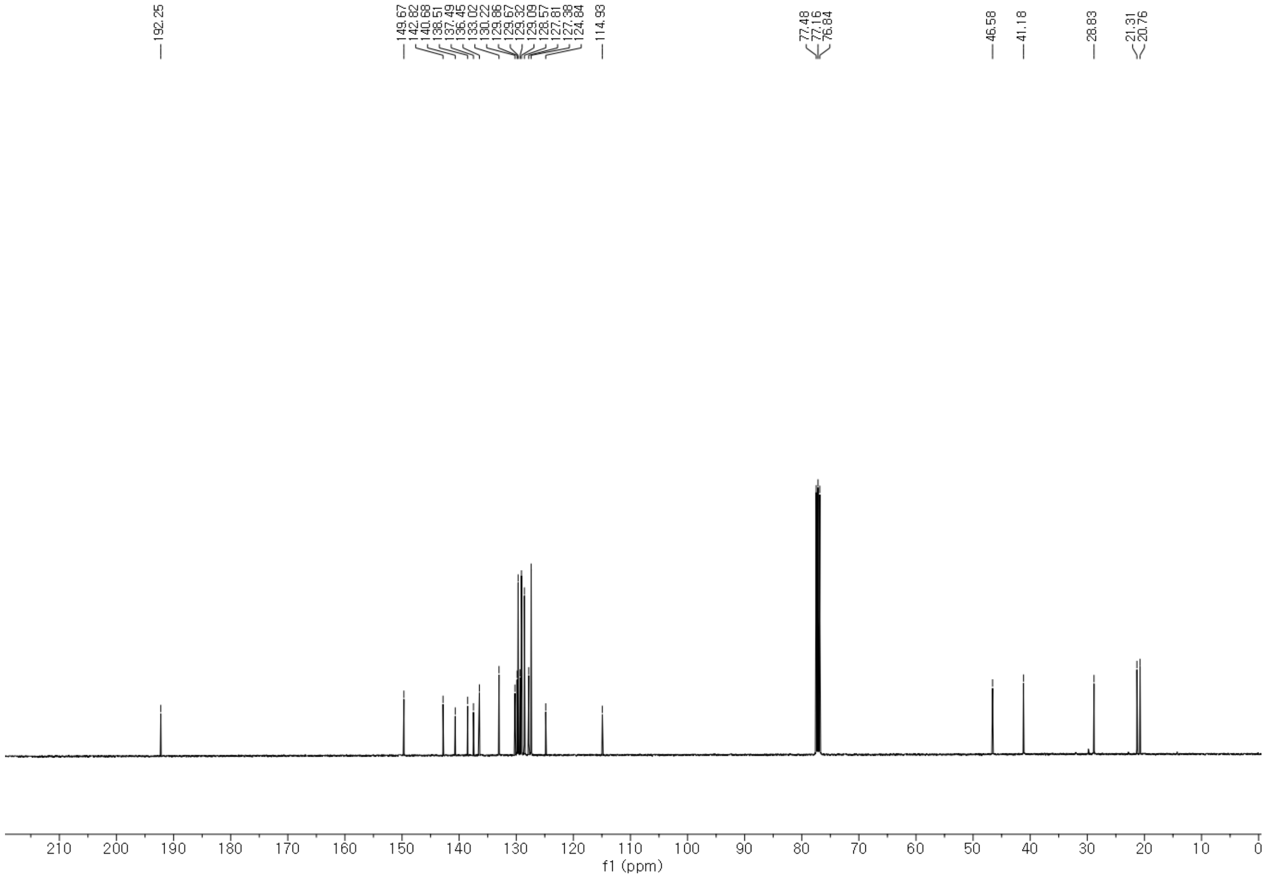

^{1}H NMR (400 MHz) in $CDCl_3$

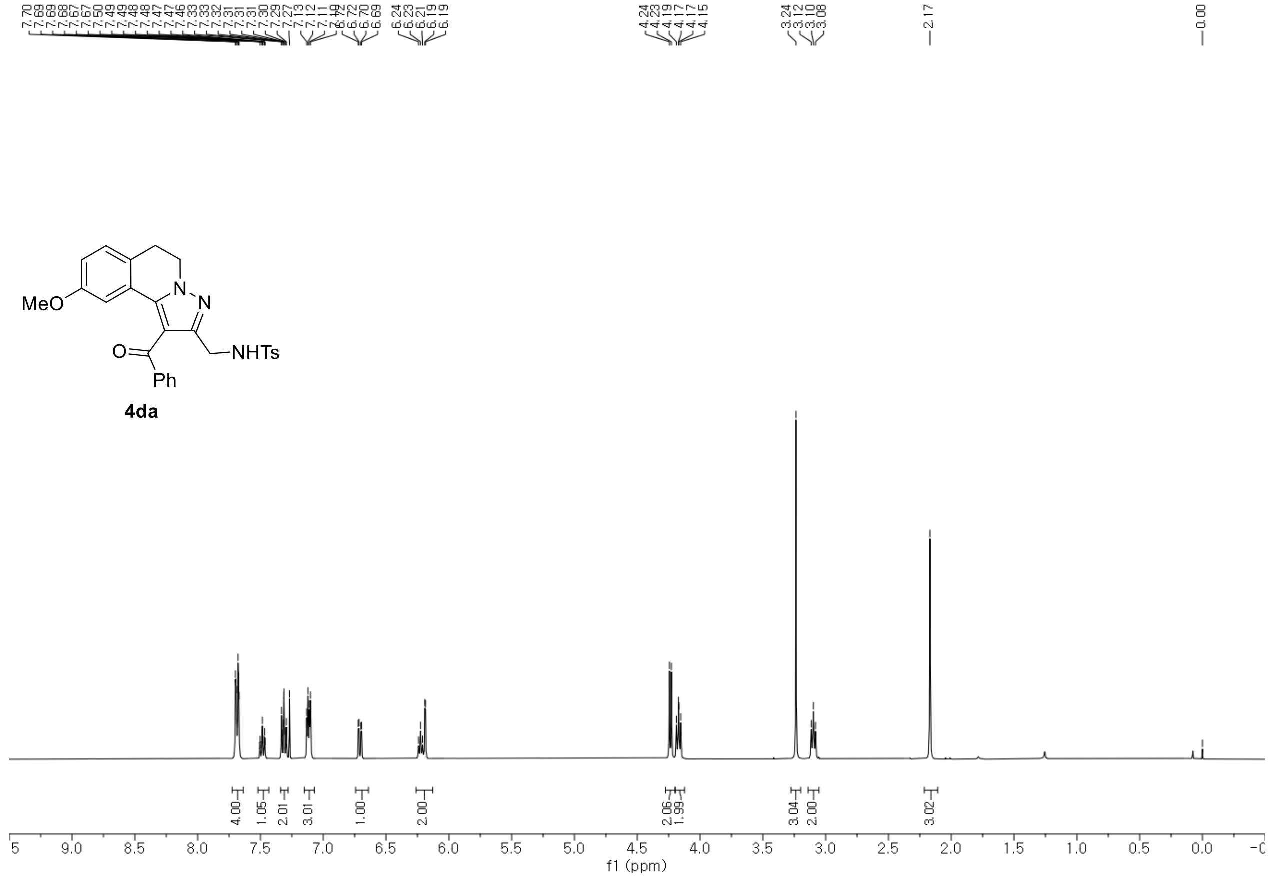

^{13}C NMR (100 MHz) in $CDCl_3$

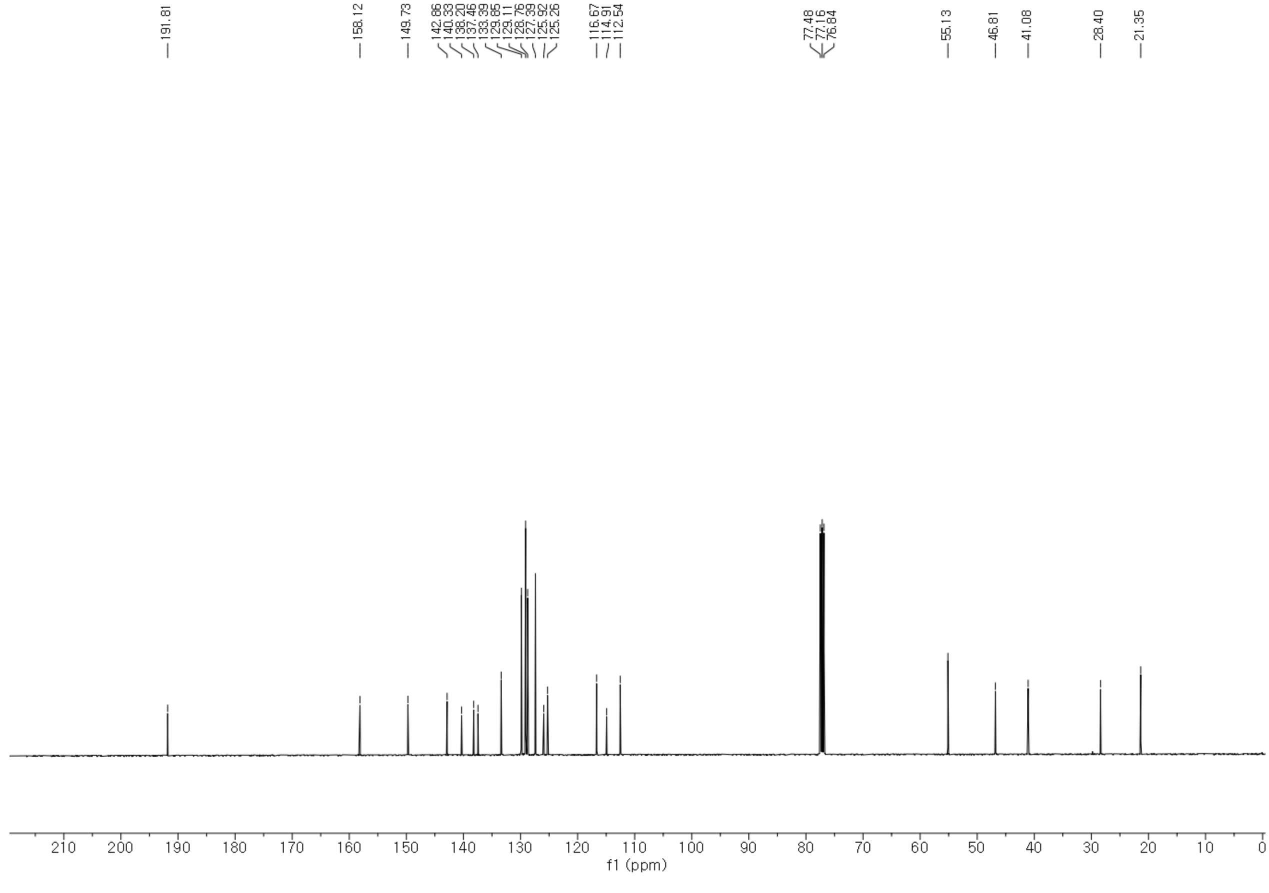

^{1}H NMR (400 MHz) in $CDCl_3$

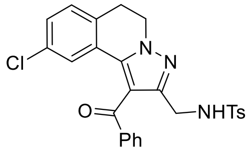

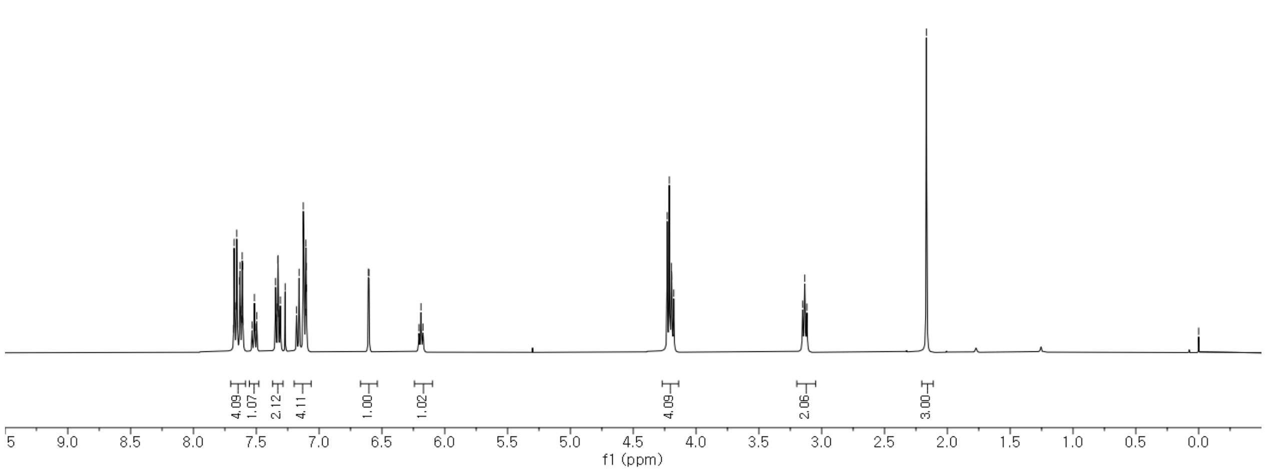

4ea

^{13}C NMR (100 MHz) in $CDCl_3$

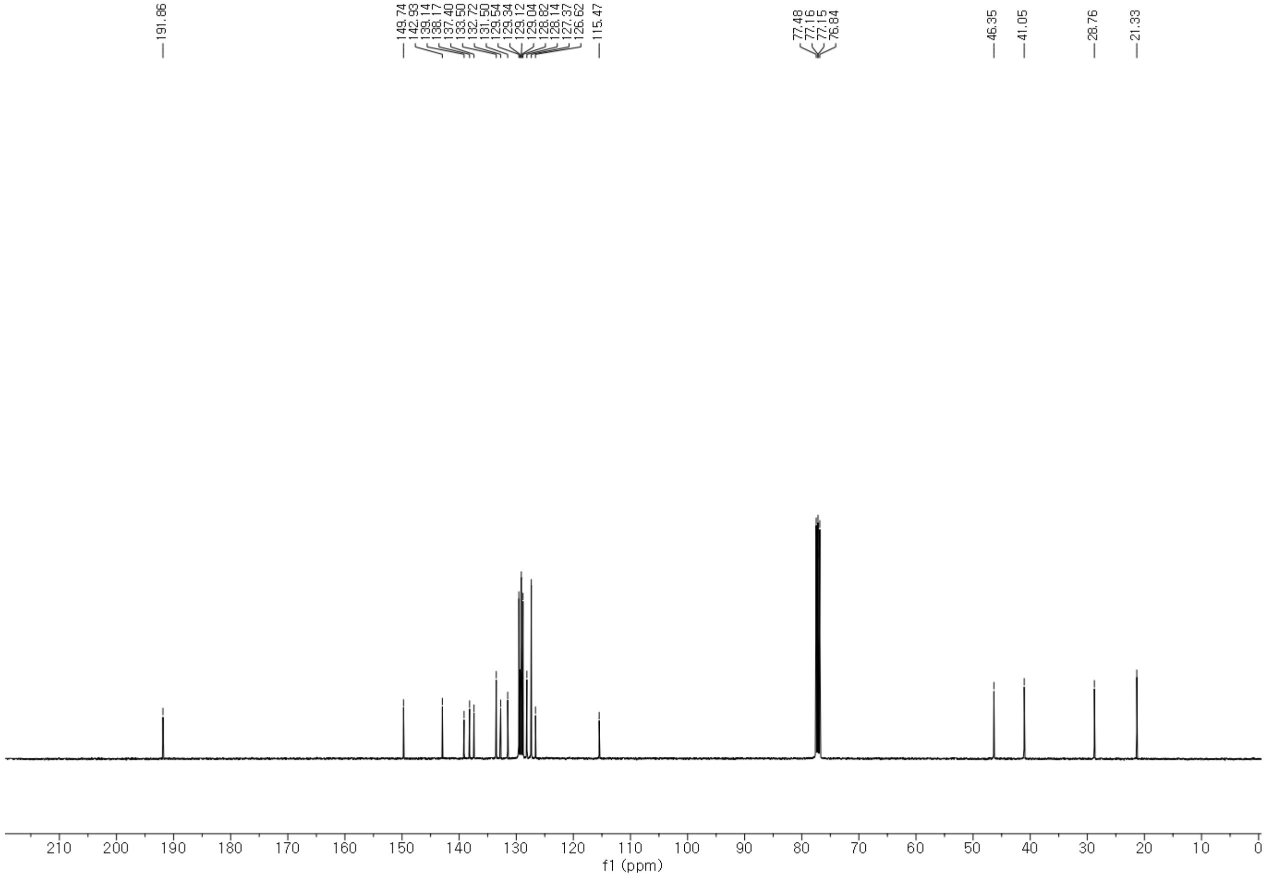

^{1}H NMR (400 MHz) in $CDCl_3$

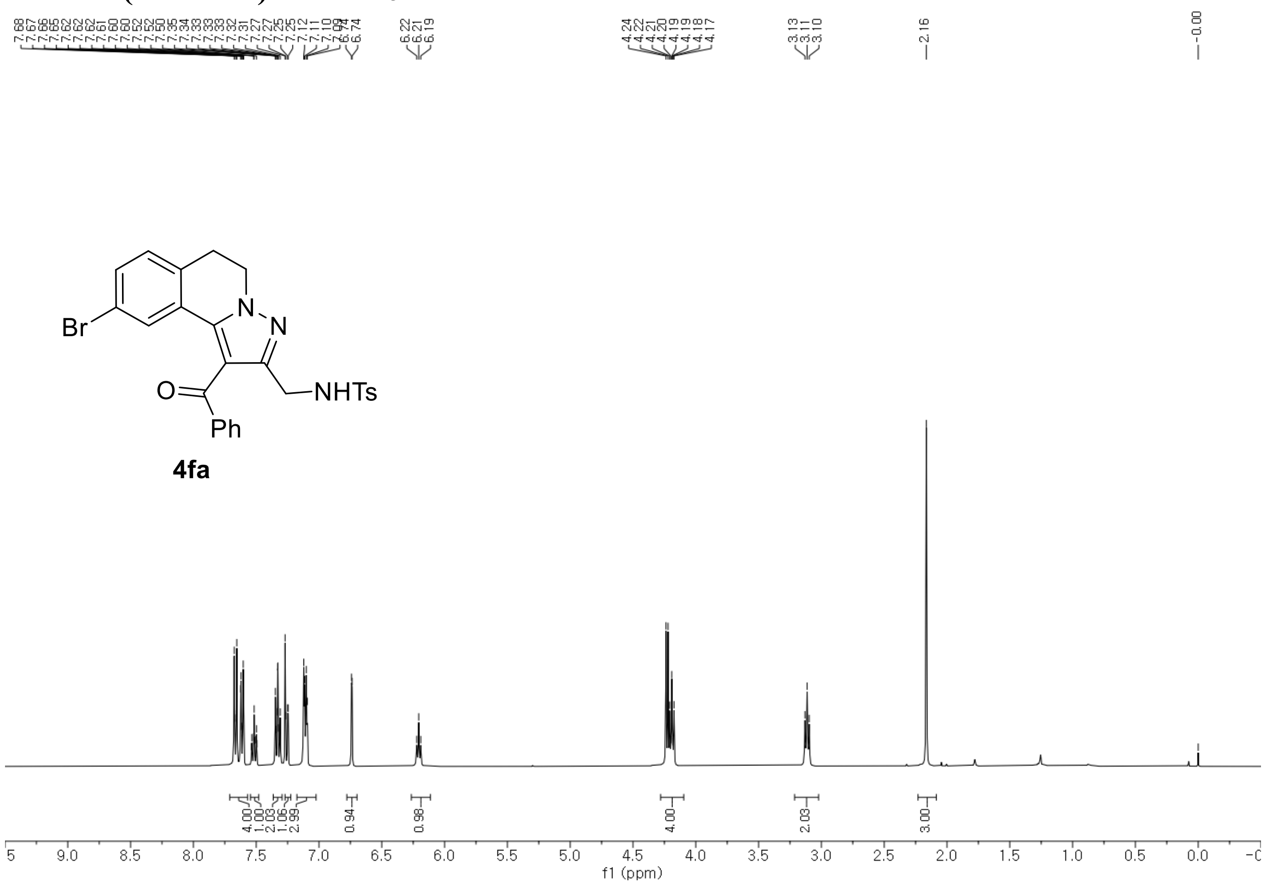

^{13}C NMR (100 MHz) in $CDCl_3$

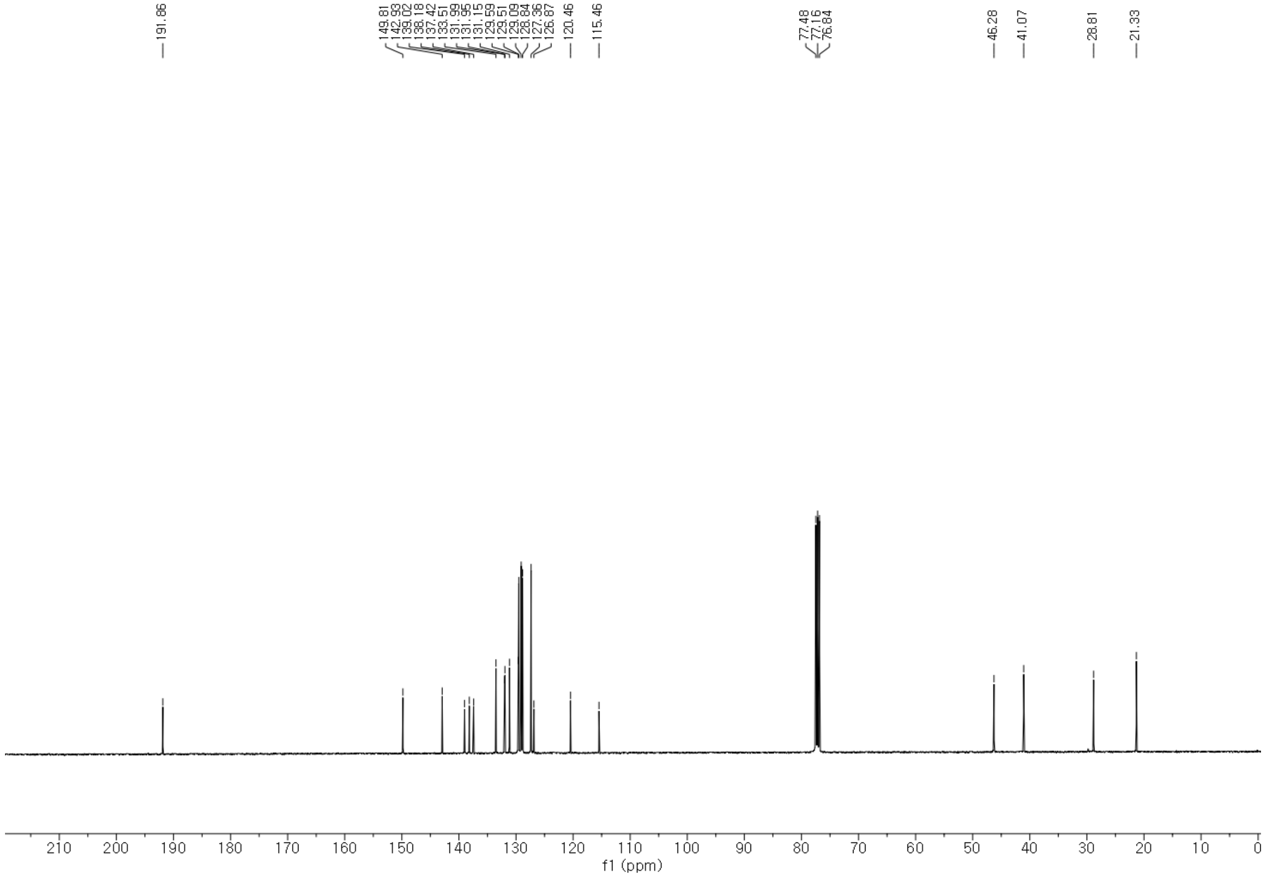

Supplement: Supplementary file 1 [file molecules-28-03710-s001.zip › molecules-2353494-supplementary.pdf]
